# Driving Assessment for Persons with Dementia: How and when?

**DOI:** 10.14336/AD.2022.1126

**Published:** 2023-06-01

**Authors:** Lara Camilleri, David Whitehead

**Affiliations:** ^1^Saint Vincent De Paul Long Term Care Facility, L-Ingiered Road, Luqa, Malta.; ^2^Department of Gerontology, University Hospital of Wales, Heath Park, Cardiff, CF14 4XW, United Kingdom.

**Keywords:** dementia, driving, behaviors, assessments

## Abstract

Dementia is a progressive neurodegenerative disease leading to deterioration in cognitive and physical skills. Driving is an important instrumental activity of daily living, essential for independence. However, this is a complex skill. A moving vehicle can be a dangerous tool in the hand of someone who cannot maneuver it properly. As a result, the assessment of driving capacity should be part of the management of dementia. Moreover, dementia comprises of different etiologies and stages consisting of different presentations. As a result, this study aims to identify driving behaviors common in dementia and compare different assessment methods. A literature search was conducted using the PRISMA checklist as a framework. A total of forty-four observational studies and four meta-analyses were identified. Study characteristics varied greatly with regards to methodology, population, assessments, and outcome measures used. Drivers with dementia performed generally worse than cognitively normal drivers. Poor speed maintenance, lane maintenance, difficulty managing intersections and poor response to traffic stimuli were the most common behaviors in drivers with dementia. Naturalistic driving, standardized road assessments, neuropsychological tests, participant self-rating and caregiver rating were the most common driving assessment methods used. Naturalistic driving and on-road assessments had the highest predictive accuracy. Results on other forms of assessments varied greatly. Both driving behaviors and assessments were influenced by different stages and etiologies of dementia at varying degrees. Methodology and results in available research are varied and inconsistent. As a result, better quality research is required in this field.

## INTRODUCTION

An average of 1.35 million people dies each year from road traffic accidents (World Health Organization, 2018 Global Status Report on Road Safety, available online from www.who.int/violence_injury_prevention/road_safety_status/2018/English-Summary-GSRRS2018.pdf?ua=1). With an ageing population, the number of elderly drivers on the road is increasing. In fact, drivers aged more than 85 years doubled in amount from 1998 to 2013 [1]. Driving plays an important role in providing independence and mobility. In fact, a systematic review showed that driving cessation in the elderly is associated with a perceived reduced quality of life, greater risk of depression, social isolation, deteriorating physical and cognitive function and a higher institutionalization risk [2].

However, driving is a complex task requiring various skills including physical, sensory, behavioral, and cognitive ones. Old age is associated with co-morbidities and polypharmacy which might affect driving capacity. Table 1 provides a list of these which includes dementia [3]. Dementia affects around 50 million people worldwide [4]. It is a neurodegenerative disease affecting cognitive, physical, and behavioral abilities. It has multiple etiologies, all comprising different signs and symptoms with different rates of progression. As a result, the effects of dementia on driving can vary from one person to the other. This makes driving assessment in dementia quite challenging. Basic knowledge of the neurophysiology and mechanisms of both driving and dementia is essential to help us understand how dementia can impede driving, which in turn will guide us in the assessment process.

**Table 1:** Driver’s intrinsic factors that can impact driving competence and performance.

Neurological	Musculoskeletal	Cardiovascular/ Respiratory	Medications / Toxins
**Epilepsy** **Movement disorders including Parkinson’s Disease** **Huntington’s disease** **Stroke** **Certain psychiatric conditions,** **Spinal cord injuries,** **Peripheral neuropathy,** **Sensory impairment especially visual,** **Autonomic dysfunction,** **Sleep disorders,** **Vertigo,** **Cognitive impairment.**	Any form of arthritis that can limit range of motion and dexterity, Fractures or deformities, Amputations, Sarcopenia, Myositis	Arrhythmias Syncope or pre-syncope, Severe valvular pathology, Cardiac ischaemia, Reduced cardiac output as present in heart failure, Carotid stenosis Hypotension, Obstructive Sleep Apnoea	Alcohol, Benzodiazepines, Antipsychotics, Anticholinergics, Antihistamines, Antihypertensives, Antianginals, Antiparkinsonian medications, Hypoglycaemic agents

### 1.1 The Driving Process

Driving is a complex task involving the co-ordination of various cognitive, motor, and perceptual skills. Moreover, driving is a dynamic task influenced by both intrinsic and extrinsic factors [5]. Intrinsic factors refer to the driver’s individual factors including neurological and cognitive abilities, motor function, medications, co-morbidities, psychology, and behaviors. External factors are related to the outside environment and include the type and quality of vehicle being used, road conditions, weather conditions, traffic, and other environmental distractions (Leung J (2004), ‘Psychological predictors of fitness to drive’ University of Wollongong Australia; Available online from Google Scholar at https://ro.uow.edu.au/theses/2135). In fact, various theoretical models of driving capacity were developed to better understand these interactions (Supplementary Table 1) [6-9]. However, none of these models are representative of both driving competence and performance [5].

With the development of science and technology, the neural basis of driving has been investigated. Navarro J, Reynaud E and Osiurak F (2018) conducted a meta-analysis of studies exploring functional neuroimaging data of healthy drivers on a simulator and identified a core driving circuit involving the following brain regions: Cerebellum, extra-striate cortex in the occipital lobe, middle temporal gyrus in the temporal lobe, precuneus in the parietal lobe, the precentral gyrus, superior and middle frontal gyri located in the frontal lobe, the anterior insula, the posterior cingulate gyrus and the dorsomedial thalamus [7]. They also explored the MRI images in relation to the three-level hierarchal model. The sensory and motor areas of the brain were activated during the strategic component. The middle frontal area and middle temporal gyri were activated during the tactical stage of driving. The extrastriate cortex, anterior cerebellum and the thalamus were activated during the operational stage of driving [7].

### 1.2 Dementia and cognition

Dementia is a cause of cognitive impairment but not all cases of cognitive impairment are due to dementia [10]. This article will only focus on the relationship between driving and cognitive impairment secondary to dementia.

Cognition consists of multiple domains. Harvey PD (2019) named seven cognitive domains as described in Table 2 [11-12]. Dementia can occur due to different pathophysiological processes which can affect different cognitive domains and therefore lead to a spectrum of clinical manifestations. As a result, diagnostic criteria were developed to identify the presence of dementia and subsequently the etiology and stage. The most common causes of neurodegenerative dementia include Alzheimer’s disease (AD) followed by Lewy Body Dementia (LBD) followed by Frontotemporal Dementia (FTD). The most common cause of non-neurodegenerative dementia is vascular dementia (VaD). Vad accounts for 15-35% of all dementias making it the second most common cause of dementia following AD [13]. Table 3 describes and compares these subtypes [13-18]. Moreover, multiple etiologies can co-exist resulting in mixed dementia with the combination of AD and VaD being most common [13].

To date, there is no effective disease-modifying treatment for dementia, so the course of disease is progressive in nature irrespective of the underlying pathophysiology [19]. In fact, dementia is often classified into three stages: mild, moderate and severe. Dementia disease severity is associated with worsening cognitive and functional abilities as well as increased behavioral problems [19]. Moreover, it might be more difficult to differentiate between dementia subtypes in later stages since symptoms often overlap as neurodegeneration becomes more diffuse [20]. This stresses the importance of early dementia diagnosis for prognostication and management planning. In fact, several tools have been devised to assess cognitive and functional abilities to help diagnose and grade dementia.

**Table 2:** Cognition - Components and Neuroanatomy.

Cognitive domain	Description	Components	Associated neuroanatomy
**Attention**	Ability to filter relevant and irrelevant information from various stimuli in order to maintain concentration and avoid distractibility.	Selective attention Sustained attention Vigilance Arousal	Prefrontal cortex namely cingulate gyrus and amygdala. Ascending reticular activating system. Frontal subcortical network Parietal region.
**Executive function**	A process utilising multiple cognitive domains for problem solving, planning, judgement and task performance.	Initiation Planning Cognitive flexibility Self-monitoring	Anterior cingulate gyrus Dorsolateral prefrontal cortex
**Memory**	Most complex cognitive domain. Responsible for acquiring, storing and retrieving new and learnt information from various stimuli. Also involved in learning.	Working memory Episodic memory Encoding Storage Recall Semantic memory Long- and short-term memory Verbal and visual memory Prospective memory	Reticular activating system Prefrontal and frontal cortex Parietal cortex Temporal lobe Amygdala
**Sensation and Perception**	Also known as visuospatial skills. Refers to the brain’s ability to detect and process any sensory stimulus which can be used to identify objects, sounds and other stimuli compared to their orientation in space	Perception Construction	Occipital lobe Temporal lobe Parietal lobe
**Motor skills and construction**	The brain’s ability to carry out motor skills including dexterity, balance, motor speed and reaction time. It also includes the ability to copy and reproduce drawings of objects.	Speed Strength Dexterity Co-ordination Apraxia	Premotor and motor cortex of frontal lobe. Somatosensory area of the parietal lobe. Cerebellum Brain stem
**Processing speed**	It effects the rate at which a simple or complex task can be performed.	Linked to all other cognitive domains	Frontal lobe Parietal lobe
**Language**	This involves the brain’s ability to both understand and produce verbal information.	Expressive Receptive Repetition	Motor and prefrontal cortices Wernicke’s area Broca’s area

### 1.3 Driving with dementia

Driving competence and driving performance are two separate concepts since various dynamic intrinsic and extrinsic factors can affect the final outcome [5]. As a result, driving assessment in dementia may comprise of various components [21]. The aim of driving assessment in drivers with dementia is to balance the benefits of driving and the risks of driving cessation, versus the risk of continued driving.

#### Mobility and driving cessation

A private vehicle is the most common mode of transport amongst elderly people in the US with only 3% of older people using public transport [22]. The importance of private transportation became more evident during the global pandemic. In fact, risk factors for COVID-19 related mortality include age above 65 years, male sex and the presence of co-morbidities [23]. As a result, public transportation may be dangerous for elderly people during a pandemic, due to an increase infection risk in crowded subways and buses. Moreover, if the patient is the main driver and their caregivers are unable to drive, the revocation of their driving license may have dire consequences. It may lead to isolation and impede access to essential resources with potential neglect and/or caregiver burden [24].

**Table 3:** Dementia Subtypes - Pathophysiology and clinical manifestations.

	Alzheimer’s Disease (AD)	Frontotemporal Dementia (FTD)	Lewy Body Dementia (LBD)	Vascular Dementia (VaD)
**Pathophysiology**	Accumulation of beta amyloid plaques, tau proteins and neurofibrillary tangles.	Transactive responsive DNA-binding, tau protein accumulation, and fused-in-sarcoma.	Accumulation and deposition of Lewy bodies (alpha-synuclein aggregates) in neurons.	Cerebrovascular events including ischaemic, embolic or haemorrhagic strokes, small vessel disease and cerebral amyloid angiopathy
**Effected Brain Region**	Parietal and temporal areas	Frontal and anterior temporal areas	Basal ganglia and limbic system	Varies according to vascular territory involved.
**Effected Cognitive Domains**	Memory is the first domain to be affected but may also affect visuospatial and language domains in certain AD variants.	Executive function and social cognition effected first in the behavioural variant and language effected first in the language variant.	Attention and alertness are the first cognitive domains to be affected, followed by executive and visuospatial functions.	Vary according to vascular territory involved. Reduced processing speed, memory and language impairment in small vessel disease.
**Diagnostic Criteria**	National Institute on Aging and the Alzheimer’s Association (NIA-AA) criteria. Divide AD as Probable or Possible based on clinical and/or radiological/ biochemical criteria.	Clinical criteria for behavioural variant FTD revised by the International Consensus Criteria in 2011. Diagnostic criteria for FTD- associated aphasic syndromes or primary progressive aphasia	Revised DLB Consortium Consensus Criteria. Divide LBD as Probable or Possible based on clinical and/or radiological/ biochemical criteria.	Multiple diagnostic criteria are available. The NINDS-AIREN criteria have the highest specificity and are most commonly used in research. They divide VaD as Possible, Probable, Definite and Mixed based on both clinical and radiological / histological criteria.
**Clinical Manifestations/Classification**	Slowly progressive decline. Mean survival rate of 10-12 years. Two main types by NIA-AA criteria: Amnestic presentation: Inability to learn or recall new information. Nonamnestic presentation - language, visuospatial or executive function are the prominent symptoms.	More common than LBD in people aged less than 65 years but still less common than AD. Symptoms depend on the subtype: Behavioural variant FTD: Executive function and social cognition affected resulting in personality changes and behavioural problems. Language variant FTD (progressive primary aphasia)	Progressive Cognitive decline. Parkinsonism Hallucinations REM sleep disorders Fluctuating alertness. Sensitivity to antipsychotics Postural instability Severe autonomic dysfunction Hyper or hypo-osmia Delusions Anxiety / depression Cognitive and motor symptoms occur <1year apart	Cognitive and motor neurological symptoms vary according to the vascular territory involved. Disease progression may be of acute onset with stepwise deterioration if repeated vascular events occur or slowly progressive with insidious onset in small vessel disease.

The true proportion of drivers with dementia is difficult to assess. A longitudinal study concluded that approximately 30% of drivers aged over 75 years, have dementia [25]. This study also showed that the degree of cognitive impairment was associated with a higher percentage of driving cessation over 3 years. A 12% cessation rate in those without dementia was identified, compared to 50% in those with mild dementia compared to >95% in those with moderate to severe disease. This implies that a substantial proportion of dementia patients still drove for a significant period post diagnosis. However, there are numerous factors that influence driving cessation in dementia. The PRODERM longitudinal study, examined these risk factors and identified the following: [26]

- Type of dementia, with DLB having the highest rate of cessation.
- More advanced stages of dementia and worse cognition,
- Higher ADL impairment,
- Female gender.

The PRODERM study also identified the main reason for cessation, which was caregiver concern (93.8%) as opposed to crashes (5.5%) and revocation of license (0.7%) [26]. A retrospective study also examined risk factors of driving cessation in dementia in 427 patients. Of these, 26% were still driving post diagnosis. This study also showed that in 40% of cases, the decision to stop was made by the patients themselves, in 29% it was made based on advice from relatives and in 25% based on advice from physicians [27].

**Table 4:** Laws on Driving in Dementia - Comparison between countries.

Country	Legal Authority	Driving Assessment Guidelines Used	Licence Validity and Renewal Period	Additional Tests Required for Drivers with Dementia	Legal obligation to report	Absolute Indication for Revocation of Licence
**UK**	Driver and Vehicle Licensing Agency (DVLA)	At a glance Guide to the current Medical Standards of Fitness to Drive	1 year	Visual Acuity Driving test Functional testing Medical evaluation Cognitive testing, Neuropsychological assessment and written knowledge test.	Driver’s obligation (Self- report)	Advanced dementia, specifically those with poor short-term memory, disorientation, lack of insight and judgement.
**Germany**	Federal Motor Transport Authority Driving License Division	Expert Guidelines on Driver’s Aptitude 2000 Assessment Guidelines for Drivers’ Suitability	Not specified	Cognitive testing, Neuropsychological assessment and written knowledge test.	Not specified	Advanced dementia
**France**	General Police Directorate License Office	None	15 years	Medical examination from accredited physicians in assessing driving competence, Cognitive testing, Neuropsychological assessment and written knowledge test.	Medical certificate issued by physician but reporting duty relies on the driver	Advanced dementia
**Italy**	Department of Land Transport Provincial Office of Motor Vehicles Local health authority	Guidelines Medical Services-Legal Territorial	50-70 years = 5 years 70-80 years = 3 years >80 years = 2 years	Visual Acuity Functional testing Medical evaluation Cognitive testing, Neuropsychological assessment and written knowledge test.	Medical certificate issued by physician but reporting obligations rely on the driver.	Advanced dementia
**USA**	National Highway Traffic Safety Administration (NHTSA) Department of Motor Vehicles (DMV)	The Physician’s Guide to Assessing and Counseling Older Drivers issued by the American Medical Association and the National Highway Traffic Safety Administration (NHTSA).	2 to 6 years (varies according to state)	Visual Acuity Driving test Functional testing Medical evaluation Cognitive testing, Neuropsychological assessment and written knowledge test.	Varies between states but physician reporting is specified in some states.	Advanced dementia
**Canada**	Canadian Council of Motor Transport Administration (CCMTA)	CCMTA Medical Standards for Drivers in 2020.	6 to 12 months	Visual Acuity Functional testing Medical evaluation Cognitive testing, Neuropsychological assessment and written knowledge test.	Physician’s obligation to report in all territories with additional self-reporting obligations in some territories.	Advanced dementia
**Australia**	Austroads & National Transport Commission (NTC)	Assessing Fitness to Drive for Commercial and Private Vehicle Drivers 2016 (AFTD)	1 year	Visual Acuity Driving test Functional testing Medical evaluation Cognitive testing, Neuropsychological assessment and written knowledge test.	Driver’s obligation (Self- report)	Advanced dementia
**Japan**	Metropolitan Police Department Driver License Center	Medical certificate description guidelines on dementia for doctors	3 years	Cognitive testing, Medical evaluation and traffic safety education completion certificate	Medical certificate issued by physician but reporting obligations rely on the driver.	Any dementia diagnosis
**China**	Ministry of Public Security	Not specified	1 year	Medical evaluation	Driver’s obligation (Self- report)	Any dementia diagnosis
**South Korea**	Korean National Police Agency Korea Road Traffic Authority	Not specified	5 years	General physical and functional examination if hospitalised for over 6 months, otherwise a standard medical evaluation.	Driver’s obligation (Self- report)	Advanced dementia

#### Driving risk in dementia

The major safety concern involves motor vehicle crashes (MVC) which can result in injury and death to the driver, car passengers, other drivers, or pedestrians. However, there is conflicting evidence on MVC risk in drivers with dementia, namely owing to different methodologies and sample sizes used as well as selection bias [28]. For instance, a systematic review conducted by Man-Son-Hing M et al. (2007), exploring crash risk in drivers with dementia, made a distinction in the method used when analyzing results [29]. In fact, all the studies which used caregiver reporting to report the number of MVCs, concluded that patients with dementia had a higher crash rate when compared to controls without dementia. On the other hand, only one of the three studies which used state crash records to identify MVC risk, found a higher MVC rate in patients with dementia. In addition, people with dementia were more likely to stop driving than cognitively normal subjects [25]. This could result in reduced hours of milage and therefore reduced opportunities to have an MVC. On the other hand, studies exploring driving habits in dementia relied on self-reported milage which can be inaccurate [29-30].

Nine out of the twenty-three articles included in the systematic review conducted by Man-Son-Hing M et al. (2007), examined drivers with only early or mild stages of dementia. However, another systematic literature review, concluded that crash risk in drivers with AD, increased with higher disease severity, where a CDR score of more than 1 was associated with an unacceptably high crash risk compared to all groups of drivers [31]. On the other hand, there are numerous studies, which show a reduced crash rate in dementia, especially in those with more advanced stages. However, these studies do not take into account the milage driven [30]. A Cochrane review concluded that the majority of studies which analyzed crash rates in patients with dementia, were retrospective, therefore subject to recall bias [22]. However, a prospective study found a significant association between higher crash rates in AD drivers and impairment in visuomotor, executive function and memory on neurophysiological testing [32].

These cognitive skills are also essential for orientation and rout finding. As a result, crashes are not the only risk associated with driving in dementia since drivers may also become lost. In fact, a study conducted by Hunt LA, Brown AE and Gilman IP (2010), identified 207 articles over 10 years which reported drivers with dementia becoming lost while driving [33]. From these 207 reports, 32 drivers were found dead, 70 drivers were not found nor had uncertain outcome and 116 drivers were found alive. 4% of drivers who were found dead or not found/uncertain outcome had passengers present compared to 6% of those drivers who were found alive. In addition, drivers found more than 48 hours after becoming lost, were more likely to be found dead. It is important to note that drivers with dementia, who got lost and died, were driving to familiar locations. The letter conclusion was also achieved in a retrospective study conducted by Rowe MA et al. (2012) [34]. This study also concluded that most of these trips were authorized by the driver’s caregivers and the driver gained access of the car keys behind the caregiver’s back in only one third of cases. The causes of death were multiple, ranging from MVC, drowning after driving into bodies of water, parking in dangerous places and wandering out of the car. In fact, the majority of deaths occurred when drivers wandered off, became lost on foot and were exposed to the elements [33-34].

#### Driving laws for people with dementia

The increasing aging population and the co-morbidities associated with aging have led to the development of various driving guidelines specific to age, co-morbidities and medications. You Joung K, Hoyoung A, Binna K et al. (2017), conducted a comparative review analyzing driving regulations worldwide, concerning the elderly and those suffering from dementia [35]. Ten developed countries were reviewed as described in Table 4 [35] (2016 Austroads National Road Transport Commission Guidelines, available online from https://austroads.com.au/__data/assets/pdf_file/0022/104197/AP-G56-17_Assessing_fitness_to_drive_2016_amended_Aug2017.pdf.) (2020 National Safety Code Standard 6: Determining Driver Fitness in Canada, available online from https://ccmta.ca/web/default/files/PDF/National%20Safety%20Code%20Standard%206%20-%20Determining%20Fitness%20to%20Drive%20in%20Canada%20-%20February%202021%20-%20Final.pdf.) (2021 Driver and Vehicle Licensing Agency guidelines, available online from https://assets.publishing.service.gov.uk/government/uploads/system/uploads/attachment_data/file/965900/MIS828_interactive_020321_Final.pdf) (2016 Clinician’s guide to assessing and counselling older drivers, available online from https://www.dmv.virginia.gov/drivers/pdf/older.pdf.) All countries except China used co-morbidities rather than age itself as a contraindication for driving. The license renewal period also varies considerably between countries, ranging from 6 months to 15 years, with the majority not exceeding 5 years. Reporting obligations also vary between countries, with the majority putting the legal obligations on the drivers themselves [35]. However, the physician has an active role in the driving assessment process since all countries require at least a medical certificate for license renewal. On the other hand, countries like the USA put the reporting obligation on the physician themselves highlighting the importance of education on driving assessment amongst physicians (2016 Clinician’s guide to assessing and counselling older drivers, available online from www.dmv.virginia.gov/drivers/pdf/older.pdf).

There is a lack of consensus on driving guidelines with regards to assessment methods and license review periods. In fact, a Cochrane review found a lack of good quality studies on driving assessment in dementia dementia [22]. On the other hand, a dementia diagnosis does not necessarily mean that driving capacity is impaired [31].

License renewal often requires a medical certificate for fitness to drive. As a result, the initial assessment and identification of drivers at risk is often performed by the patient’s physician, most commonly the general practitioner. However, driving assessment is a complex task which may prove challenging to physicians. In fact, Adler D and Rottunda SJ (2011) conducted a survey to assess physician’s knowledge, attitudes and practices when addressing driving in patients with dementia [36]. A total of 239 physicians working with patients with dementia completed the survey. The majority of the study population included general practitioners. Of these, 58.6% broached the subject of driving competence with their patients compared to 41.4% who did not. Another scoping review also examined the approach of assessing fitness to drive in dementia in primary care [37]. This review concluded that most primary care physicians were not comfortable or confident in performing a driving assessment in patients with cognitive impairment. This review also explored the reasons physicians were not comfortable performing such a task.

The following reasons were identified:

- Lack of good quality training;
- Lack of knowledge on local laws and resources;
- Fear of losing the patient’s trust and compromise the doctor-patient relationship;
- Concern about the risk: benefit ratio of driving cessation;
- Uncertainty on the optimal time or stage of disease at which to perform the assessment.

The above studies stress the need for continuous education, which would empower physicians to be comfortable in addressing such a sensitive and complex topic. As a result, the main objective of this study is to carry out a review of available literature and evidence on driving assessment in dementia. The aims include the following:

1. To identify driving behaviors in dementia.
2. To assess whether the dementia stage and subtype influence driving capacity.
3. To identify different types of driving assessment tools used in patients suffering from dementia.
4. To assess the accuracy of different driving assessment tools at predicting driving capacity in different stages of dementia.
5. To assess the accuracy of different driving assessment tools at predicting driving capacity in different types of dementia.

## MATERIALS AND METHODS

### Design

The research question was identified using the PICO strategy. Literature reviews are an effective method to identify, criticize and summarize available literature, as well as identify any gaps in evidence. There is no gold standard process on how to perform a literature review so, the PRISMA checklist for systematic reviews was used [38-39].

### Search method

Articles were retrieved via an online search. This consisted of a three-stage process. The first stage included a broad online search from the following four databases: PubMed, Cochrane database, Google scholar and Elsevier. Search words used in combination in the first stage included: (driving, dementia), (driving behavior, dementia), (dementia, driving assessment), (driving, dementia, neurophysiological testing), (driving, dementia, on-road tests), (dementia, driving capacity), (dementia, fitness to drive), (driving, Alzheimer’s), (frontotemporal dementia, driving), (lewy body dementia, driving), (vascular dementia, driving) and (dementia severity, driving).

The second stage involved an incremental search by reviewing the reference lists of related literature reviews, systematic reviews and meta-analysis to identify relevant articles that were missed in the primary electronic database search.

The third stage involved the application of inclusion and exclusion criteria described in Table 5. The titles were screened initially and those titles on driving and dementia, driving and cognitive impairment or driving in old age were selected. The abstract of each selected article was screened. Finally, articles whose abstracts met inclusion criteria but not exclusion criteria, underwent a full article review.

**Table 5:** Inclusion and exclusion criteria for articles in literature review.

Inclusion Criteria	Exclusion Criteria
Primary studies published in peer-reviewed journals. Cohort studies, case-control studies, cross-sectional studies, systematic reviews with meta-analysis and mixed-method studies. Studies analysing driving behaviours in different stages of dementia. Studies analysing driving behaviours in different types of dementia including Alzheimer’s disease, frontotemporal dementia, dementia with Lewy body and/or vascular dementia. Studies on driving capacity assessments in patients suffering from different stages of dementia. Studies on driving capacity assessments in patients suffering from different types of dementia including Alzheimer’s disease, frontotemporal dementia, dementia with Lewy body and/or vascular dementia. Research published between 1st January 2000 and 1st January 2021	Case studies, letters, editorials, comments, case reports, conference abstracts, guidelines, book chapters, literature reviews, systematic reviews with narrative synthesis, unpublished studies, and grey literature. Studies that do not specify the diagnostic criteria used for dementia diagnosis. Studies in which the population includes patients with other neurological or medical conditions in addition to dementia and dementia patients are not isolated in the data analysis. Studies in which the type of dementia is not specified. Studies in which the population includes patients with non-dementia related cognitive impairment, or dementia that is not secondary to Alzheimer’s disease, Frontotemporal dementia, Lewy Body Dementia, or vascular dementia, unless data analysis of the separate groups is carried out. Articles on driving in dementia that do not concern driving behaviour and/or assessment. Articles that are not published in the English language. Research published before 1st January 2000 or after 1st January 2021.

### Quality appraisal

Critical appraisal of each article was performed. The Newcastle-Ottawa Quality Assessment Scale (NOS) and the Cochrane Risk of Bias (ROB) assessment tools were used for observational studies, while the AMSTAR-2 tool was used for quality appraisal of systematic reviews with meta-analysis.

The NOS scale is divided in three components as described by Wells GA, Shea B, O'Connell D et al. (Available online from www.ohri.ca/programs/clinical_epidemiology/oxford.asp). The first component describes the selection of participants, case definition and group allocation (maximum score of 4). The second component describes the comparability and control of confounding factors (maximum score of 2). The final component refers to the exposure in case control studies or the outcome in cohort studies, with particular focus on the mode of measurement used (maximum score of 3). The scores of each individual component and the total score were recorded for each study. Studies with global NOS score of 0-3 were rated as low quality, those with an NOS score of 4-6 were rated as medium quality and those with an NOS score of 7-9 were considered as good quality studies.

Several Risk of Bias Assessment tools are available. Two validated and commonly used tools for risk of bias assessment in non-randomized studies include the RoBANS and ROBINS-I [40-41]. The risk of each type of bias was graded as high, low, or uncertain if there is insufficient information. The following types of bias were assessed: Selection Bias, Confounding Bias, Misclassification Bias, Performance Bias, Detection Bias, Attrition Bias, and Reporting Bias [40-41].

The AMSTAR-2 tool consists of sixteen items addressed to assess the validity and reliability of results [42]. Flaws in items 2, 4, 7, 9, 11, 13 and 15 are considered as critical weaknesses. Flaws in items 1, 3, 5, 6, 8, 10, 12, 14 and 16 are considered as non-critical weaknesses. The quality of meta-analysis was ranked as follows:

- High quality: No or one non-critical weakness identified in the systematic review.
- Moderate quality: More than one non-critical weakness identified.
- Low quality: One critical weakness present with or without non-critical weaknesses.
- Critically low quality: More than one critical flaw present with or without non-critical weaknesses.

The level of evidence of each study was assessed using the updated version of the Oxford Centre for Evidence Based Medicine (CEBM) Criteria (Available online from www.cebm.ox.ac.uk/resources/levels-of-evidence/oxford-centre-for-evidence-based-medicine-levels-of-evidence-march-2009). These are described as follows:

Level 1a- Systematic review of RCTs or prospective cohort studies, with homogeneity.
Level 1b - Good quality prospective cohort studies.
Level 1c - All or none case series.
Level 2a - Systematic review of retrospective cohort studies or RCTs with untreated control groups, with homogeneity.
Level 2b - Good quality retrospective cohort studies or RCTs with untreated control groups.
Level 2c - Audits, Outcome Research or Ecological studies.
Level 3a - Systematic review of case-control studies, with homogeneity.
Level 3b - Individual good quality case-control studies.
Level 4 - Case series and poor-quality cohort and case control.
Level 5 - Expert opinion.

The level of evidence was used to grade recommendations. It is described as follows:

Grade A = Consistent Level 1 studies
Grade B = Consistent Level 2 or 3 studies
Grade C = Level 4 studies
Grade D = Level 5 studies or inconsistent and inconclusive studies from all levels.

### Data extraction and analysis

Data was extracted from the full texts to identify themes, comparisons, conflicting views, and gaps in evidence. Information was divided into four main themes: (i) driving behaviors in dementia; (ii) driving assessment in dementia; (iii) driving in different subtypes of dementia; and (iv) driving in different severities of dementia. Data was organized in table forms using an Excel sheet. The following information was extracted:

- Author and year of publication.
- Study aims and objectives.
- Population description
- Diagnostic criteria used.
- Measures/assessments used
- Results and conclusions.

## RESULTS

### 4.1 Search Outcomes

**Figure 1.**
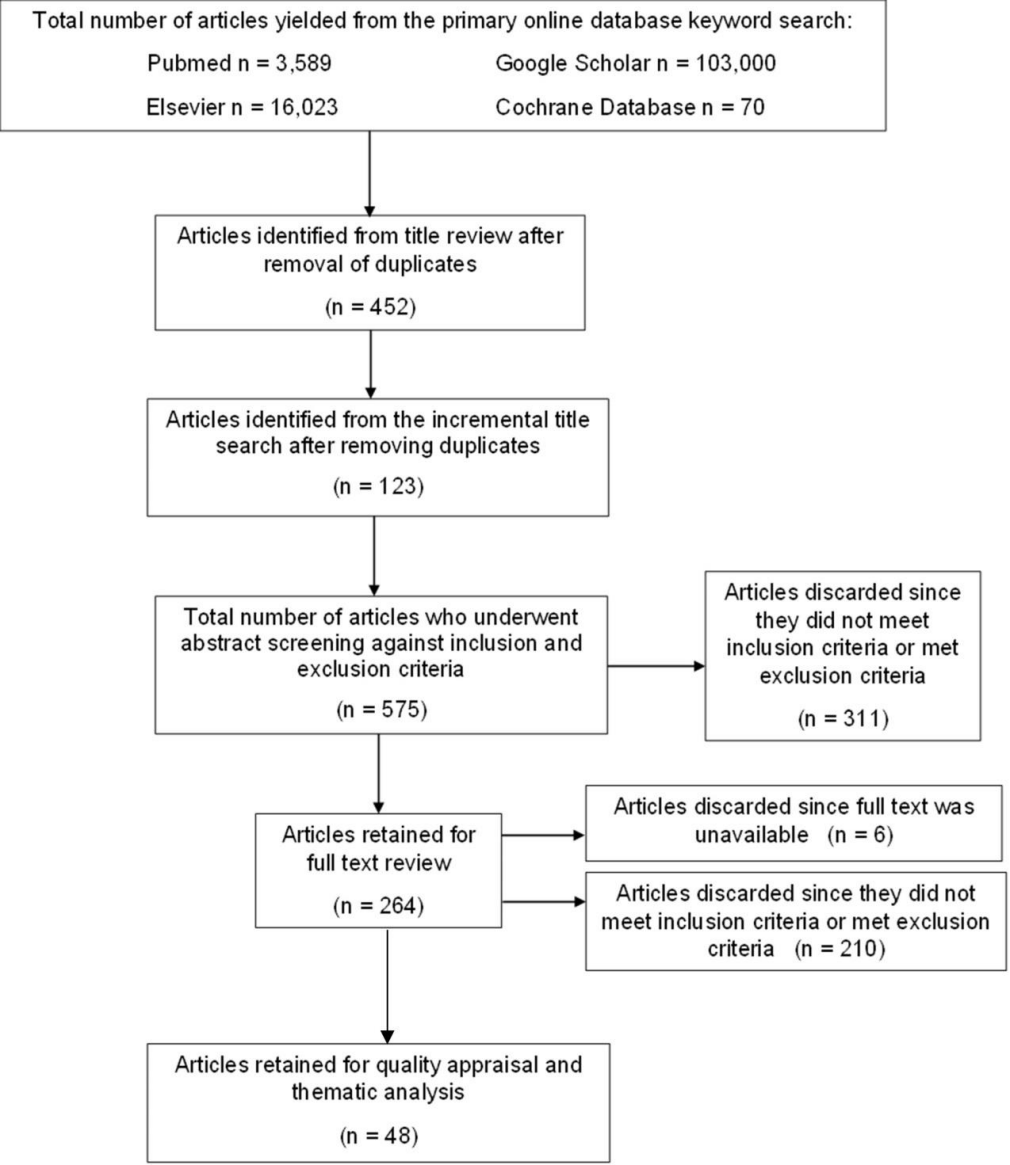
**Flow Diagram of Literature Search**. *N= number of articles*

Figure 1 illustrates a summary of the literature search. The full text of six journal articles was not found through the online search. Communications with the authors were made but no response was received, so these were not included in the final analysis. A list of the excluded articles can be found in Appendix A.

A total of forty-eight articles were included in the narrative review. These included forty-four primary observational studies [15, 43-85] and four systematic reviews with meta-analysis [86-89]. Twenty-five out of the forty-four primary observational studies were included in either of the four meta-analyses. Consequently, results from primary observational studies and meta-analysis were analyzed separately. None of the articles were discarded based on quality assessment since this is a subjective process with no gold standard quality assessment tool [90-91].

### 4.2 Study Description

Fourteen articles concerned only driving behavior in dementia (13 primary observational studies and one meta-analysis), nineteen studies examined only driving assessment in dementia (16 primary studies and three meta-analysis), and fifteen observational studies examined both driving behaviors and assessments. Twenty-six primary studies used a standardized on-road driving assessments, seven used naturalistic driving assessment, twelve used stimulated driving, fifteen used informant/patient/physician reporting as a measure of driving assessment, and thirty-four used various in-office cognitive and/or visual assessment tools. Certain studies used more than one driving assessment tool. The meta-analysis concerning driving behaviors used on-road assessment only [87]. One meta-analysis compared neurophysiological testing with on-road assessment only [89]. The other two meta-analyses compared multiple types of driving assessment methods [86, 88]. A summary of the articles can be found in the supplementary Tables 2, 3 and 4).

The primary studies combined included a total population of 1967 participants with dementi. Population across individual studies ranged from 6 to 139, with an average of 44.7 participants. The majority of participants had AD dementia (n = 1678), followed by FTD (n = 97), VaD (n = 32), DLB (n = 31), and Mixed Dementia, namely VaD and AD (n = 10). One article (n = 119) did not specify the number of patients according to subtypes [43]. Different studies used different methods for dementia severity assessment. The CDR scale was the most common (26 observational studies), followed by the MMSE (9 observational studies) and the GDS (2 observational studies). Nine observational studies did not use or describe any assessment tools for severity rating. Dementia severity was either mild or mild in thirty-two observational studies, mild to moderate in seven observational studies and five observational studies did not describe the severity of dementia. The total dementia population in meta-analysis ranged from 240 to 2037 participants. Two meta-analysis included participants with AD dementia only. Two meta-analysis included participants with dementia of any etiology and severity.

### 4.3 Quality Analysis

Four observational studies and two meta-analyses were of low quality, thirty observational studies and one meta-analysis were of medium quality and ten observational studies, and one meta-analysis was of high quality. A more detailed breakdown of the quality analysis can be found in the supplementary data (Tables 5 and 6).

The most common type of high-risk bias in observational studies was selection bias. In fact, most studies recruited participants from memory clinics. This might not be representative of the whole dementia community, since some participants might not have access to such clinics leading to selection bias. Moreover, patients who do not attend clinics might do so in view of reduced social support, non-compliance with appointments or treatment and/or lack of insight into their impairment. These factors might increase their driving risk and also make them less likely to consent and participate in such studies. Moreover, driving can be affected by various medication and other medical conditions. These might act as confounders (Available online from www.healthknowledge.org.uk/public-health-textbook/research-methods/1aepidemiology/biases). However, these were described in detail in most studies and formed part of the exclusion criteria. On the other hand, multi-morbidity and polypharmacy are extremely common in the geriatric population; therefore, the selection of healthier participants may not be representative of the whole population. In addition, the average study population was 44.7 with some studies including less than 10 participants. It is doubtful how representative this sample was of the general population. This is especially the case for participants with non-AD dementia. In fact, the effect size (ES) was not calculated in all studies, and it is difficult to determine the required sample size without the ES [92].

On the other hand, the most common type of low-risk bias was misclassification bias. In fact, the description of diagnostic criteria to define dementia was part of the inclusion criteria. This was done in order to reduce misclassification bias since there could be various causes of cognitive impairment other than dementia. This is also a form of information bias ^[93]^. Other forms of information bias analyzed, included observer or detection bias. These types of bias can be reduced by blinding [93]. However, blinding of the diagnosis is difficult to achieve when information is obtained from the participant themselves or their caregiver. The latter can also lead to recall and reporting bias [192].

The meta-analysis included in this review calculated the effect size and heterogeneity which varied from low to high. This correlates with the great variability in search method, population characteristics, assessment methods and outcome measures used. This reduced the validity and reliability of results. In addition, three out of the four meta-analyses analyzed risk of bias quantitatively. A low risk of publication bias was detected in all three.

### 4.4 Driving Behaviors in Dementia

One meta-analysis [87] and twenty-eight primary observational studies [43-46, 48-60, 63, 65-74] explored driving behaviors in dementia. Multiple assessments were used to identify driving behaviors (Supplementary data Table 2).

#### 4.4.1 Comparison between dementia and cognitively intact individuals

Twenty-two primary studies and one meta-analysis [87] compared driving behaviors of dementia drivers with age matched cognitively normal drivers. In all studies, dementia drivers had a higher driving test failure rate and overall higher error scores than cognitively intact drivers. They were also more likely to commit serious errors when compared to cognitively intact drivers [49, 65, 74]. Drivers with dementia were found to drive fewer miles per week when compared to healthy controls. However, driving frequency was not associated with driving performance [72].

**Table 6:** Driving Behaviors in Dementia

Driving behaviour measure	Number of studies which assessed the behaviour	Number of studies which identified a statistically higher incidence of each error in dementia drivers.
**Landmark/sign identification**	9	8 (88.9%)
**Adequate response to traffic signs and lights**	13	8 (61.5%)
**Lane Maintenance**	13	11 (84.6%)
**Lane Changing ability**	8	6 (75%)
**Route Learning and Following**	7	7 (100%)
**Speed control**	11	10 (90.9%)
**Turns or intersections**	14	12 (85.7%)
**Response to distractions**	3	3 (100%)
**Appropriate reaction to traffic stimuli**	11	11 (100%)
**Reaction Time**	7	7 (100%)
**Following too close**	6	5 (83.3%)
**Backing up**	3	3 (100%)
**Parking**	7	3 (42.9%)
**Sudden Braking**	9	4 (44.4%)
**MVC**	11	6 (54.5%)

Different studies assessed several types of behaviors and errors. The WURT, RIRT, TRIP and CDAS were the most commonly used scores which are described in Appendix B. Table 6 illustrates the type and frequency of the most common driving behaviors and errors in drivers with dementia. The behaviors that were constantly and significantly more common in dementia drivers, included inappropriate response to traffic stimuli, followed by poor speed control, followed by poor lane maintenance and difficulty with maneuvering turns or intersections. There were inconsistent results with regards to response to traffic lights/signs and MVCs. Other behaviors/ errors with study consistency more than 75%, included reduced reaction time, difficulty in route learning and following, poor response to distractions, difficulty backing up, impaired landmark/sign identification, following too close to the vehicle in front of them and difficulty in lane changing. Two studies assessed the participant’s perception of their driving and cognitive ability and compared it with standardized testing. Both studies identified a statistically significant reduced awareness of self-driving ability in patients with dementia as opposed to cognitively intact older adults [65,72].

#### 4.4.2 Comparison between different stages of dementia

Seven primary studies and one meta-analysis assessed driving behaviors in different stages of dementia. Three studies used the MMSE to quantify stages of dementia [56, 58, 60], six studies used the CDR score [51-52,56,60,63,87], and one study used other scores [50-52]. Results varied according to the measurement used. For instance, in all three studies using the MMSE, increased dementia severity quantified by a lower score, was not significantly correlated with a worse driving performance. On the other hand, increased dementia severity quantified by higher CDR levels was consistently related with worse driving performance.

**Figure 2.**
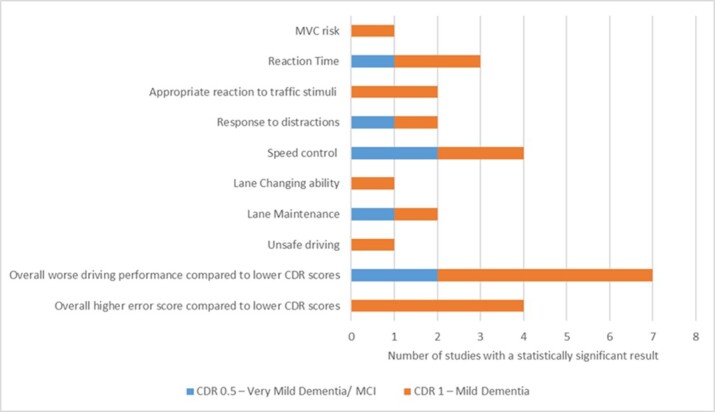
**Driving Behaviors according to Dementia Severity.** This graph shows a comparison of the number of studies in the literature search that reported the individual driving behaviors in very mild (CDR 0.5) and mild (CDR 1) dementia severity. *CDR = Clinical Dementia Rating scale.

Most studies assessed dementia participants with CDR scores of 0.5 and 1. There were inconsistent results when comparing participants with CDR scores of 0.5 to healthy controls (CDR 0). On the other hand, participants with a CDR score of ≥ 1 performed consistently worse and made consistently more safety errors when compared to both CDR 0.5 and cognitively normal participants. Three studies included dementia participants with moderate severity (CDR 2) [51, 60, 63]. All these patients made overall more driving errors, had a worse performance on standardized tests and were unsafe drivers. Figure 2 illustrates a graph comparing behavior frequency in very mild (CDR 0.5) and mild (CDR 1) dementia. However, worse performance and increased safety errors do not necessarily equate to unsafe driving. In fact, five out of six studies using CDR measures compared accident probability or pass/fail results amongst dementia severities. The studies which included moderate severity (CDR 2) identified a high rate of unsafe driving in this subgroup of patients.

#### 4.4.3 Comparison between different types of dementia

Only five observational studies included dementia subtypes other than AD [45, 50, 54, 58, 59]. One meta-analysis also included studies with different dementia subtypes, but results were not analyzed for individual etiologies [87]. Table 7 illustrates the frequency of articles which included the individual dementia etiology and the different driving behaviors identified per etiology.

### 4.5 Driving Assessments in Dementia

Thirty-two observational studies [15, 44, 46-50, 54, 57-59, 61-62, 64, 67-73, 75-85] and three meta-analysis [86, 88-89] investigated driving assessment in dementia. Table 8 illustrates the main types of driving assessments used in dementia as identified by this literature search [17-18, 36, 48, 61, 77, 94-96]. There was great variability between assessments used (Supplementary data Table 3). On-road and neuropsychological assessments were the most common tests used. The relationship between different driving assessments will be explored.

#### 4.5.1 On-Road driving assessments: Comparison between Naturalistic Driving and Standardized Road Assessment

Three primary studies compared nat, XXXllistic driving with standardized road assessments [47-48, 64]. All three used RIRT and CDAS scales as driving outcome measures. All three studies concluded that standardized on-road testing is an accurate mode of driving assessment in dementia. The reason being that it is reflective of naturalistic driving behavior when considering global performance (Level 1b, 3b). On the other hand, results vary when considering individual behaviors. In fact, road tests that stress proper lane keeping and response to traffic have standardized weighting for each item and are held in the patient’s environment, may be more representative of naturalistic driving ability. Moreover, automated computerized analysis of naturalistic driving is an accurate and practical method to monitor driving behavior in drivers with AD dementia (Level 1b).

**Table 7:** Driving Behaviors according to Dementia Subtype.

Dementia subtype	AD	VaD	Mixed (AD + VaD)	FTD	LBD
**Number of studies which included the dementia subtype**	27 [43-50, 52-74]	3 [54, 58, 87]	1 [54]	6 [50, 45, 54, 58-59, 87]	3 [50, 54, 87]
**Comparison groups if present**	Healthy controls, Semantic Dementia/FTD, VaD, Mixed Dementia, DLB	Healthy Controls, AD, FTD, Mixed Dementia, LBD	Healthy Controls, AD, FTD, VaD, LBD	Healthy controls AD, VaD, Mixed Dementia, LBD	Healthy Controls, AD, FTD, VaD, Mixed Dementia
**Driving Behaviours specific to dementia subtype**	Route Learning and Following problems, Difficulty at Turns or intersections, Poor Response to distractions, Reduced Reaction Time, Following too close.	Insufficient observation at intersections when turning right, Poor perception and judgement during overtaking, Inadequate communication with other car drivers, Manoeuvring intersections, Accelerating on the merging lane.	Insufficient observation when turning right and left, or in curves; insufficient anticipatory scanning for changes in traffic situations, Manoeuvring intersections, Difficulty with lane maintenance and changing.	Poor lane choice at roundabouts, Insufficient communication with other car drivers, cyclists and pedestrians Insufficient observation when turning right and left, or in curves; insufficient anticipatory scanning for changes in traffic situations, Manoeuvring intersections, Difficulty with lane maintenance and changing	Lane positioning and observation of the blind spot, Unsteadiness of steering, Insufficient observation at intersections, Poor perception and judgement during overtaking.
**Driving Behaviours common to all subtypes**	Lane maintenance errors, Speed control, No observation of the blind spot, Unsafe lane changing, Unsafe merging with traffic, Poor traffic sign recognition and reaction Inappropriate reaction to traffic stimuli.				

#### 4.5.2 Comparison between Simulated Driving and On-Road Driving Assessments

Three primary observational studies compared simulated driving with on-road driving assessment [15, 54, 84]. All three studies used TRIP scoring system as an outcome measure for the on-road assessment and the following outcome measures for driving simulator assessment: minimum speed when approaching an intersection with traffic lights, number of collisions, the deceleration of the rear car after merging and time headway directly after merging. Two studies explored this comparison in participants with dementia of the AD subtype only and one study included participants with non-AD dementia only. In both studies with AD participants, a significant correlation was found between on-road and simulated driving assessments. The study conducted by Piersma D, Fuermaier ABM, De Waard D et al. (2016), calculated a predictive value of 75% for simulated driving assessment when compared to on road pass/fail scores. On the other hand, driving simulator assessment was not predictive of driving competence in participants with non-AD dementia when compared to road-testing.

**Table 8:** Types of Driving Assessments used in Dementia.

	On Road Assessments	Driving Simulator	Clinical Interviews	Neuropsychological tests
**Description**	The participant’s driving ability is assessed by driving on the road in a vehicle.	An instrument which includes the inside structures of a vehicle but it is attached to a screen which simulates driving scenarios.	Participant self-rating, caregiver rating and clinician rating. Multiple measures can be included.	Used to assess various cognitive domains and global cognition.
**Advantages**	Closest association with naturalistic driving and considered the gold standard assessment.	Safe; Scenarios are easier to control in order to test multiple environments	Safe, Part of routine visits, Caregivers spend most time with the patients and can see them in their natural environment	Multiple tests available Standardised score In-office assessment
**Disadvantages**	Time consuming, Trained instructors needed to perform the assessment, Expensive, Cannot control assessment environment.	No standardization in type of simulator, scoring systems and scenarios used; Limited availability	Subjective opinion, Recall bias,	Time consuming Training in performing tests is needed.
**Factors that can affect driving performance**	Test-related anxiety, Instructor bias, Type of scoring system used, Use of unfamiliar vehicles or environments, Driving scenarios present during the test might not be representative of all driving behaviors or performance in risky situations.	Unfamiliar to participants so they need to learn a new skill which might be difficult for dementia patients. Simulator Sickness	Reduced awareness of their driving ability, Caregivers may depend on the patient for transportation, Fears of taking driving license, Lack of confidence or knowledge regarding dementia and driving assessment, Fear of breaking doctor-patient relationship.	Too many tests available can lead to confusion with regards to which one to choose, Too little evidence on the best cut-off scores to use. Lack of training on how to perform test can result in wrong results. Sensory impairments can affect results.
Accuracy and predictive values	Accuracy: RIRT 72.9% vs CDAS 60.3% vs Mockingbird 91.7% Sensitivity: 92.7% Mockingbird vs 74.4% RIRT vs 50% CDAS Specificity: CDAS 87.5% vs 68.8% RIRT vs 33.3% Mockingbird	Compared with on-road assessment		
		**Accuracy in predicting fitness to drive in AD: 85.6%**	Accuracy in predicting fitness to drive in AD: 94.6%	Accuracy in predicting fitness to drive in AD: 79.6%

#### 4.5.3 Comparison between Caregiver/Clinician/ Participant Rating of driving ability and On-road Driving Assessment.

Seven observational studies compared caregiver/ clinician/participant rating with on-road driving assessment [15, 44, 46-47, 61, 72, 84]. Table 9 compares the different types of interview ratings in individual studies. In general, clinician rating was more accurate than caregiver rating (Grade B) and caregiver rating was more accurate than participant self-rating (Grade D).

#### 4.4.4 Comparison between Caregiver/Clinician/ Participant Rating of driving ability and Simulated Driving Assessment.

Three studies compared caregiver/participant self-rating and simulated driving assessments [15, 44, 84]. In all three studies, both assessments were compared individually with on-road outcome measures rather than between each other. In general, simulated driving had a higher predictive accuracy when compared to clinical rating in AD dementia (Grade D). Results varied in non-AD dementia (Grade D).

**Table 9:** Correlation between clinical rating of driving ability compared to on-road driving assessment in drivers with dementia.

Article reference	Scoring system	Participant Self-rating	Caregiver Rating	Clinician Rating
**Bixby K, Davis JD, Ott BR (2015)**	RIRT CDAS	Not assessed	No statistically significant correlation	Significantly poorly correlated with test score errors in on road testing but not with global scores or naturalistic driving.
**Brown LB, Ott BR, Papandonatos GD et.al. (2005)**	WURT	PPV 46.7% * NPV 100% Specificity 10.7% Sensitivity 100% Correctly classified 53.2%	PPV 60% NPV 73.3% Specificity 47.8% Sensitivity 81.8% Correctly Classified 64.4%	PPV 64.5% NPV 89.5% Specificity 60.7% Sensitivity 90.9% Correctly Classified 74%
**Ott BR, Anthony D, Papandonatos GD et.al. (2005)**	WURT	Not assessed	Not assessed	Correct classification ranged from 62% to 79% between different clinicians. Great variability between PPV, NPV, Specificity and Sensitivity between clinicians.
**Piersma D, Fuermaier ABM, De Waard D et.al. (2016)**	TRIP	When considered together, clinical ratings showed an overall accuracy of 83.5%.	Not Assessed
		Predictive of fitness to drive at a statistical significance even after binary logistic regression analysis	Did not reach statistical significance after binary logistic regression analysis.	
**Piersma D, Fuermaier ABM, De Waard D et.al. (2018)**	TRIP	NO correlation between self-rating scores and on road driving scores in participants with non-AD dementia	Not assessed	Not assessed
**Wild K and Cottrell V (2003)**	Driving Safety Evalua-tion score	Dementia participants overestimated their driving ability. Self-rating was better than on-road instructor rating in 7 out of 10 items.	Caregiver rating was more accurate than participant self-rating. Similar scores between caregiver and on road rating in 8 out of 10 items (managing intersections and responding to road signs)	Not Assessed
**Crivelli L, Russo MJ, Farez MF, Bonetto M, Prado C et.al. (2019)**	Standr-dised Road Test	Did not analyse the statistical difference between caregiver/ participant self-rating and on-road driving assessment but rather the difference in scores between dementia and non-dementia participants. There was no statistically significant difference in both caregiver rating and participant self-rating of driving ability despite there being a statistically significant difference in error frequency on the on-road assessment, making the correlation between clinical rating and on-road testing unlikely.		

* PPV = positive predictive value, NPV = negative predictive value

#### 4.5.4 Comparison between In-Office Neuropsychological Tests and On-road Driving Assessment.

Seventeen primary observational studies compared in-office neuropsychological tests with on-road driving assessment outcomes to assess predictive accuracy of fitness to drive in dementia. Table 10 gives a list of the tests included in the studies and the number of studies that found a statistically significant correlation between individual neurophysiological tests and other driving assessments. The most common tests used were the TMT-B, MMSE and TMT-A, followed by ROCFT, UFOV, WAIS Block Design, BVRT, COWA, AVLT, CS, SFM, CDR, JLO, Maze Tests, NVA, FVA, Verbal fluency, COGSTAT.

The Trial Making Test (TMT) B was included in sixteen studies and compared to on-road assessment in twelve studies. None of these studies included score cut-off points. 33.3% of studies found no statistically significant correlation between TMT-B scores and on road driving outcomes. Two of these studies used ARGOS instrumented vehicles with driving errors as outcome measures [49, 75]. The other two studies used Naturalistic driving using both error scores and safety risks as outcome measures [46, 48]. On the other hand, 66.7% of the studies found a statistically significant correlation between TMT-B scores and on-road assessment measures. All of these studies used standardized road tests and included drivers with dementia of AD subtype. The following measures were included:

- Driving error scores: Error measures that were significantly correlated with TMT-B scores included general behavior, traffic sign/landmark identification and compliance, ability to follow instructions, WURT scores, route following, RIRT driving awareness [44, 62, 67-768, 71].
- Driving Safety: TMT-B scores were strong predictors of driving safety in drivers with AD dementia [15, 57].

**Table 10.** Relationship between individual neuropsychological tests and other driving assessment tools.

Neuropsychological Test	On-Road Assessment (* n=19)	Simulated Driving (n= 6)	Caregiver Rating (n= 1)	Participant Self Rating (n= 1)	Collisions/ MVC history (n= 12)
**Trial Making Test B** **TMT-B (+ N=16)**	ˠ 8/12 [15, 44, 57, 62, 68, 67, 71, 88]	2/5 [44, 69]	0/1	0/0	1/2 [85]
**Mini Mental State Examination MMSE (N=15)**	7/15 [15, 44, 48, 54, 79, 80, 88]	1/5 [44]	0/1	0/2	0/5
**Trial Making Test A** **TMT-A (N=11)**	10/11 [15, 46, 49, 44, 57, 62, 68, 71, 75, 88]	1/1 [44]	0/1	0/0	0/1
**Rey Osterreith Complex Figure Test ROCFT (N= 9)**	5/6 [69, 57, 67-68, 88]	2/2 [85, 69]	0/1	0/2	1/2 [85]
**Usual Field of View** **UFOV (N=9)**	4/5 [49, 67-68, 88]	3/4 [69-70, 73]	0/0	0/0	½ [70]
**Wechsler Adult Intelligence Scale-III Block Design Test** **WAIS BD (N=8)**	3/4 [67-68,75]	1/4 [85]	0/0	0/0	0/1
**Benton Visual Retention Test BVRT (N=8)**	4/5 [49, 67-68, 88]	1/3 [69]	0/0	0/0	0/1
**Controlled Oral Word Association Test COWA (N=7)**	2/3 [67-68]	1/3 [85]	0/1	0/0	1/2 [85]
**Auditory Verbal Learning Test AVLT (N=6)**	2/4 [68-69]	0/2	0/0	0/0	0/1
**Contrast Sensitivity** **CS (N=6)**	3/4 [67-68, 88]	1/2 [69]	0/0	0/0	0/1
**Structure from Motion SFM (N=5)**	0/4	0/1	0/0	0/0	0/0
**Clinical Deterioration Rating Scale CDR (N= 5)**	2/4 [15, 54]	0/1	0/0	0/1	0/0
**Judgment of Line Orientation JLO (N=5)**	2/3 [67-68]	1/2 [69]	0/0	0/0	0/1
**Maze Tests (N= 5)**	4/5 [15, 48, 62, 88]	0/0	1/1 [82]	0/0	0/0
**Near Visual Acquity NVA (N=5)**	2/3 [67-68]	0/2	0/0	0/0	0/1
**Far Visual Acquity FVA (N=5)**	2/3 [67-68]	0/2	0/0	0/0	0/1
**Computerised cognitive assessment COGSTAT (N=5)**	2/3 [67-68]	2/2 [69, 85]	0/0	0/0	1/1 [85]
**Verbal fluency (N=4)**	2/2 [44, 88]	1/2 [59]	0/0	0/0	0/0
**Clock Drawing Test - CDT (N= 4)**	0/3	1/1 [71]	0/1	0/0	0/0
**Boston Naming Test - BNT (N=3)**	1/1 [49]	0/2	0/0	0/0	0/0
**Digit Symbol Substitution/Modalities Test DSST/DSMT (N=3)**	2/2 [49, 57]	0/1	0/0	0/0	0/0
**Hopkins Verbal Learning Test HVLT (N= 3)**	1/3 [68]	0/0	0/0	0/0	0/0
**Reaction time S2 (N=3)**	1/3 [55]	0/0	0/0	0/0	0/0
**Hazard perception test (N=3)**	1/3 [55]	0/0	0/0	0/0	0/0
**Traffic theory test (N=3)**	1/3 [55]	0/0	0/0	0/0	0/0
**Finger Tapping Test (N=3)**	1/3 [88]	0/0	0/0	0/0	0/0

* n=number of studies which included the individual driving assessment

+ N=number of studies which included the individual neuropsychological test.

ˠ Number of studies which found a statistically significant correlation / number of studies which investigated the correlation between respective neuropsychological test and other driving assessment tools.

The Mini Mental State Examination (MMSE) was included in fifteen studies all of which used on-road driving assessments. Only 46.7% of these found a statistically significant association between MMSE scores and road driving outcomes. Only one study included cut-off scores, with a cut-off score of 19 yielding a sensitivity of 42% and a specificity of 89% in predicting unsafe driving in dementia [79]. The following outcome measures were included:

- Driving errors: Error outcomes associated with MMSE scores included general behavior, ability to follow instructions and Mockingbird error scores [44, 48]. Conflicting results were found regarding the association between MMSE and traffic sign/landmark identification and compliance [44, 67].
- Driving safety (safe/unsafe outcomes): Two studies found a significant correlation between lower MMSE scores and unsafe driving outcomes in drivers with AD dementia [18, 89]. However, this correlation was not present in another study conducted by Piersma D, Fuermaier ABM, De Waard D et al. which included participants with non-AD dementia [84].
- No correlation was found between MMSE scores and RIRT, CDAS, visual discrimination stimuli, lane observance errors and route following tasks [48-49, 68, 71,83].

Eleven studies compared Trial Making Test A (TMT-A) scores with on road outcome measures. 90.9% of theses found a statistically significant correlation between the two. Correlation ranged from moderate to high. The following correlations were present:

- Driving errors: Error outcomes associated with TMT-A scores included total error scores, general behaviour, ability to follow instructions, speeding events, hard acceleration events, number of safety errors, WURT test scores, RIRT total error score and driving awareness scores [44, 46, 49, 62, 71, 75].
- Driving safety (safe/unsafe outcomes): Two studies found a significant correlation between TMT-A scores and unsafe driving outcomes in drivers with AD dementia [15, 57].
- No correlation was found between TMT-A and Mockingbird scores on computerized analysis of naturalistic driving [48].

Table 10 shows multiple other neuropsychological tests. Other tests were compared with on road assessments in less than ten studies, the maximum being six. Tests which were analyzed in two or less studies were not included in the table and include the following: MDR, NAB, VOSP, MOCA, TEA, Facial Recognition Test, Ideomotor praxis, Verbal Paired Associates Test, Logic Memory - Immediate and Delayed, WMS, FMS, Corsi blocks, PPT, Luria's motor sequence, Stroop test, Token Test, Easy Picture Naming, reading and word matching, LACLS, CPT, Money Road Map Test, ATAVT - VTS, Isaacs’s Set Test, Zazzo’s Cancellation Test, NPI-Q, FAQ, FDS.

The following tests had a consistent correlation with on-road tests across studies:

- ROCFT: Correlation found in 83.3% of studies, specifically with total driving errors, lane observance errors, driving safety/ fitness to drive, route following tasks, at fault safety errors, landmark and traffic sign identification [49, 57], 67-68, 75].
- UFOV: Correlation found in 80% of studies, specifically with landmark and traffic sign identification, route following tasks, incorrect turns, lane observance errors [49, 67-68].
- BVRT: Correlation found in 80% of studies, specifically with total number of safety errors, landmark and traffic sign identification and route following tasks [49, 67-68].
- Maze Tests: Correlation found in 80% of studies. There was a modest correlation between Mocking-bird scores and time to perform mazes and number of errors on mazes [48]. Two studies found a significant correlation between Maze test and driving safety [15, 62].
- DSST/DSMT: A statistically significant correlation with on road driving outcome was found in two out of two studies which assessed this relationship. One test also identified a cut-off score of 25 as having the highest sensitivity (92.2%) with a specificity of 75% [44, 80].

Even though they were only included in one or three studies, it is worth describing the findings related to the Clock Drawing Test (CDT) and the Montreal Cognitive Assessment (MoCA) in view of their widespread use in clinical practice. None of the three studies exploring the relationship between CDT and on-road tests found a significant correlation between the two, even after comparing different scoring systems [48, 71, 81]. Only one study explored the correlation between the MoCA test and on road driving assessment. One study calculated the cut-off points and their sensitivity and specificity at predicting unfitness to drive [79]. The MoCA test with a cut-off score of 16 had a sensitivity of 50% and a specificity of 89% which was similar to the MMSE score with a cut-off of 19. In fact, this study concluded that the Cognitive Performance Test (CPT) was the most accurate at predicting unfitness to drive (cut-off score 4.7, sensitivity 89% and specificity 75%). However, these were calculated for the whole study population rather than the dementia participants alone.

Hird MA, Egeto P, Fischer CE et al. (2016) conducted a meta-analysis aiming to compare several neuropsychological tests to various driving outcome measures. The majority of studies included road driving assessments [88]. The tests and number of included studies are described in Table 11. Maze Test, TMT-A, TMT-B, verbal fluency, UFOV, SFM, CS, Finger Tapping, BVRT, ROCF-Copy and MMSE significantly predicted driving outcome with Maze Test, TMT-A and TMT-B being the best predictors. These results correlate with the above analysis.

**Table 11.** Association between dementia etiology and driving assessment.

	On-road assessment (n=33)	Simulated Driving (* n=12)	Neurophysiological tests (n=34)	Clinical interviews (n=15)
**AD** **(+ N=33)**	ˠ 8/8 [15, 44, 47-48, 64, 71, 76, 83]	3/4 [15, 44, 54]	20/22 [15, 44, 46, 48-49, 54, 57, 59, 62, 67-71, 73, 75, 77, 80, 81-90, 85]	5/7 [15, 44, 54, 61, 72, 76-77]
**FTD** **(N=5)**	1/1 [15]	0/1 [15]	2/3 [15, 50, 59]	0/1 [15]
**VaD** **(N=3)**	1/1 [15]	0/1 [15]	1/1 [15]	0/1 [15]
**LBD** **(N=2)**	1/1 [15]	0/1 [15]	1/1 [15]	0/1 [15]

AD = Alzheimer’s Dementia, FTD = Frontotemporal Dementia,

VaD = Vascular Dementia, LBD = Lewy Body Dementia.

* n=number of studies which included the individual driving assessment

+ N=number of studies which included the specific dementia subtype.

ˠ Number of studies which found a statistically significant correlation / number of studies which investigated the predictive accuracy of the driving assessment tools in relation to the dementia subtype.

#### 4.5.6 Comparison between In-Office Neuropsychological tests and Simulated Driving Assessment.

Six studies compared neurophysiological assessment with simulated driving. The MMSE and TMT-B were the most common tests compared to driving simulators. The frequency of correlation between driving simulator outcomes and TMT-A, ROCFT and UFOV reflects the frequency of correlation with on road assessment described above. On the other hand, the correlation between TMT-B and MMSE with simulator driving assessments vary according to the outcome variable used. The following correlations were identified:

- Traffic Signal identification/reaction with MMSE, Logical Memory, DSMT, BNT, RAVLT, ROCFT, FAB, NPI-Q and FAQ [44].
- Brake Reaction time with TMT-A, TMT-B, Verbal semantic Fluency and FDS [44].
- Collisions or crash detection with Neurobehavioral Rating Scale, ROCFT, WAIS-R Block Design, TMT, motion perception, COWA, ADSTAT, COGSTAT, JLO, BVRT, FVA, CS, VOSP and UFOV [49-50,73, 85].
- Rater Score of fitness to drive with TEA and UFOV [73].

#### 4.5.7 Comparison between Caregiver/Participant Rating and In-Office Neuropsychological Assessment.

Two studies compared neuropsychological assessments with caregiver/informant rating. The study was conducted by Ott BR, Heindel WC, Whelihan WM et.al. (2003) was divided into two parts. A significant correlation between caregiver rating of driving safety and the time to complete the Proteus Maze Drawing was found in the first part. A correlation between the total score of 10 Maze tasks and CDR scores, was found in the second part of the study [62]. The other study did not compare individual neurophysiological tests with caregiver rating but rather the predictive accuracy of neurophysiological tests with caregiver rating. This study concluded that neurophysiological tests were more accurate than caregiver or participant rating at predicting fitness to drive in drivers with AD dementia (accuracy 94.6% vs. 79.6%) [15].

#### 4.5.8 Driving Assessments according to Dementia Severity

Twenty-one observational studies exploring driving assessment, used participants with dementia of very mild severity (CDR 0.5, MMSE >26, GDR ≤ 3), twenty-two used participants with dementia of mild severity (CDR 1, MMSE 21- 25, GDR 4-5), and five used participants with dementia of moderate severity (CDR 2, MMSE 10-20). Few studies explored the specific correlation between dementia severity and driving assessments. None of the studies assessed the predictive value of neurophysiological tests according to dementia severity. On-road assessment was used in three studies which yielded inconsistent results [47, 57, 88]. Simulated driving was used in two studies giving inconsistent results [50, 88]. One study compared the predictive accuracy of driving safety between physician, informant and participant rating with on-road testing. It found no significant difference between both assessments in the very mild (CDR 0.5) and mild (CDR 1.0) AD groups [78].

#### 4.5.9 Driving Assessments according to Dementia Etiology

Thirty-three out of thirty-five studies exploring driving assessments in dementia included participants with AD dementia. Of these, twenty-eight studies included participants with dementia of the AD subtype only. Only six studies included non-AD dementia [50, 68-59, 54, 79, 84]. Among these, FTD was the most common subtype followed by VaD, LBD and Mixed dementia. These concluded that the predictive accuracy of different driving assessment methods is affected by dementia subtypes (Grade B). As a result, the etiology of dementia should be considered when assessing driving competence in dementia. Table 11 describes the driving assessments that were found to have statistically significant results according to various etiologies.

#### 4.5.10 Timing of Driving Re-assessment in Dementia

Three studies explored the longitudinal relationship of driving competence in dementia [51, 58, 63]. One study included dementia drivers with AD, VaD and FTD and two studies included drivers with AD dementia. All three used a standardized road test. These concluded that a higher CDR score indicating higher disease severity, should prompt driving assessment. This is especially the case if associated with older age. A six-monthly reassessments period for those who pass the initial assessment was recommended (Grade C).

## DISCUSSION

Driving behaviors and assessments are important to help clinicians identify patients at risk of unsafe driving. Various tools were identified to help assess driving competence. However, one must remember that competence does not always relate to driving performance since the latter is influenced by multiple extrinsic and intrinsic factors [5]. Moreover, there was great variability between studies with regards to population size and type, aims, study design, driving assessments and outcome measures used. These in turn yielded variable results, especially with regards to driving assessments.

### Cognitive domains and driving

Driving involves the activation of multiple brain regions and the use of multiple cognitive domains. Cognitive impairment is the main symptom of dementia. Table 12 gives a summary of the significant associations between individual cognitive domains, driving behaviors and assessments identified in the literature search. Attention, visuospatial skills, and executive function were predictive of most driving behaviors. In addition to global cognition, these three cognitive domains were also predictive of driving safety and collision risk. On the other hand, language and social cognition were the least common predictors of driving outcomes. However, social cognition was only investigated in one study.

Three meta-analyses explored the relationship between cognitive domains and driving assessments. Rashid R et al. (2020) used only on road testing as an assessment measure and concluded that executive function, memory, attention and visuospatial skills were accurate predictors of driving performance [89]. The other two meta-analyses used on-road assessment measures, driving simulators, caregiver/participant rating, crash records and driving knowledge tests. Hird MA, Egeto P, Fischer CE et al. (2016) concluded that executive function, attention, visuospatial function, global cognition, visual memory, and vision were significant predictors of driving outcome in all assessment modalities used [88]. On the other hand, Reger MA, Welsh RK, Watson GS et al. (2004) found a correlation between visuospatial skills and all driving assessments at varying degrees, general cognition with simulator test and care-giver rating, and attention with on-road tests [86]. However, the meta-analysis by Reger MA, Welsh RK, Watson GS et al. (2004), was of poor quality and the risk of bias was not analyzed, as opposed to the meta-analysis by Hird MA, Egeto P, Fischer CE et al. (2016), which was of high quality. Morever, the meta-analysis by Hird MA, Egeto P, Fischer CE et al. (2016) did identify a degree of publication bias in all cognitive domains except global cognition, visual memory, and psychomotor cognition. Moreover, ES was high for on-road testing but low for simulated driving so results in the latter should be interpreted with caution.

**Table 12.** Association between Cognitive domains and driving behaviors and assessments.

Cognitive Domain	Driving behaviours	Driving Assessments
**Global Cognition**	Route following Landmark/ traffic sign identification Collision detection	On-road assessments Caregiver Rating (low correlation) Neurophysiological tests: MOCA, Cogstat, Sort Blessed Test, MMSE, CDR, Dementia Rating Scale, MDR, Temporal Orientation, Behavior Rating Scale, Direct Assessment of Functional Status, Full Scale IQ, IADLs, Shipley IQ Estimate, Sum of Boxes, Expanded Constructional Praxis.
**Attention**	Speed control Brake use Lane observance errors Incorrect turns Route following Collision detection Driving awareness Driving safety	On-road Assessments Neurophysiological tests: TMT-A, Digit Span, UFOV, Digit Symbol, TEA, Attention Switching, CPT, Crossing-Off, Letter Cancellation, Mattis Attention, Vigilance, WORLD spelled backwards, AMIPB Information processing, NAB total score corrected, Hazard Perception Test, Es and Fs, Measure of Inhibition (RTS3 and MTS3), Visual Orientation Two Mazes, Grooved Peg Board, Auditory Attention, Reaction Time (RT S2), Balloon Test, Letter Cancellation Test, Zazzo’s Cancellation Test.
**Memory**	Route Following Landmark/traffic sign identification Lane Observance errors Incorrect turns At-fault safety errors	On-road assessment Neurophysiological Tests: Logic Memory, Benton Visual Retention Test, Visual Reproduction, Facial Recognition Test, Associate Learning, Mattis Memory, Recognition Memory for Faces or Words, Spatial Recognition Test, Word List Learning, HVLT, AVLT, BVRT, Wechsler memory Scale (Logic Memory and Verbal Paired Association Test), Digit Span Test Backwards, ROCF-Recall.
**Executive Function**	Route Following Landmark/traffic sign identification Driving safety Brake use Collision avoidance Response to traffic	On-road assessment Driving simulator Neurophysiological tests: TMT-B, Stroop Test, Shipley Abstraction, Word Fluency, Picture Arrangement, Category Fluency and Naming, Mattis Initiation/ Perseveration, Mazes.
**Language**	Road sign naming and comprehension	Neurophysiological tests: Boston Naming Test, Information, Verbal IQ, Aphasia Battery, Comprehension, Shipley Vocabulary, Reading IQ Equivalent, Modified Token Test, COWA, Semantic Fluency Test with Isaacs’s Set Test.
**Visuospatial Skills**	Total driving error scores Lane observance errors Incorrect turns Landmark/traffic sign identification Driving safety Route following Brake use Response to traffic Speed control Driving safety Collisions	On-road Assessment Care-giver rating (low correlation) Driving simulator Neurophysiological tests: ROCF-Copy, WAIS Block Design, BVRT, UFOV, VOSP Benton Copy, Drawings, Figure Ground Test, JLO, Money Road Map Test, Visual Form Discrimination, Picture Completion, Visual Discrimination Stimuli, NAB Map Reading, CDT, Picture completion.
**Social Cognition**	Speeding Collisions	NBRS

### Driving in different dementia etiologies

Different dementia etiologies have different types of cognitive dysfunction in early disease. This might affect driving performance and assessment accuracy across subtypes differently (Refer to Tables 3 and 7). The majority of research and data available on driving in dementia concerns dementia of the AD subtype. In fact, little data is available for driving assessment in other types of dementia.

Only one study compared the driving behaviors among multiple dementia etiologies [54]. The study yielded the following results:

- Percentage fail rate per group on the on-road assessment via TRIP: 69% VaD, 66% Mixed dementia, 57.5% AD, 50% FTD, 35% DLB, 11.1% HC.
- Driving errors that were common to all subtypes: Lane maintenance errors, speed control, no observation of the blind spot, unsafe lane changing and unsafe merging with traffic, poor traffic sign recognition and reaction. In more than 50% of the individual groups, there was a general impression of poor response to traffic stimuli. These are related to visuospatial skills, executive function and attention which are impaired in most forms of dementia [15, 73].
- Driving errors are more common in specific groups: AD, mixed dementia and FTD patients made primarily tactical errors. VaD and LBD/PD participants committed both tactical and operational errors. VaD participants had the highest rate of driving errors and difficulties amongst all the groups, especially when applying rules at intersections. Both VaD and LBD patients had a degree of operational errors which might reflect the early motor symptoms associated with these conditions. Participants with FTD were noted to disregard pedestrians and cyclists on the road reflecting impairment in social cognition. In fact, FTD participants with a behavioral variant were more likely to fail the test, compared to language variant FTD.

The highest failure rate occurred in VaD patients. The study conducted by Piersma D, de Waard D, Davidse R et al. (2018), showed similar results with regards to on road failure rates (VaD 71%, FTD 58% and LDB 38%) [84]. However, the study conducted by Fitten LJ, Perryman KM, Wilkinson CJ, et al. (1995) showed a worse driving performance in AD drivers compared to VaD drivers [97]. Moreover, VaD symptomatology is highly variable since it depends on the affected brain region. For instance, multi-infarct dementia is associated with mild cognitive decline whereas subcortical VaD is associated with early marked executive dysfunction [19]. The type of VaD was not described in either study.

On the other hand, one would expect a higher degree of failure in the DLB group since it is characterized by attentional and visuospatial deficits which were associated with driving impairment [98-99]. In fact, Piersma D, de Waard D, Davidse R et al. (2018) identified an on-road test failure rate of 62% in drivers with DLB [93]. However, fluctuations in attention are also a feature of DLB so the patient’s driving performance rather than competence might fluctuate during the driving test.

As illustrated in Table 7, most behaviors overlap between different etiologies, particularly AD and FTD. In fact, failure rate was comparable between the two subtypes (AD 57% versus FTD 50%). Moreover, speed control and traffic sign recognition/naming were deficient in both etiologies. This is consistent with the fact that FTD has a language variant and AD has a non-amnesic variant with a similar cognitive profile to FTD. However, another study concluded that participants with semantic dementia scored significantly worse than AD drivers in road sign naming and comprehension, but not route learning which is dependent on memory [59]. In addition, FTD was consistently associated with an increased collision risk as opposed to AD, whose studies produced inconsistent results.

The above results suggest that etiology of dementia affects driving performance, but does it affect the predictive accuracy of driving assessments? Two studies conducted by Piersma D, Fuermaier ABM, De Waard D et al. showed the best comparison between driving assessments in AD and non-AD drivers. The initial study conducted in 2016 compared the accuracy of various assessment tools in predicting fitness to drive in participants with AD dementia with TRIP road driving measures as a point of reference [15]. This study concluded that neuropsychological assessments (94.6% accuracy) were most accurate at identifying fitness to drive in AD, followed by driving simulator tests (85.6% accuracy), and followed by participant rating (79.6% accuracy). However, the combination of these three measures provided the highest diagnostic accuracy (97.4% accuracy). A similar study was conducted in 2018 where participants with non-AD dementia (FTD, VAD, LBD) were included [84]. This study showed that only neurophysiological tests alone (AUC=0.786) were significant predictors of fitness to drive in participants with non-AD dementia. In fact, there was no statistically significant correlation between on-road TRIP scores and simulator driving scores, participant rating and the three tests combined.

This study also compared results between the different non-AD subtypes and concluded the following:

- Vascular dementia (n=14): Had the worst scores in on-road assessment, participant self-rating and neurophysiological tests when compared to other subtypes.
- Lewy Body Dementia (n=8): Had the best/safest mean scores in all predictor variables compared to other subtypes. They made more driving errors on simulated driving when compared to on-road assessment.
- Frontotemporal dementia (n=12): Had the highest self-judgment rate but were more likely than the other two groups to approach an intersection with traffic lights at high speed. They made more driving errors on simulated driving assessment compared to on-road assessment. Another study found a correlation between speeding and collision risk on simulated driving with increased agitation and aggression on the NBRS [50].

As a result, the etiology of dementia should be considered when choosing a driving assessment method since the predictive accuracy varies. For instance, neurophysiological tests rather than participant/caregiver rating could be used as office-based tests in all subtypes. On-road assessments may be needed if concern about driving fitness is identified during the clinical interview. Moreover, driving simulator assessments may also be considered for task-specific skills like collision risk in patients with FTD if this scenario is not presented during the on-road assessment.

### Driving and dementia severity

Dementia severity is associated with cognitive and functional decline. The CDR was the most commonly used tool. It also yielded the most consistent results to assess dementia severity in fact, there is consensus that drivers with moderate to severe dementia defined by a CDR of 2 or more, are unfit to drive since this is associated with marked cognitive and functional impairment [19]. However, driving fitness in mild disease (CDR 0.5-1) is more difficult to determine [100].

The impact of dementia severity on specific behaviors was analyzed in only three studies [51-52, 56]. Moreover, it is very difficult to distinguish between MCI and early AD dementia. Pavlou D, Papadimitriou E, Antoniou C et al. (2017), identified the presence of the following behaviors in both MCI and early AD when compared to healthy participants: slower reaction time (MCI = AD), longer mean headway (AD>MCI), lower mean speed (MCI=AD) and lane maintenance problems (AD>MCI) [101]. These outcomes are consistent with the results in this literature review.

However, impaired driving behaviors do not necessarily imply unsafe driving. In fact, as opposed to total errors and performance scores, results concerning pass/safe outcomes were inconsistent for CDR 0.5-1 severities. Longitudinal studies can best describe the safety outcome according to dementia severity. Two were identified in this review which assessed the driving performance of drivers with AD dementia at six monthly intervals at two and three years [51, 63]. Both studies used standardized road testing (WURT) to determine fitness to drive. Duchek JM, Carr DB, Hunt L et.al. (2003) concluded that drivers with CDR 1 dementia severity who were safe at baseline, took a significantly shorter time than non-dementia drivers to become unsafe. On the other hand, the time for the very mild AD drivers (CDR 0.5) to become unsafe fell between HC and the mild AD groups (CDR 1), but the difference was not statistically significant. Mild AD drivers (CDR 1) had the highest fail rate at baseline (41% vs 14%), but very mild AD drivers (CDR 0.5) had the highest marginal score at baseline (24% vs 17%). This indicates that monitoring is important at both stages. On the other hand, the study conducted by Ott BR, Heindel WC, Papandonatos GD et.al. (2008), did not reveal an increase in unsafe ratings at 18 months in AD participants. However, the number of marginal scores increased and the number of CDR 1 severities increased at 18 months, indicating disease progression. Moreover, 77% of AD drivers had stopped driving either due to unsafe driving or disease progression, and thus dropped out of the study. This value increased to 91.7% at 36 months. Despite this, a significant difference in median time to failure was observed between participants with CDR 0.5 and CDR 1 at baseline (605 days versus 324 days, respectively). Both studies found increasing age as a significant predictor of unsafe driving. Both studies also recommended a six-monthly driving assessment in patients with early-stage dementia. The same conclusion was reached by Lovell RK and Russell KJ (2005) who included dementia drivers with AD, VaD and FTD [58]. However, research in this area is lacking as evidenced by a systematic review who identified only three articles concerning period of follow-up assessment, one of which was explained above [102].

### On-road and non-road driving assessments in dementia

The ideal driving assessment should mimic naturalistic driving. However, patients with dementia may adopt compensatory mechanisms to help them cope with their impairment. For instance, most studies report a lower driving milage in patients with dementia compared to healthy elderly drivers. In fact, a qualitative study identified several compensatory strategies including exclusive daytime driving, driving during non-rush hours, driving in familiar places and shorter distances [103]. Some patients also reported the use of driving aids like GPS or driving accompanied. However, anything that increases cognitive demand may affect driving performance. These include any form of distractions like following commands, performing additional tasks or test-related anxiety [43-44, 52, 67-68, 72]. In fact, the study conducted by Paire-Ficout L, Marin-Lamellet C, Lafont S et al. (2016) showed a worse performance in dementia drivers when maneuvering intersections when a navigational device like a GPS was used [104]. However, there is limited literature on the use or benefit of advanced driver-assistance systems (ADAS) as compensatory strategies to facilitate driving in dementia. A study explored the attitudes of eighteen senior drivers towards ADAS but did not outline their comorbidities or assess the participant’s cognitive status. There were mixed opinions. The most popular features were the blind spot alert and automatic headlights, which compensate mainly for physical impairments including reduced neck mobility and reduced vision. On the other hand, the most concerning feature included the inaccurate feedbacks of ADAS in challenging environments like construction sites and bad weather [105]. Maneuvering challenging environments requires more cognitive input. However, the ADAS created more distractions in such cases, so its use would increase driving challenges for dementia patients. Moreover, participants received a one-to-three-hour training session before using the device. All reported a higher degree of confidence using the device after getting familiar with it. On the other hand, not everyone found the user manual easy to comprehend [105]. In real life, the user manual would be the only training provided which might not be enough for patients with dementia whose learning abilities are impaired, making the use of ADAS in such cases even more complex.

In addition, naturalistic driving assessment is quite time and resource consuming. Most studies using naturalistic driving analyzed results from camera footage during daytime only since image quality at night was low. As a result, standardized on-road tests have been devised as an alternative and are considered as the gold standard driving assessment in dementia [20,106]. In fact, results from this literature review showed that global ratings of driving safety and the types of errors did not differ significantly between on-road assessments and naturalistic driving in dementia participants (Level 1b). However, another study concluded that crash history was associated with error scores on the on road RIRT scores but not the naturalistic driving CDAS scores [47]. In fact, there are different scoring systems comprising of different behaviors, to assess road fitness to drive (Appendix B). The study conducted by Ott BR et al. (2012) used the CDAS score as an assessment method for both naturalistic and on-road testing in addition to the RIRT to minimize performance bias [64]. This identified only one cluster of important driving behaviors on on-road testing compared to two clusters in naturalistic driving assessment. It also explored the inter-rater agreement of two driving instructors. The inter-rater agreement was moderate for global pass/fail rating and low but not significant with regards to important items on the CDAS score. This translates into detection bias. Davis JD, Wang S, Festa EK et.al. (2018) introduced another assessment modality in addition to the RIRT and CDAS scores which are both dependent on instructor rating [68]. This consisted of the Modified Mockingbird Scoring system. This system uses an automated computerized analysis of the video recording of naturalistic driving (Appendix B). When compared to CDAS and RIRT scores, Mockingbird scoring system showed the highest accuracy (60.3% vs 72.9% vs 91.7 % respectively) to detect driving behavior in AD dementia. Sensitivity was the highest for the Mockingbird score even when corrected for milage (92.7% vs 74.4% RIRT vs 50% CDAS) and specificity was highest for CDAS scores (CDAS 87.5% vs 68.8% RIRT vs 33.3% Mockingbird).

Road and traffic conditions on standardized road testing are difficult to predict and may not be representative of true driving performance in certain scenarios. As a result, simulated driving was developed. This can simulate both rural and urban environments, evaluate particular driving maneuvers, assess response to surrounding environment or simulate high risk scenarios like traffic collisions [95]. However, elderly people may not be accustomed to using technological devices. As a result, the driver needs to learn how to use the simulator in the first place. This might be a challenge for dementia patients with impairment in memory and learning [96]. In fact, validity, and comparability of simulated driving with naturalistic driving is lacking and inconsistent when compared to on-road driving as outlined in section 4.4.2 of results. There is also a lack of standardization with regards to assessment via driving simulators [20]. Simulation sickness has also been described. In fact, a study identified a higher percentage of simulator sickness in older adults compared to a younger comparison group, especially when performing certain tasks [107]. Moreover, elderly people found it more difficult to adapt to the driving simulator environment since their performance improved significantly from the first to the fourth trial as opposed to younger individuals where it remained constant across the four trials.

### Office Based Driving Assessments

The above tests are often conducted in specialized facilities by trained instructors and often require a referral. This referral is often done by the patient’s physician. This led to the development of office-based driving assessments. History taking is often the first step in any medical consultation. As a result, participant and caregiver interviews can be a great source of information to assess the patient’s behaviors and driving abilities.

However, patients with cognitive impairment may lack insight into their driving problems so participant self-rating should be interpreted with caution [65, 72]. In fact, five studies assessing self-rated driving ability were identified in this review. Results were highly variable as shown in Table 9. All except one study, included participants with dementia of the AD subtype only. In fact, the only study which found no statistically significant correlation between participant self-rating and on road testing included participants with non-AD dementia [84]. On the other hand, three studies which included participants with AD dementia found either a negative correlation [72] or positive correlation [15, 77] between participant self-rating and on-road scores.

As a result, the physician might rely on the patient’s caregiver or family to inform them on the patient’s driving performance. However, one must take several factors into account including any caregiver agenda which might bias the report. In fact, two studies found no statistical correlation between caregiver rating and fitness to drive as defined by road testing [15, 76].

In a qualitative study conducted by Adler G (2010), many patients and caregivers reported that the patient would be more likely to stop driving if the advice was given by a health care professional [103]. However, most physicians do not feel comfortable approaching the subject due to various reasons [36]. As a result, the accuracy of clinician driving assessment should also be questioned and explored. Three studies also assessed clinician rating compared to on-road driving assessment. All three studies found a significant correlation with standardized road testing. The study conducted by Bixby K, Davis JD and Ott BR (2015), also compared clinician ratings with naturalistic driving. No significant association was found between the two ^[76]^. Ott BR, Anthony D, Papandonatos GD et.al. (2005) also investigated the predictive value of clinician interviews. The accuracy of these interviews ranged from 62-79% according to individual physicians [61]. This study also explored inter-rater accuracy between clinicians. The greatest accuracy was observed in clinicians with specialized training in dementia irrespective of the years of experience. Moreover, the following measures were given the highest weighting by the most accurate clinicians:

- Dementia duration;
- CDR and MMSE measures as predictors of disease
- Neuropsychological tests assessing praxis, visuospatial ability, executive function, and attention;
- History of accidents and traffic violation

Only one study compared participant self-rating, caregiver rating and clinician rating [78]. It concluded that participant self-rating had the least percentage correct classification compared to on-road pass/fail outcomes but highest NPV and sensitivity. On the other hand, clinician rating had the highest percentage correct classification and highest PPV. Despite this, PPV and specificity where less than 70%, indicating that negative results (i.e., unfit to drive) are much more accurate than positive results (i.e., fit to drive).

Assessment by clinicians may include the use of neurophysiological tests which provide a quantifiable measure of cognitive impairment. However, there are a multitude of tests which can be used, and different studies include a different combination of these. TMT-A, TMT-B and the MMSE were the most common tests explored. Results are explained and discussed in section 4.4.5. A systematic review assessed the relationship between cognitive tests and driving performance in dementia [108]. Similar results were obtained. In fact, they also identified the MMSE, TMT-B and TMT-A as the most commonly investigated tests. Inconsistent results were also found, with 56% of studies showing a significant correlation between MMSE and driving performance compared to 46.7% in this review. They also examined the use of cut-off scores. Only five out of thirty studies used cut-off scores, of which only three provided predictive equations [109-111]. These three studies were not included in this literature review since the diagnostic criteria used for dementia diagnosis were not specified. In fact, this review concluded that no single test was dependable to predict driving performance when used in isolation. On the other hand, the use of a composite battery of tests representing different cognitive domains is more accurate at predicting fitness to drive in dementia.

## Conclusions

Driving is an important skill which facilitates independence and autonomy, but it can be a dangerous weapon in the hands of an unskilled driver. The identification of common driving behaviors and assessments in dementia can help us identify drivers at risk. This will in turn help the medical team to devise strategies to improve the safety of the drivers themselves as well as those around them. Unfortunately, literature has shown that most physicians do not routinely approach the subject of driving cessation with patients with dementia. A reason for this being a perception of lack of knowledge, especially with regards to assessment [36-37].

Moreover, there are several guidelines regarding driving assessment in dementia. However, these vary in their advice as described in table twenty. As a result, this literature review was aimed at identifying available research on driving in dementia. The following conclusions were drawn:

1. Dementia effects driving performance (Grade B).
2. Attention, visuospatial skills, executive function, and global cognition are associated with driving competence and fitness to drive (Grade C).
3. Dementia disease severity effects driving performance (Grade C).
4. Higher disease severity is associated with a greater risk of unsafe driving (Grade C).
5. Moderate to severe disease (CDR≥2) is not compatible with safe driving (Grade C).
6. Inconsistent results were drawn with regards to driving safety in very mild and mild disease (CDR 0.5-1). Disease severity should not be an absolute contraindication for driving in mild disease stages (CDR0.5-1) but screening, formal assessment and regular follow-ups may still be indicated (Grade D).
7. Driving reassessment in dementia should occur at intervals of 6 months (Grade C).
8. Dementia etiology affects driving performance in different ways (Grade B).
9. Road assessments are reflective of naturalistic driving and should be considered the gold standard for driving assessment in dementia (Grade D).
10. Driving simulators are comparable to on-road tests for the assessment of fitness to drive in AD dementia but not in non-AD dementia (Grade D).
11. Participant and caregiver rating have a limited role in determining fitness to drive in dementia, but negative results are more reliable than positive ones (Grade D).
12. Neurophysiological tests play a role in assessing fitness to drive in all types of dementia. However, no one test has been identified as being predictive of fitness to drive. Instead, a combination of tests aimed at assessing multiple cognitive domains needed for driving competence, should be used (Grade D).

Most conclusions are of grade D evidence, due to the inconclusive or inconsistent nature of results. This is namely attributed to the great variability in methodology and assessment measures used. Moreover, there is a lack of standardization in driving assessments worldwide, so current research in driving assessment in dementia might depend on the familiarity of the authors with their local assessment methods. In fact, most studies compare two or three different methods, with on-road driving assessment being the most popular since it is considered as a gold standard assessment. However, even the latter is not standardized, and multiple scoring systems exist. In fact, most studies compare two or three different methods. In fact, different assessment tools have unique advantages and disadvantages. As a result, more research comparing multiple rather than one or two modes of driving assessments is needed.

Evidence on the effects of dementia severity on the predictive accuracy of different assessment tools is also lacking. Moreover, the majority of studies which assess the impact of dementia severity on driving quantified dementia severity by using the CDR score. However, the CDR score may not be the most commonly used score in clinical practice. As a result, studies assessing the impact of dementia severity on driving competence and assessment accuracy, might need to first identify the most commonly used tools to rate dementia in clinical practice and include them in the studies. In addition, dementia is a progressive disease, so longitudinal studies would be ideal to assess the progression of fitness to drive and the effect of disease severity on driving safety. These could also help us identify the optimal and most practical reassessment period at different stages of disease.

However only a few such studies were identified and included only participants with AD dementia. In fact, research on driving behaviors and assessments in drivers with dementia of the non-AD subtypes is lacking and of poor quality. Only two studies compared all four subtypes, and the population of non-AD participants was very low and unlikely to be representative of the whole population. In addition, these studies have shown variable results amongst different assessments in different subtypes. This highlights the need for further research in this area.

Dementia is a progressive condition with no disease modifiable treatment so advanced planning and symptom management are the hallmark of treatment. In addition, a gradual risk management approach via early and continuous discussion on driving cessation can help the patient and their families make the necessary transportation arrangements. This is more effective than crisis management which can be dangerous for both patients and others around them. Moreover, dementia is associated with loss of mental capacity in later stages, so discussions of driving cessation in early disease will empower the patient to make decisions about their driving. Such discussions could also serve as a smooth transition where safety measures are put into place to prolong driving cessation as much as possible. Unfortunately, research on compensatory strategies for driving in dementia is also lacking, including the role of advanced driver assistance systems [25].

In conclusion, research on driving and dementia is scary and of questionable quality, so its application in clinical practice is limited. As a result, like with any other form of assessment in geriatric medicine, driving assessment should involve the multidisciplinary approach. The multidisciplinary team consists of various healthcare professionals who together can identify both dementia related and non-dementia related factors that may jeopardize driving safety [112]. Most importantly, before or when a driving cessation advice is given, alternative modes of transportation should be arranged to safeguard the patient’s social well-being. Moreover, the types of resources and laws in a particular country/region will also impact on the methods of assessment used. As a result, evidence-based medicine together with patient centered care will help us make the best interest decision for the individual patient.

## Supplementary Materials

The Supplementary data can be found online at: www.aginganddisease.org/EN/10.14336/AD.2022.1126.


