# Supplementary material for: Driving Assessment for Persons with Dementia: How and when?
Source: Aging Dis. 2023 Jun 1;14(3):621–51. doi: 10.14336/AD.2022.1126 (PMC10187703; doi:10.14336/AD.2022.1126)
Supplement: Supplementary file 1 [file AD-14-3-621-s.pdf]

SUPPLEMENTARY DATA

Driving Assessment for Persons with Dementia: How and when?

Lara Camilleri[1*], David Whitehead[2]

© 2022. Camilleri L & Whitehead D. Published online at http://www.aginganddisease.org/EN/10.14336/AD.2022.1126

SUPPLEMENTARY DATA

Supplementary Table 1. Theoretical Models of Driving Behaviors.

Model type	Description	Components
**Information processing Models**	These are often linear models depicting progression from one stage to another which underestimate the true complexity of the driving process	They explore multiple cognitive domains including perception style, reaction time, attention, vigilance and decision making
**Cybernetic models**	It describes how the sensory environmental input affects the driver's processing and decision making which are reflected in voluntary motor action directing car movement. Figure 1 is an example of how this model can be applied to driver steering movement	Divided into 4 main parts:<br>1. Anticipatory module – responsible for input from visual stimuli of the road's geometry and environment from a far point<br>2. Compensatory module – responsible for input of the road geometry and environment but from a near points<br>3. Processing time and speed of the input information<br>4. Neuromuscular system - produces the output based on the stimuli and processing information
**Hierarchal control models**	Namely focus on the interaction between the driver and their environment.	Two main models:<br>1. The skill-rule-knowledge model identifies different levels of driving behaviours which can categorise driving errors as shown in figure 2.<br>2. The three-level hierarchical model divides its three levels strategical, tactical and operational levels.
**Motivational Model**	These models focus on the driver's performance based on the driver's subjective risk-taking behaviour in relation to the particular environment or situation.	

Supplementary Table 2. Summary of Primary Observational Studies Related to Driving Behaviours in Dementia.

Author and year of publication	Study objectives	Study population	Dementia Diagnostic criteria	Cognitive and Visual tests	Driving measures and outcomes	Results and conclusion
Aksan N, Anderson SW, Dawson J et.al. (2014),	1) To determine whether driving competence is dependent on performance in secondary driving tasks.<br><br>2) To assess whether cognitive status affects driving outcomes.	32 Probable AD<br><br>39 PD<br><br>77 Healthy Controls (HC)	NINCDS-ADRDA criteria	Grooved Peg Board, TMT-A, UFOV CFT-C, JLO, Blocks, BVRT-E, COWA, AVLT, CFT-R, TMT-B, Contrast sensitivity (CS), Visual acuity	**Standardised Road Test:**<br><br>Navigation-related secondary task performance (route-following and landmark/ sign identification tasks),<br><br>On-task safety errors,<br><br>Baseline safety errors.	AD patients were worse than PD patients at identifying stop signs. The diseased group (AD + PD) performed worse than the healthy group in global secondary task performance, global landmark/sign identification, and global on task safety route following, baseline safety, lane observance, turns, visuosensory function, visuospatial construction, memory, processing speed and executive functioning.<br><br>Visuospatial construction predicted baseline safety errors per mile. Processing speed predicted on-task

© 2022. Camilleri L & Whitehead D. Published online at http://www.aginganddisease.org/EN/10.14336/AD.2022.1126

						safety errors. Visual sensory functioning, memory and executive function predicted safety in navigation-related secondary task performance.
Crivelli L, Russo MJ, Farez MF, Bonetto M, Prado C et.al. (2019)	To produce a driving assessment protocol for drivers with mild dementia by identifying the neurophysiological tests that best predict driving competence.	28 Mild AD (CDR 1.0)<br><br>28 Healthy Controls (HC)	NINCDS-ADRDA criteria for AD. CDR scale for severity rating.	MMSE<br>Logical Memory Immediate and Logical Memory Delayed<br>Digit Span Forward and Backward,<br>TMT-A,<br>DSMT,<br>Category Fluency,<br>Boston Naming,<br>NPI-Q,<br>FAQ,<br>FDS	**Standardised Road Test**<br><br>**Caregiver and Participant Perception of driving ability**.<br><br>**Simulator driving assessment**: Assessed traffic signal reaction tasks and brake reaction tasks,<br><br>**Traffic signal recognition task**.	AD drivers made more mistakes than HC during the on-road driving assessment and the traffic signal recognition score. AD patients also had lower traffic signal reaction scores and brake time reaction scores on simulator test. Difficulty in recognising the following signs was more prominent in the AD group:<br>"No entry for vehicles"<br>"U-turn prohibited"<br>"No overtaking"<br>"Narrow road"<br>"Pedestrian crossing"
Croston J, Meuser TM, Berg-Weger M et.al. (2009)	1) Identify abnormal driving behaviour in drivers with dementia near the time of cessation.<br><br>2) To investigate the caregiver's perspective regarding driving cessation.	119 Mild to Moderate Dementia of AD, FTD or DLB type<br><br>(CDR 0.5 - 2)	DSM-IV criteria for dementia diagnosis<br><br>NINCDS-ADRDA criteria for AD.<br><br>CDR scale for severity rating.	CDR<br>MMSE	**Caregiver Questionnaire**:<br><br>Driving Habits Questionnaire for the driver's crash history, driving status and trips per week.<br><br>19-item questionnaire covering various driving behaviours:<br>Monitoring for traffic,<br>Speed control,<br>Turns or intersections,<br>Backing up,<br>Lane Keeping,<br>Following traffic signals or signs,<br>Parking,<br>Yielding,<br>Use of gas/brake pedals.	Only 29% of drivers with dementia were active drivers.<br>53% of caregivers rated the dementia participants' driving ability as fair, poor or unsafe prior to driving cessation.<br><br>Behaviours that were more common before cessation included:<br>Drove closer to home,<br>Reduced frequency of driving;<br><br>70% reported at least one abnormal driving behaviour, the commonest being failure to monitor traffic followed by speed maintenance, difficulty with intersections or turns, backing up and lane maintenance.<br><br>Reasons for driving cessation included worsening cognition (54%), caregiver's/family's advice (42%), physician's advice (40%) and MVC (23.5%).<br><br>Barriers to driving cessation included lack of insight or personality issues in the driver with dementia (33%) followed by caregiver belief of good driving capacity (17%) and risk of social isolation (14%).

© 2022. Camilleri L & Whitehead D. Published online at http://www.aginganddisease.org/EN/10.14336/AD.2022.1126

SUPPLEMENTARY DATA

Davis JD, Wang S, Festa EK et.al. (2018)	To identify unsafe behaviours or distracted driving with a potentially high crash risk in cognitively impaired drivers.	44 possible or probable AD (CDR 0.5 - 1)<br><br>16 Healthy controls (HC)	NINCDS-ADRDA criteria for AD.<br><br>CDR scale for severity rating.	MMSE<br>TMT-A,<br>TMT-B<br>Maze-drawing<br>CDT	**Rhode Island Road Test (RIRT)**<br><br>**Naturalistic Driving Assessment**: CDAS was used.<br><br>**Automated computerised analysis of naturalistic driving** The Modified Mockingbird Event Scoring System was used.<br><br>**Miles driven per week and crash history.**	AD patients drove fewer miles/day when compared to HC.<br><br>When controlling for mileage driven, AD participants had higher error scores than HC participants.<br><br>AD patients made more severe errors for lane maintenance and not looking far enough ahead to anticipate traffic situations and manoeuvres. HC participants made more severe errors for behaviors related to distraction, failing to keep an out, and generally riskier behaviors.
Davis JD, Babulal GM, Papandonatos GD et.al. (2020)	To identify driving errors in drivers with early AD.<br><br>To compare driving behaviors in drivers with early symptomatic AD to cognitively normal (CN) drivers with preclinical AD and healthy adults without evidence of AD.	11 AD dementia (CDR 0.5 - 1)<br><br>11 preclinical AD<br><br>11 Healthy controls (HC)	NINCDS-ADRDA criteria for AD.<br><br>CDR scale for severity rating.	MMSE<br>TMT-A and TMT-B	**Naturalistic Driving Assessment**: The following driving error categories were used:<br>Collision/ near collisions<br>Distractions<br>Awareness<br>Driver conduct<br>Fundamentals<br>Following too close<br>Driver's condition and drowsiness<br>Traffic violations<br>Error frequency and safety risk were measured.	Most common driving behaviours in AD dementia group:<br>Four collisions with static objects<br>Driving unbelted,<br>Late response,<br>Rolling stops,<br>Poor judgment,<br>Following too close,<br>Speeding,<br>Failing to scan intersections,<br>Too fast for conditions.<br><br>When controlled for miles driven, speeding was the most common driving behaviour in the HC and AD dementia groups while hard braking was the most common event in the preclinical AD group.
Dawson JD, Anderson SW, Uc EY et.al. (2009)	To determine whether performances on neurophysiological tests, visual perception, and motor function could predict driving safety in licensed drivers with probable AD	44 Mild Probable AD (Mean MMSE 26.5)<br><br>115 Healthy controls (HC)	NINCDS-ADRDA criteria for AD.<br><br>MMSE for severity rating.	Rey-Osterreith Complex Figure Test-Copy and Recall versions (CFT-Copy and CFT-Recall)<br>MMSE<br>WAIS-R Block Design component<br>BVRT<br>TMT-A and TMT-B<br>AVLT<br>JLO<br>COWA Test<br>UFOV<br>CS<br>FVA<br>NVA<br>SFM.	**Standardised on-road driving assessment using ARGOS instrumented vehicle**.<br><br>**Motor skills measures**: Functional Reach for balance impairment. The Grooved Pegboard task was used to measure dexterity and motor speed. TUG test.	There were more driving errors amongst AD drivers when compared to HC drivers (average of 42 versus 32.2). The mean differences between the AD and HC groups were 5.9 for total errors, 2.3 for serious errors, 3.7 for less serious errors and 5.9 for lane observance errors.<br><br>More errors were observed according to increasing age but not according to gender.<br><br>The following driving errors were statistically more common in the AD group when compared to the HC group:<br>Lane observance errors;

© 2022. Camilleri L & Whitehead D. Published online at http://www.aginganddisease.org/EN/10.14336/AD.2022.1126

						Failing to proceed through intersection even though the light had turned green.<br>AD drivers make more total safety errors, lane observance errors, and serious safety errors than elderly drivers without AD.<br><br>A neurophysiological test score combining performances across multiple cognitive domains, was a better predictor of driving safety in AD when compared to single domains. Visuospatial and motor response test were also good predictors of driving safety in AD.
de Simone V, Kaplan L, Patronas N et.al. (2007)	To examine the driving behaviour of FTD patients, especially the impact of personality changes and deficits of social cognition on driving safety in FTD patients.	15 FTD<br><br>15 Healthy controls (HC)	Consensus Criteria for Frontotemporal lobe degeneration.	Mattis Dementia Rating Scale<br>Phonemic Fluency,<br>Semantic Fluency,<br>Boston Naming Test,<br>Token Test,<br>WAIS-III<br>WMS-III<br>Neurobehavioral Rating Scale (NBRS).	**Brain MR images** for the distribution and severity of cerebral atrophy.<br><br>**Driving Simulator test**:<br>Total number of collisions,<br>Off-road accidents,<br>Traffic light and speeding tickets.<br>Haphazard stopping,<br>Running stop signs,<br>Unusual variations in speed.	Several FTD patients stopped driving for variable lengths of time throughout the simulation test.<br><br>FTD patients had greater speed variability, with significantly higher number of speeding tickets. FTD patients remembered fewer words on both the free recall and cued recognition tests.<br><br>Amongst FTD patients, 60% had collisions, 47% had off-road accidents, and 33% ran stop signs. In contrast, none of the control subjects ran a stop sign or had a collision, and only one had one off-road accident. Dementia severity was not correlated with any driving measure.

© 2022. Camilleri L & Whitehead D. Published online at http://www.aginganddisease.org/EN/10.14336/AD.2022.1126

SUPPLEMENTARY DATA

Duchek JM, Carr DB, Hunt L et.al. (2003)	To investigated longitudinal driving performance in healthy aging and early-stage Alzheimer's Dementia at a 6 monthly interval over 2 years.	58 Healthy controls (HC)<br><br>21 Very mild AD (CDR 0.5)<br><br>29 Mild AD (CDR 1.0)	NINCDS-ADRDA criteria for AD.<br><br>CDR scale for severity rating.	CDR	**Washington University Road Test (WURT)**<br>.	HC drivers took a significantly longer time to receive a rating of not safe compared to Mild AD patients (CDR 1). The time for the very mild AD drivers to become unsafe fell between HC and th mild AD groups but the difference was not statistically significant.<br><br>The driving behaviours that were statistically correlated with increasing dementia severity (higher CDR scores), were impairment in lane change and signals. The driving behaviours that showed a statistically significant decline over time were qualitative judgement and speed control but were not affected by CDR scores. There was a longitudinal decline in driving skills across all three groups of drivers.<br><br>Three driving behaviors showed a significant decline for all groups: qualitative judgments, reaction to others, and speed control. Baseline age was a significant risk factor for receiving a rating of not safe.
Economou A, Pavlou D, Beratis I, et al. (2020)	To identify driving variables that predict crash risk in drivers with mild AD.	90 Healthy Controls (HC)<br>37 MCI (CDR 0.5)<br><br>16 Mild AD Dementia (CDR 1)	DSM-IV and NINCDS-ADRDA criteria for AD dementia and MCI.<br><br>CDR for severity	Not applicable	**Driving Simulator test**<br>Two unexpected incidents occurred per driving condition.<br><br>The following measures were used:<br>Speed position measures - average speed, headway average and lateral positioning;<br>Variability measures - speed variability, headway variability and lateral positioning variability;<br>Time measures - Average reaction time.	Compared to the HC group, the MCI group showed significantly lower average speed and larger headway average in moderate traffic with distraction, and significantly larger average reaction time in high traffic with distraction. Accident probability did not differ among the three groups.<br><br>Drivers with mild AD differed consistently from both the control group and the MCI group in most driving variables in all four conditions as follows:<br>They drove slower;<br>Left larger headway distances;<br>Were slower to respond at unexpected incidents;<br>Showed smaller variability in average speed;<br>Showed larger variability in headway distance.

© 2022. Camilleri L & Whitehead D. Published online at http://www.aginganddisease.org/EN/10.14336/AD.2022.1126

SUPPLEMENTARY DATA

Fernandez R and Duffy CJ (2012)	To gain a better understanding of the neural mechanisms and behavioral implications of navigational impairments in older and AD drivers	9 Young Healthy Controls (YHC)<br><br>16 Older Healthy Controls (OHC)<br><br>15 Mild Probable AD	NINCDS-ADRDA criteria for AD.	Not applicable	**Visual neurophysiological studies**.<br><br>**Scalp EEG recordings.**<br><br>**Driving Simulator test**: Pattern coherence and motion speed were assessed.<br><br>**Dot Motion Simulated Optic Flow Paradigm**.	Performance accuracy was worse in AD participants when compared to OHC and YHC and it was the same between YNC and OHC participants. Reaction time was faster in YHC participants compared to AD participants. Reaction time amongst OHC did not differ from either YHC or AD participants. Impairment in attention does not affect coherence and speed increment responses. YHC have a good global motion processing, whereas OHC and AD participants rely more on local motion processing.<br><br>Aging delays optic flow N200s, whereas AD reduces their amplitude. Coherence increments evoke the largest responses in AD participants, but speed increments evoked only minimal responses suggesting loss of neuronal speed specificity.
Fuermaier ABM, Piersma D, de Waard D, Davidse RJ, de Groot J et.al. (2019)	To identify driving errors of AD patients in comparison with healthy controls.<br><br>To identify any differences in driving errors, neurophysiological scores and simulator scores in AD drivers who failed versus those who passed the on-road test.<br><br>To investigate whether driving errors differ between different types of dementias.	45 Healthy control (HC),<br><br>80 AD,<br><br>13 VaD,<br><br>9 Mixed (AD + VaD),<br><br>14 FTD,<br><br>23 DLB and PD<br><br>(Very mild to mild severity CDR < 2)	CDR for severity rating.<br><br>NINCDS-ADRDA criteria for AD.<br><br>NINDS-AIREN criteria for VaD.<br>International Consortium Criteria for FTD.<br>Criteria of the DLB consortium for DLB.<br><br>UK PDS Brain Bank Criteria for PD.	CDR<br>MMSE<br>Reaction Time S2<br>Hazard Perception test<br>Traffic Theory test<br>Clinical interviews	**A Standardised Road test using Test Ride Investigating Practical (TRIP)**<br><br>**Driving Simulator Test**: The following measures were included: minimum speed when approaching an intersection with traffic lights, the number of collisions in a ride with intersections and two merging manoeuvre (the deceleration of the rear car after merging and the time headway directly after merging).	57.5% of AD patients failed the on-road test compared with 11.1% of HC, 69% with VaD, 66% with mixed dementia, 50% with FTD and 35% with DLB/PD.<br><br>AD drivers had significantly lower scores when compared to HC drivers on all four TRIP subscales, especially tactical, visual and global outcomes.<br><br>The following are the TRIP items at which 50% or more of participants got an insufficient score per diagnosis:<br>AD: Position on the road, speed, observation, turning left, merging with a fast-moving stream of traffic, general impressions.<br><br>VaD: Position on the road, speed, observation, turning left, merging with a fast-moving stream of traffic, general impressions.<br><br>Mixed (AD + VaD): Position on the road, speed, observation, anticipation, turning left,

© 2022. Camilleri L & Whitehead D. Published online at http://www.aginganddisease.org/EN/10.14336/AD.2022.1126

						merging with a fast-moving stream of traffic, general impressions.<br><br>FTD: Position on the road, speed, observation, turning left, merging with a fast-moving stream of traffic, general impressions.<br><br>DLB/PD: Position on the road, turning left, merging with a fast-moving stream of traffic, general impressions.
Fujito R, Kamimura N, Ikeda M et.al. (2016)	To compare driving behaviours of patients with frontotemporal dementia with patients with Alzheimer's type dementia.	28 FTD<br><br>67 AD<br><br>(Very mild to moderate severity CDR 0.5 - 2)	Consensus Criteria for Frontotemporal lobe degeneration<br><br>NINCDS-ADRDA criteria for AD.	MMSE<br>CDR<br>IADL scale	**Caregiver Questionnaire**: Forgot the destination;<br>Failed to get the car in/out of the garage;<br>Difficulty judging inter-vehicle distances;<br>Ignored road and traffic signals; Distractions;<br>Showed overall changes in driving behaviour;<br>MVC history;	Driving behaviours which were statistically significantly higher in the FTD group compared to the AD groups included: increased difficulty in judging inter-vehicle distances, ignoring traffic signals, and distraction.<br><br>Driving behaviours which were statistically significantly higher in the AD group compared to the FTD group included an increased frequency of forgetting the destination. FTD patients had a significantly higher risk of causing an MVC compared to AD patients. Most common causes of MVC included rear-end collision due to distraction or poor judgement in inter-vehicle distance.<br><br>The mean time for an MVC from dementia diagnosis in FTD was 1.35 +/- 0.83 years compared to 3.0 +/- 1.36 years in the AD group ($P < 0.01$).
Frittelli C, Borghetti D, Ludice G et al (2009)	To explore the effects of AD and MCI on driving ability.	20 Mild Probable AD<br>(CDR 1)<br><br>20 MCI<br>(CDR 0.5)<br><br>19 Healthy Controls (HC)	NINCDS-ADRDA criteria for AD. Quality Standards Committee of the American Academy of Neurology Diagnostic Criteria for MCI.<br><br>CDR for severity rating.	MMSE<br>CDR<br><br>Self-administered SSS to obtain the daytime vigilance level.	**Driving Simulator Test**: The following measures were obtained: the length of run/ test duration, the number of infractions (speed limits violations and omission of stop at pedestrian crossings), number of stops at traffic lights, mean time to collision and the number of off-road events.<br><br>A **simple visual reaction time (S-VRT) test** to obtain the mean reaction time latency values.	SSS scores for all groups was 1, indicating that participants were "completely awake, active and alert". MMSE scores corresponded to CDR scores in all groups, but MMSE scores did not significantly correlate with driving performance in either of the three groups. AD participants had an overall worse driving performance when compared to both MCI and HC participants.<br><br>There was a significantly worse performance in the following behaviours in the AD group when

© 2022. Camilleri L & Whitehead D. Published online at http://www.aginganddisease.org/EN/10.14336/AD.2022.1126

						compared to the MCI and HC groups:<br>Length of run,<br>Mean time to collision,<br>Number of off road events.<br><br>S-VRT test: AD participants had significantly longer mean latencies when compared to boh MCI and HC participants.
Grace J, Amick M, D'Abreu A, et.al. (2005)	To assess how motor and cognitive functions in drivers with PD and AD affect driving performance.	21 Cognitively Intact PD<br><br>21 Mild AD (CDR 0 - 1)<br><br>21 Healthy Elderly Controls (HC)	Hoehn and Yahr staging and the motor section of the UPDRS for PD staging.<br><br>NINCDS-ADRDA criteria for AD.<br><br>MMSE for cognitive status in PD and HC.<br><br>CDR for severity rating in AD.	HVLT-R<br>ROCF<br>NAB Driving Scenes test<br>TMT-A and TMT-B<br>Computerized Mazes<br>Finger Tapping Test	**Washington University Road Test (WURT)**<br><br>**Participant/Informant Interview**: To ascertain number of miles driven per week, number of driving trips per week, history of moving violations and motor vehicle accidents over the past three years.	**Global ratings on on-road test**: 100% of HC drivers were safe compared to 67% of PD drivers and 45% of AD drivers.<br><br>**Specific errors on on-road test**: AD participants committed errors in all three categories, especially the tactical category. The most frequent errors included: lane changes, left turns and pulling over to the curb. Tactical errors were also common among PD participants, but these committed less operational and strategic errors when compared to the AD group. HC participants made very few errors in all three categories.<br><br>AD participants were more likely to make driving errors or be unsafe drivers when compared to both PD and HC participants. Tactical errors were most common across all three groups but only the AD group committed strategic and operational errors.
Lovell RK and Russell KJ (2005)	To develop criteria for formal driving assessment referral.<br><br>To investigate the value of routine driving assessment in patients with dementia.	20 dementia participants<br><br>(15 AD and 5 VaD/FTD)	DSM-V criteria for dementia diagnosis	MMSE	**Standardised road test**<br><br>**Off road assessment**: Driving history, medical history, drug history, vision and hearing, physical examination, cognition, reaction time and knowledge of road laws.	15 out of 20 participants failed initial assessment. There was no significant difference in MMSE scores or duration of disease between pass and fail groups. However, participants in the fail group were significantly older compared to the pass group.<br><br>Instructor interventions were significantly higher in the fail group.<br>The greatest number of interventions in the fail group occurred in the car park when the participant was asked to park or get out of the car park. Other interventions in the fail

© 2022. Camilleri L & Whitehead D. Published online at http://www.aginganddisease.org/EN/10.14336/AD.2022.1126

						group included the following:<br>Traffic sign recognition;<br>Giving way to on-coming traffic;<br>Driving off-route.
Luzzi S, Cafazzo V, Damora A, Fabi K, Fringuelli FM et. al. (2015)	To investigate knowledge and neural correlates related to road sign recognition and route learning in AD and semantic dementia.	16 Healthy elderly controls (HC)<br><br>73 Mild-moderate AD.<br><br>22 Mild-moderate SD<br><br>(MMSE >18/30)	NINCDS-ADRDA criteria for AD.<br><br>Consensus Criteria for Frontotemporal lobe degeneration<br><br>MMSE for severity rating.	MMSE<br>VOSP Battery<br>Ideomotor praxis,<br>RFOC-Recall,<br>Bisyllabic word span, Corsi blocks.<br>Letter Fluency, Luria's motor sequence,<br>Stroop test.<br>Verbal fluency, Easy picture naming and reading, Easy word-picture matching,<br>PPTT.	**Tests exploring road sign knowledge**: Road sign naming and comprehension assessed.<br><br>**Tests exploring route finding**: A route learning test was performed by means of a videotape.<br><br>**Neuroimaging**: PET scan to assess 2-deoxy-2-fluoro-D-glucose (FDG) uptake in different brain areas.	**Road Sign Naming:** Patients with AD had significantly lower scores compared to HC participants. Patients with SD had significantly lower scores compared to both HC and AD participants.<br><br>**Road Sign Comprehension**: Patients with SD had significantly lower scores when compared to both HC and AD participants. There was no significant difference between AD and HC participants.<br><br>**Route learning Test**: Patients with AD showed a lower performance compared to both HC and SD participants. There was no significant difference between SD and HC participants.
Ott B.R, Heindel WC, Whelihan WM et.al. (2000)	To identify the underlying neurophysiology of driving impairment in drivers with a dementia diagnosis	79 Probable or Possible AD<br>(35 very mild,<br>28 mild<br>16 moderate severity)	NINCDS-ADRDA criteria for AD.<br><br>CDR for severity rating.	MMSE<br>CDT	**SPECT scans**<br><br>**Caregiver-rated driving ability scale**.<br><br>**Instrumental Activities of Daily Living (IADL) scale**.	Severity of driving impairment as assessed by caregiver rating, was significantly correlated with dementia severity quantified by higher CDR and lower IADL scores, but not related to global cognitive functions as measured by the MMSE.<br><br>SPECT changes related with temporoparietal regions were associated with milder driving impairment, whereas changes in the frontal regions were associated with more severe driving impairment.

© 2022. Camilleri L & Whitehead D. Published online at http://www.aginganddisease.org/EN/10.14336/AD.2022.1126

SUPPLEMENTARY DATA

Ott BR, Heindel WC, Papandonatos GD et.al. (2008) [2]	To identify the natural and progressive course of driving impairment in drivers with a mild dementia diagnosis	84 Early AD (CDR 0.5-1)<br><br>44 Healthy Controls (HC)	NINCDS-ADRDA criteria for AD.<br><br>CDR for severity rating.	CDR	**Washington University Road Test (WURT)**<br><br>**MVC and Traffic Violation History**: History of events over the past 3 years. A log of MVCs, traffic violations and miles driven, and trips taken per week was kept. This data was supplemented by state records.<br><br>AD participants underwent both office and road tests at 6-month intervals over 3 years. HC participants underwent both office and road tests at baseline and at 18 months.	At baseline, AD participants had a higher number of MVCs and traffic violations when compared to HC participants and performed more poorly on the road test. Over time, both HC and AD participants obtained worse on road scores when compared to baseline, but AD participants performed worse than HC participants.<br><br>All AD participants developed worsening CDR scores along the three years of the study indicating disease progression. In fact, 91.7% of AD participants had dropped out by 36 months. None of the HC participants had developed higher CDR scores at 18 months.<br><br>At 18 months, the number of unsafe on-road scores did not increase in the AD group, however there was a higher number of marginal scores. The hazard of failure in the CDR 1.0 group was 3.5 times higher than the CDR 0.5 group.<br><br>Increasing age, a higher CDR score and lower education levels were significant independent predictors of unsafe driving in AD patients.
Paire-Ficout L, Lafont S, Conte F, Coquillant A, Fabrigoule C et.al. (2018)	To identify tactical self-regulating behaviour in drivers with Alzheimer's Dementia during naturalistic driving.	20 Early AD (MMSE >24/30)<br><br>21 Healthy Elderly Controls (HC)	DSM-IV and NINCDS-ADRDA criteria for AD.<br><br>MMSE for severity rating.	TMT A<br>TMT B<br>Stroop test<br>DSST UFOV<br>159 Koh's blocks test	Naturalistic Driving Assessment: The Naturalistic Driving Assessment Scale (NaDAS) tool was used.<br><br>Participant Interview: number of miles driven per week, number of driving trips per week, main driver, new difficulties reported, and any avoidance of these driving difficulties.	AD participants scored worse than HC participants on all neurophysiological tests. Driving performance in the AD group was statistically worse than the HC group when measured with all the assessment scales. Critical events in the AD group occurred twice as often when compared to the HC group and were more likely to be unaware of the event.<br><br>AD participants experienced a poorer tactical self-regulatory behaviour which reflects impairment in the following driving manoeuvres:<br>Speed control,<br>Ensure safe distances

© 2022. Camilleri L & Whitehead D. Published online at http://www.aginganddisease.org/EN/10.14336/AD.2022.1126

						between vehicle and surrounding objects,<br>Changing<br>lanes,<br>Appropriately anticipate or plan actions.
Tomioka H, Yamagata B, Takahashi T et.al. (2009)	To assess the cognitive responses of AD drivers to high risk driving situations.	12 Mild AD (CDR 1.0)<br><br>14 Healthy Controls (HC)	DSM-IV and NINCDS-ADRDA criteria for AD.<br><br>CDR for severity rating.	MMSE<br>CDR	**Driving Simulator**: Four collision avoidance scenes were presented.<br><br>**Near-infrared spectroscopy (NIRS)**: Used to measure brain activity during the simulator test by detecting changes in cerebral blood flow. Bilateral frontal and temporal regions were examined.	The reaction time and force of breaking was significantly lower in the AD group when breaking was needed to avoid collision.<br><br>There was no significant difference in brain activity between AD and HC participants during routine driving. There was also no significant activation in the prefrontal cortices during baseline driving. During the collision avoidance scenario, there was a significant difference in frontal activation between HC and AD groups. Prefrontal activation was evident in all four scenarios in the HC groups but was diminished in the AD participants.<br><br>AD patients exhibit hypofrontality when faced with high risk situations which might impair their ability to recognize and responding to high risk situations during driving.
Uc EY, Rizzo M, Anderson SW et.al. (2004)	1. To assess whether AD drivers make more driving errors related route following tasks (RFT).<br><br>2. To assess whether visual and cognitive measure can predict navigation and safety errors in AD.	32 Mild AD (MMSE >18/30)<br><br>136 Healthy Controls (HC)	NINCDS-ADRDA criteria for AD.<br><br>MMSE for severity rating.	MMSE<br>Cogstat<br>JLO<br>ROCFT<br>AVLT-RECALL<br>WAIS-R Blocks Design BVRTT<br>TMT-B<br>COWA<br>UFOV<br>NVA<br>FVA<br>CS<br>SFM	**Standardised road test using ARGOS instrumented vehicle**<br><br>The following measures included:<br>1) Incorrect turns;<br>2) Times lost (incorrect turns which the driver did not recognise and correct);<br>3) At-fault safety errors.	AD drivers made significantly higher driving errors when compared to HC drivers. RFTs increased the likelihood of committing safety errors in AD drivers.<br><br>Common driving errors in the AD group included the following:<br>Incorrect turns;<br>Got lost;<br>At-risk safety errors particularly inappropriate lane crossing;<br>Longer duration to complete the test and learn the route;<br>More perception/attention and memory related errors compared to HC participants.
Uc EY, Rizzo M, Anderson SW et.al. (2006)	To assess the behaviour of drivers with AD when faced with a potential risk of rear-end collision.	61 Mild AD<br><br>115 Healthy Controls (HC)	NINCDS-ADRDA criteria for AD.	MMSE<br>Cogstat<br>JLO<br>ROCFT<br>AVLT-RECALL	**Driving Simulator test**: A scenario posing a risk of rear-end collision is presented to the participant.	89% of AD participants exhibited an improper response compared to 65% of HC participants, namely AD patients had a higher risk of stopping abruptly and permanently.

© 2022. Camilleri L & Whitehead D. Published online at http://www.aginganddisease.org/EN/10.14336/AD.2022.1126

				WAIS-R Block Design<br>BVRTT<br>TMT-B<br>COWA<br>UFOV<br>NVA<br>FVA<br>CS<br>SFM	The following measures were used:<br>First Reaction Time;<br>Occurrence of Improper Response (crashing, swerving out of lane or stopping abruptly and permanently).	The reaction time was also significantly slower in the AD group.<br><br>There was no statistically significant difference in crash rate between the AD and HC groups.
Uc EY, Rizzo M, Anderson SW et al. (2005)	To assess driving behaviour in AD drivers during a landmark and traffic sign identification task.	33 Mild Probable AD 137 Healthy Controls (HC)	NINCDS-ADRDA criteria for AD.	MMSE<br>Cogstat<br>JLO<br>ROCFT<br>AVLT-RECALL<br>WAIS-R Blocks Design<br>BVRTT<br>TMT-B<br>COWA<br>UFOV<br>NVA<br>FVA<br>CS<br>SFM	**Standardised On-road Driving assessment using ARGOS instrumented vehicle**<br><br>Divided in two parts: 'On-task' segment to assess landmark and traffic sign identification tasks (LTIT); and the 'no-task' segment typical of a standard road test.	AD participants identified a smaller percentage of restaurants and traffic signs and committed more at-fault safety errors during LTIT at a significant difference when compared to HC participants.<br>LTITs increased the likelihood of committing safety errors in AD drivers.
Vaux LM, Ni R, Rizzo M et.al. (2010)	To assess the ability of AD and PD patients to detect impending collisions.	6 Mild AD (Mean MMSE 24.5)<br><br>8 PD<br><br>18 Healthy Controls (HC)	NINCDS-ADRDA criteria for AD.<br><br>Diagnostic criteria described by Gelb DJ et.al. (1999) for PD.<br><br>MMSE for severity rating.	MMSE<br>Cogstat<br>JLO<br>ROCFT AVLT-RECALL<br>WAIS-R Blocks Design<br>BVRTT<br>TMT-B<br>COWA<br>UFOV<br>NVA<br>FVA<br>CS	**Self-Reported Driving Behaviour**: Number of miles and days driven per week, number of accidents in the past two years and number of times pulled over in the past two years.<br><br>**Collision Detection task**	**Collision Detection Performance:** AD participants performed significantly worse when compared to HC participants. The performance between AD and PD groups was not statistically significant. Worse outcomes were evident with increased number of objects and longer time to contact.
Venkatesan U et.al. (2018)	To investigate the relationship between driving and visuospatial search and binding in drivers with mild AD dementia.	42 Mild AD (CDR 0.5-1)<br><br>37 Healthy Controls (HC)	NINCDS-ADRDA criteria for AD.<br><br>CDR for severity rating.	MMSE<br>CDR<br>TMT-A and TMT-B<br>CDT	**Rhode Island Road Test (RIRT)**<br><br>**Naturalistic Driving**: The Composite Driving Assessment Scale (CDAS)<br><br>**Visual Search Task**: Luminance motion (L+M) and colour motion (C+M) targets presented.<br>Measures used:<br>1) Median reaction time<br>2) Mean hit rate;<br>3) Mean false alarm rate.	Compared to the HC group, AD participants scored significantly worse in the following:<br>1) All cognitive scores except TMT-A;<br>2) RIRT Total Errors and Driving Awareness;<br>3) CDAS Total Errors and Response to Traffic;<br>4) All Visual Search Tasks and performed worse in the C+M compared to the L+M tasks.<br><br>In HC participants, selective attention was the major predictor of driving performance, whereas in the AD group visual search under high binding demands was the major predictor of driving performance.
Wild K and Cottrell V (2003)	To investigate how impairment in awareness in AD drivers may affect driving behaviours.	15 Probable or Possible Mild AD (CDR 0.5-1)	NINCDS-ADRDA criteria for AD.<br><br>CDR for severity rating.	MMSE<br>CDR	**Standardised road test**.<br>The Driving Safety Evaluation:<br>Management of intersections,	Driving frequency was not significantly correlated to driving performance in either HC and AD groups.

		15 Healthy Controls (HC)			Lane changes,<br>Lane Maintenance,<br>Speed control,<br>Follows at safe distance,<br>Appropriate signalling,<br>Proper use of mirrors,<br>Responds to road conditions,<br>Responds to warning road signs,<br>Handles conversational distraction.<br><br>**Participant and Caregiver Rating**	AD drivers have a reduced awareness of cognitive and driving abilities. AD drivers performed significantly worse in all 10 items when compared to HC participants, with the greatest discrepancy amongst the following:<br>Responds to warning road signs,<br>Management of intersections,<br>Handles conversational distraction,<br>Proper use of mirrors,<br>Responds to road conditions.
Yamin S, Stinchcombe A, Gagnon S (2016)	To assess whether measures of attention, visual processing and global cognition are good predictors of driving performance in AD drivers.	20 Probable Mild AD (GDR stage 3)<br><br>21 Healthy Controls (HC)	NINCDS-ADRDA criteria for AD.<br><br>GDR scale for severity rating.	MMSE<br>MDR Scale (DRS2 and DRS2 Alternate)<br>TEA<br>UFOV<br>VOSP<br>BNT	**Driving Simulator Test**:<br>1) Total number of errors recorded by the simulator<br>2) Structured rater score.<br>The following errors were recorded:<br>Center line crossings<br>Road edge excursions,<br>Failure to stop at a stop sign or red light,<br>Speeding,<br>Illegal turns,<br>Off-road crashes,<br>Vehicle collisions.	AD participants performed worse than HC participants on the simulator driving test. Errors that were statistically significantly higher in the AD group were the following:<br>Failure to stop at traffic lights;<br>Poor speed control (over speeding);<br>Total number of centreline crossing;<br>Total number of crashes.<br><br>AD participants scored worse than HC participants in the following cognitive tasks at a statistically significant difference:<br>MMSE;<br>DRS;<br>Object perception subscale of the<br>VOSP;<br>TEA components of attention;<br>All three subsets of UFOV.
Stinchcombe A et.al. (2016)	To identify driving errors performed by mild AD and cognitively normal elderly drivers at intersections.	17 Mild AD (GDS 3 - 4)<br><br>21 Healthy Controls (HC)	NINCDS-ADRDA criteria for AD.<br><br>Global Deterioration Scale (GDS) was used to assess severity.	MMSE	**Driving Simulator Test**: Driving errors were grouped into attentional, perceptual, judgment, or coordination.<br><br>Driving errors were further classified according to location in respect to intersection as follows:<br>Preparation errors;<br>Execution errors;<br>Recovery errors;<br>Initiation errors and Crashes.	**Preparation errors**: Most common errors in the AD group were inappropriate use of brake, poor speed control, lack of lane maintenance and non-compliance to traffic signs and lights<br>**Execution errors**: Most common errors in the AD group during this phase were poor vehicle control and non-compliance to traffic light signals.<br>**Recovery errors**: The only significant error in the AD group during this phase was loss of vehicle control.<br>**Initiation errors and Crashes**: Most common errors in the AD group were inability to follow

© 2022. Camilleri L & Whitehead D. Published online at http://www.aginganddisease.org/EN/10.14336/AD.2022.1126

						simulator's auditory instructions, inability to execute the turn and drive off the road. AD drivers were three times more likely than HC participants to crash in or around an intersection.

Supplementar Table 3. Description of Primary Observational Studies on Driving Assessments in Dementia**.**

**Author and year of publication**	**Study Aims**	**Study population**	**Dementia Diagnostic criteria**	**Cognitive and Visual Tests**	**Driving Measures and Outcomes**	**Results and conclusion**
Barrash J, Stillman A, Anderson SW et.al. (2010)	To investigate the effect of demographic adjustments on predictive accuracy of neurophysiological tests for driving assessment in AD and PD.	26 Very Mild Probable AD<br><br>(MMSE >26/30)<br><br>33 PD<br><br>24 Healthy controls (NC)	NINCDS-ADRDA criteria for AD. MMSE for severity rating.	TMT-A TMT-B<br>CFT<br>BVRT<br>BD	**Standardised road test using ARGOS instrumented vehicle**	AD had worse neurophysiological scores than NC (but not PD) on four measures, and PD had worse scores than NC (but not AD) on all five measures. AD and PD did not have significantly more driving errors than the cognitively normal controls.<br><br>Raw scores were significantly correlated with driving errors for TMT-A, CFT and BD but adjusted scores were only significantly correlated to driving errors for TMT-A. Overall, the individual and collective correlation between neuropsychological measures (raw scores) and driving performance was low to moderate.
Bixby K, Davis JD, Ott BR (2015),	To evaluate the accuracy of clinician and caregiver ratings of driving competence compared to on-road driving assessment and naturalistic driving behaviour.	75 Mild to Moderate Possible or Probable AD.<br><br>CDR<br>0.5: n=35,<br>1.0: n =33<br>2.0: n=5.	NINCDS-ADRDA criteria for AD.<br><br>CDR scale for severity rating.	MMSE<br>CDR	**The Rhode Island Road Test (RIRT)**<br><br>**Naturalistic Driving** using CDAS.<br><br>**Caregiver and Clinician rating of driving ability**.	Clinician ratings were significantly but poorly associated with road test error scores but not with naturalistic driving error scores or global ratings. Global caregiver ratings were not significantly correlated with either road test or naturalistic driving error scores or global ratings. When results were analysed according to caregivers' relationship (spouse vs. adult child), adult children's global ratings and ratings of specific driving behaviors

SUPPLEMENTARY DATA

						were more closely related to road test performance than those of spouses. In fact, the spouses', their global rating was inversely correlated with global on road driving error scores.<br><br>Clinician ratings are more accurate than caregiver ratings especially if the caregiver is a spouse. If caregiver questionnaires are used this should focus on specific driving behaviours rather than overall global ratings.
Brown LB, Stern RA, Cahn-Weiner DA, Rogers B, Messer MA et.al. (2005)	To assess the ecological validity of the NAB Driving Scenes by comparing it with a standardised road test performance.	31 Very mild Possible or Probable AD (CDR 0.5)<br><br>24 healthy elderly controls (HC).	NINCDS-ADRDA criteria for AD.<br><br>CDR scale for severity rating.	NAB Driving Scenes test	The **Washington University Road test (WURT)**	The HC group performed significantly better than the AD group on both the on-road driving test and the NAB Driving Scenes test. There was a strong correlation between the on-road test scores and NAB scores in both HC and AD groups. The NAB Driving Scenes test was able to correctly classify 66% of the population as safe, marginal or unsafe drivers.
Brown LB, Ott BR, Papandonatos GD et.al. (2005)	To assess whether information from the patient, informant or physician (neurologist) is most accurate at predicting driving fitness in AD.	50 AD (33 CDR 0.5 and 17 CDR 1.0)<br><br>25 Healthy elderly controls (HC)	NINCDS-ADRDA criteria for AD. CDR scale for severity rating.	CDR MMSE	**Washington University Road test (WURT)**<br><br>**Physician, Patient and Informant rating**.<br><br>**Number of accidents and traffic violations** over the previous 3 years.	The HC group performed significantly better than both AD group on the on-road driving test but there was no statistically significant difference between the very mild (CDR 0.5) and mild (CDR 1.0) AD groups.<br><br>When compared to on road test scores, physician rating was most accurate followed by informant rating, with participant rating being the least accurate (in both very mild and mild AD groups).
Burns T, Lawler K, Lawler D et.al. (2018)	1) To assess the predictive value of the CPT for driving competence.<br><br>2) To assess the accuracy of cognitive screening tools in classifying cognitive impairment as mild versus major.	9 Healthy Controls<br><br>13 MCI<br><br>52 AD<br><br>17 non-AD dementia	DSM-V Criteria for dementia diagnosis.	CPT, MMSE, MoCA, LACLS	A **Standardised Road test** On-road measures not described.	Sensitivity and specificity of neurophysiological tests to identify drivers who should stop driving in the whole population:<br>CPT (cut-off score 4.7) - sensitivity 89% and specificity 75%<br>LACLS (cut-off score 4.7) - sensitivity 86% and specificity 60%<br>MOCA (cut-off score 16) - sensitivity 50% and specificity 89%<br>MMSE (cut-off score 19) - sensitivity 42% and specificity 89%

© 2022. Camilleri L & Whitehead D. Published online at http://www.aginganddisease.org/EN/10.14336/AD.2022.1126

SUPPLEMENTARY DATA

						The CPT was most accurate at identifying fitness to drive and correctly classifying mild from major neurocognitive impairment.
Crivelli L, Russo MJ, Farez MF, Bonetto M, Prado C et.al. (2019)	To produce a driving assessment protocol for drivers with mild dementia by identifying the neurophysiological tests that best predict driving competence.	28 Mild AD (CDR 1.0)<br><br>28 Healthy Controls	NINCDS-ADRDA criteria for AD.<br><br>CDR scale for severity rating.	MMSE, Logical Memory Immediate and Logical Memory Delayed, Digit Span Forward and Backward, TMT-A, TMT-B DSMT, Category Fluency BNT, NPI-Q FAQ FDS	**Standardised Road Test:** Driver's general behaviour, compliance with traffic signals, ability to complete the itinerary, capacity to follow instructor's directions.<br><br>**Caregiver and Participant Perception of driving ability** via AANQ<br><br>**Simulator driving assessment**: traffic signal reaction tasks and brake reaction tasks.<br><br>**Traffic signal recognition task**	AD patients performed worse on cognitive assessment when compared to HC. The driving questionnaires revealed no difference in driving ability between AD and HC group in both participant and caregiver answers.<br><br>The TMT-B, Verbal semantic Fluency and FDS were identified as the most important predictors of driving performance which statistically significant correlation with all driving scores. The TMT-A had a statistically significant correlation with both simulator tests and on road test but not the traffic signal recognition task.The MMSE, Logical Memory, DSMT, BNT, RAVLT, ROCFT, FAB, NPI-Q and FAQ had a statistically significant correlation with the on-road testing, traffic signal recognition task and the simulator traffic signal reaction task but not brake reaction task.<br><br>Age, DSMT and BNT were the variables that best predicted performance on the driving test. No single variable was enough or better than the other to obtain a cut off score but a good performance on two of these test was an indicator of good driving performance.
Davis JD, Papandonates GD, Miller LA et.al. (2012)	To compare road test performance to video recorded naturalistic driving in cognitively impaired drivers.	59 Possible or Probable AD (41 CDR 0.5 and 18 CDR 1.0)<br><br>44 Healthy participants (HC)	NINCDS-ADRDA criteria for AD.<br><br>CDR scale for severity rating.	Not applicable	**The Rhode Island Road Test (RIRT)**<br><br>**Miles driven per week and crash history**<br><br>**Naturalistic driving Behaviour**: CDAS was used.	AD participants made more errors and had worse ratings when compared to HC. AD participants had a higher error score severity in naturalistic driving when compared to on-road testing.<br><br>Crash history was associated with error scores on the on-road test

						results but not the naturalistic driving assessment.<br><br>A lower MMSE score was associated with more driving errors on both on-road and naturalistic assessments.<br><br>Both on-road and naturalistic driving assessments are sensitive to cognitive impairment irrespective of the age. Driving performance on on-road testing is a reasonable reflection of the naturalistic driving behaviour in cognitively impaired patients.
Davis JD, Wang S, Festa EK et.al. (2018)	To identify unsafe behaviours or distracted driving with a potentially high crash risk in cognitively impaired drivers.	44 possible or probable AD (CDR 0.5 - 1)<br><br>16 Healthy controls (HC)	NINCDS-ADRDA criteria for AD.<br><br>CDR scale for severity rating.	MMSE<br>TMT-A,<br>TMT-B<br>Maze-drawing<br>CDT	The **Rhode Island Road Test (RIRT)**<br><br>**Naturalistic Driving Assessment**: CDAS used.<br><br>**Automated computerised analysis of naturalistic driving** The Modified Mockingbird Event Scoring System was used.<br><br>**Miles driven per week and crash history.**	Total error score, corrected for mileage, was significantly correlated with the road test error score. Mockingbird error scores from automated analysis, was correlated with clinic measures of cognitive functioning with a modest correlations between error scores and MMSE measures, time to perform mazes, and number of errors on mazes. There was no relationship between error scores and CDT, TMT-A, TMT-B. The Mockingbird scoring of discrete events in automated analysis, achieved the highest sensitivity and specificity in predicting AD diagnosis.<br><br>The automated, event-based method is a valid method for driving assessment in AD, but it is more clinically informative when examining multiple relevant behaviors simultaneously rather than relying on global error scores. In-office cognitive measures had significant, but modest, associations with driving errors.
Davis JD, Babulal GM, Papandonatos GD et.al. (2020)	To identify driving errors in drivers with early AD.<br><br>To compare driving behaviors drivers with early symptomatic AD to cognitively	11 AD dementia (CDR 0.5 - 1)<br><br>11 preclinical AD	NINCDS-ADRDA criteria for AD.<br><br>CDR scale for severity rating.	MMSE<br>TMT-A and TMT-B	**Naturalistic Driving Assessment**<br><br>Collision/ near collisions<br>Distractions<br>Awareness<br>Driver conduct including judgement	MMSE and TMT-B scores were not significantly related to frequency of driving events.<br><br>TMT-A scores were significantly related to

	normal (CN) drivers with preclinical AD and healthy adults without evidence of AD.	11 Healthy controls (HC)			and reckless behaviour<br>Fundamentals<br>Following too close<br>Driver's condition and drowsiness<br>Traffic violations<br>Error frequency and safety risk were measured.	driving event frequency in a non-linear manner, specifically speeding events and hard acceleration events.<br><br>An event-based approach to naturalistic driving assessment, may be a more sensitive and practical method to identify risky driving behaviour in AD dementia and indicate a transition from preclinical AD to early AD dementia.
Dawson JD, Anderson SW, Uc EY et.al. (2009)	To determine whether performances on neurophysiological tests, visual perception, and motor function could predict driving safety in licensed drivers with probable AD	44 Mild Probable AD (Mean MMSE 26.5)<br><br>115 Healthy controls (HC)	NINCDS-ADRDA criteria for AD.<br><br>MMSE for severity rating.	ROCFT<br>MMSE<br>WAIS-R BD<br>BVRT<br>TMT-A<br>TMT-B<br>AVLT<br>JLO<br>COWA<br>UFOV<br>CS,<br>FVA<br>NVA<br>SFM	**Standardised on-road driving assessment using ARGOS instrumented vehicle**.<br><br>**Motor skills measures**: Functional Reach for balance impairment. The timed Get-Up-and-Go test. The Grooved Pegboard task was used to measure dexterity and motor speed.	A higher overall cognitive function was associated with a better safety error score in the AD group.<br>The following cognitive tests were significant predictors of driving errors in AD:<br>BVRT (working memory)<br>TMT-A (visual search and visual motor speed)<br>CFT-copy (visuoconstructional ability)<br>Functional Reach (motor function)<br><br>Lane observance errors were the most common type of errors in AD group, so cognitive test scores were compared to this type of error separately and the following cognitive tests showed significant correlation: CFT-COPY, UFOV-total and Functional Reach.
de Simone V, Kaplan L, Patronas N et.al. (2007)	To examine the driving behaviour of FTD patients especially the impact of personality changes and deficits of social cognition on driving safety in FTD patients.	15 FTD drivers<br>15 Healthy controls (HC)	Consensus Criteria for Frontotemporal lobe degeneration.	MDR<br>Phonemic Fluency,<br>Semantic Fluency,<br>BNT,<br>Token Test,<br>WAIS-III<br>WMS-III<br>Neurobehavioral Rating Scale (NBRS).	**Brain MR images** for the distribution and severity of cerebral atrophy.<br><br>**Driving Simulator test**:<br>Total number of collisions,<br>Off-road accidents,<br>Traffic light and speeding tickets.<br>Haphazard stopping,<br>Running stop signs, unusual variations in speed.<br>Sign recall.	Dementia severity was not correlated with any driving measure. However, severity of frontal atrophy was positively correlated with the NBRS total score.<br><br>Speeding and collisions were related to the patients' agitated and aggressive behaviour as measured by the NBRS. Neuropsychiatric symptoms that are measured in the agitation factor include disinhibition, aggression, hostility, excitement and of course agitation.
Fuermaier ABM, Piersma D, de Waard D, Davidse RJ,	To identify driving errors of AD patients in comparison with healthy controls.	45 Healthy Older Control (HC),	CDR for severity.<br><br>NINCDS-	CDR<br>MMSE<br>Reaction Time S2<br>Hazard	**A Standardised Road test using Test Ride Investigating Practical (TRIP)**.	All four 'off-road' predictor variables (i.e., clinical interviews, neurophysiological test,

de Groot J et.al. (2019)	To identify any differences in driving errors, neurophysiological scores and simulator scores in AD drivers who failed versus those who passed the on-road test. To investigate whether driving errors differ between different types of dementias.	80 AD,<br><br>13 VaD,<br><br>9 Mixed (AD + VaD),<br><br>14 FTD,<br><br>23 DLB and PD.<br><br>(Very mild to mild severity CDR < 2)	ADRDA criteria for AD.<br><br>NINDS-AIREN criteria for VaD.<br><br>International Consortium Criteria for FTD.<br><br>Criteria of the DLB consortium for DLB.<br><br>UK PDS Brain Bank Criteria for PD.	Perception test, Traffic Theory test, Clinical interviews	**Driving Simulator Test**: minimum speed when approaching an intersection with traffic lights, the number of collisions in a ride with intersections and two merging manoeuvre (the deceleration of the rear car after merging and the time headway directly after merging).	driving simulator test and the three combined), correlated significantly with all four T-RIP subscales in AD drivers. As a result, they were adequate predictors of driving performance in AD patients.
GRACE J, AMICK M, D'ABREU A, et.al. (2005)	To assess how motor and cognitive functions in drivers with PD and AD affect driving performance.	21 Cognitively Intact PD<br><br>21 Mild AD (CDR 0 - 1)<br><br>21 Healthy Elderly Controls (HC)	Hoehn and Yahr staging and the motor section of the UPDRS for PD.<br><br>NINCDS-ADRDA criteria for AD.<br><br>MMSE for cognitive status in PD and HC.<br><br>CDR for severity rating in AD.	HVLT-R<br>ROCFT,<br>NAB Driving Scenes test<br>TMT-A and TMT-B<br>Computerized Mazes<br>Finger Tapping Test	The **Washington University Road Test (WURT)**<br><br>**Participant/Informant Interview**: To ascertain number of miles driven per week, number of driving trips per week, history of moving violations and motor vehicle accidents over the past three years.	Predictive value of neurophysiological test compared to on-road testing:<br>**HVLT-R**: useful in distinguishing between safe and unsafe drivers in PD but not AD.<br>**ROCF**: Accurate in predicting driving safety in both AD and PD.<br>NAB Driving Scenes test: Not accurate in predicting driving status in AD and PD participants.<br>**TMT-A**: Accurate in predicting driving safety in the AD group only.<br>**TMT-B**: Accurate in predicting driving safety in both AD and PD groups, especially PD drivers.<br>**Computerized Mazes**: Not accurate in predicting driving safety in either.<br>**Finger tapping test**: Not accurate in predicting driving safety in either.<br><br>Global neurophysiological test performance was reduced in both AD and PD groups among unsafe drivers when compared to safe drivers.<br>The degree of memory impairment was not related to driving safety in AD drivers. Tests of executive and visuospatial function are most helpful in identifying at-risk drivers in AD. In fact, TMT (A and B) and ROCF were most accurate

© 2022. Camilleri L & Whitehead D. Published online at http://www.aginganddisease.org/EN/10.14336/AD.2022.1126

						at predicting driving safety.
Lafont S, Marin-Lamellet C, Paire-Ficout L et.al. (2010)	To identify cognitive tools that can accurately predict driving safety amongst individuals with different levels of cognitive functioning.	20 Early AD (Mean MMSE 26.4)<br><br>56 Healthy controls	DSM IV and NINCDS-ADRDA criteria for AD.<br><br>MMSE for severity rating.	MMSE BVRT, Isaacs's Set Test, Zazzo's Cancellation Test, DSST Computerized test battery.	**Standardised road test used.**<br><br>Driving evaluation was divided in three parts:<br><br>1) The Driving Researcher Score (DRS);<br><br>2) The Driving Instructor Intervention Score (DIIS);<br><br>3) The Driving Instructor Judgment (safe, borderline or unsafe).	30% of AD drivers were deemed unsafe compared to 1.8% of HCs.<br><br>**Relationships between the Three Driving Evaluations:** The Driving Researcher Score was strongly correlated with the Driving Instructor Intervention Score. Both the DRS and DIIS were correlated with the instructor judgement scores.<br><br>**Association between Cognitive Performances and Unsafe Driving:** The Go/No Go time was the only cognitive test significantly associated with a Driving Researcher Score > 31 penalties. Low performances in finger tapping and in the double task were significantly associated with a Driving Instructor Intervention Score >9. A low performance in finger-tapping and a low MMSE score were related to the unsafe Driving Instructor Judgment. A low performance on the Wechsler Digit Symbol Substitution Test and a high performance on the Stroop Interference Test were significantly associated with the composite indicator of unsafe driving.<br><br>The DSST is the most accurate test at predicting driving safety. A DSST score of 25 has the highest sensitivity of 92.2% with a specificity of 75% in predicting driving safety in AD.

© 2022. Camilleri L & Whitehead D. Published online at http://www.aginganddisease.org/EN/10.14336/AD.2022.1126

Lovell RK and Russell KJ (2005)	1) To develop criteria for formal driving assessment referral.<br><br>2) To investigate the value of routine driving assessment in patients with dementia.	15 AD,<br>5 VaD/FTD	DSM-V criteria	MMSE	**Standardised road test**<br>Participants who failed the initial test, coul repeat it in a familiar area.<br><br>**Off road assessment**: Driving history, medical history, drug history, vision and hearing assessment, physical examination, cognitive function, reaction time and knowledge of road laws.	15 out of 20 participants failed initial assessment. 12 out of the 15 who failed, underwent secondary on-road assessment in a local area and 10 of these passed. 9 out of 20 participants were re-assessed at 6 months (6 participants had stopped driving on their own accord in the interim). 5 out of 9 participants failed the on-road test at 6 months indicating disease deterioration. 2 participants who originally failed the test, passed at 6 months which could be explained by different environmental cues during the different test dates and fluctuation in cognition associated with AD.<br><br>Referral for driving assessment is recommended even in early stages of disease with 6 monthly re-assessments thereafter.
Luzzi S, Cafazzo V, Damora A, Fabi K, Fringuelli FM et. al. (2015)	To investigate knowledge and neural correlates related to road sign recognition and route learning in AD and semantic dementia.	16 Healthy controls (HC)<br><br>73 Mild-moderate AD.<br>22 Mild-moderate SD<br><br>(MMSE >18/30)	NINCDS-ADRDA criteria for AD.<br><br>Consensus Criteria for Frontotemporal lobe degeneration<br><br>MMSE for disease severity.	MMSE<br>VOSP, Ideomotor praxis, RFOC, Bisyllabic word span, Corsi blocks, Letter Fluency, Luria's motor sequence, Stroop test. Verbal fluency, Easy picture naming and reading, Easy word-picture matching, PPTT.	**Tests exploring road sign knowledge**: Road sign naming and comprehension assessed.<br><br>**Tests exploring route finding**: A route learning test was performed by means of a videotape.<br><br>**Neuroimaging**: PET scan to assess 2-deoxy-2-fluoro-D-glucose (FDG) uptake in different brain areas.	**Neurophysiological tests:** VOSP (silhouette component) and semantic fluency scores correlated significantly with road sign naming scores. VOSP (silhouette component), semantic fluency, single word comprehension and PPTT scores correlated significantly with road sign comprehension scores.<br><br>**Neuroimaging results**: In the SD group, there was a strong and significant correlation between FDG uptake in the left temporal area and the road sign naming test and road sign comprehension test. **I**n the AD group, there was a correlation between the road sign naming test and FDG uptake in a cluster centred on the posterior part of the parahippocampal gyrus close to the lingual gyrus. There was also a correlation between the performance on the route learning test and FDG uptake in the superior

						frontal gyrus/anterior cingulate.
Manning KJ et.al. (2014)	To assess and compare the ability of different CDT scoring systems in accurately assessing fitness to drive in cognitively impaired drivers.	47 Healthy Controls (HC)<br><br>75 Mild Possible or Probable AD<br><br>(CDR 0.5 - 1)	NINCDS-ADRDA criteria for AD. CDR scale for severity rating.	CDT with the following scoring systems:<br>7-point system<br>8-point system<br>10-poin t system<br>Conceptualized system	**The Rhode Island Road Test (RIRT)**	AD drivers had higher error scores, poorer global ratings on road tests and worse CDT scores when compared to HC participants.<br><br>The CDT on its own has limited clinical utility in predicting driving ability in both HC and AD patients. Diagnostic accuracy varied very slightly between different scoring systems. The 7-point scoring systems was the most accurate amongst the four scoring systems but still inaccurately classified 76% of AD participants when compared to on-road testing.
Ott BR, Heindel WC, Whelihan WM et.al. (2003)	To investigate the utility of neurophysiological tests in predicting driving safety in drivers with a dementia diagnosis.	Part A: 27 Probable or Possible AD<br>(18 CDR 0.5,<br>9 CDR 1.0)<br><br>Part B:<br>6 Healthy Controls (HC),<br><br>21 Probable AD,<br>11 MCI,<br>1 FTD,<br>1 Mixed Dementia	NINCDS-ADRDA criteria for AD.<br><br>CDR scale for severity rating.	Porteus Mazes errors (Years VIII & XII)<br>COWA<br>CDT<br>TMT- B<br>MMSE<br>FSIQ<br>Maze Tests (10 Mazes)	**Caregiver-rated driving ability scale**	In part A of the study, the time to complete the Proteus Maze Drawing was the only significant correlation to Caregiver-rated driver ability.<br><br>In part B of the study, the total score each of the 10 Maze tasks, dementia severity as measured by the CDR score, education and number of crashes, were significantly correlated to Caregiver-rated driving ability.
O'Brien HL, Tetewsky SJ, Avery LM et.al. (2001)	To analyse the perceptual mechanisms of visuospatial disorientation in AD.	26 Probable AD<br><br>50 Elderly Healthy Controls (EHC)<br><br>32 Young Healthy Controls (YHC)	NINCDS-ADRDA criteria for AD.	MMSE<br>Money Road Map Test,<br>WMS,<br>Figural Memory Test,<br>Verbal Paired Associates Test,<br>UFOV	**Standardised road test**<br><br>**Visual Discrimination Stimuli**: Left/Right Horizontal Motion Stimuli<br>Left/Right FOE Outward Radial Motion Stimuli<br>Left/Right Outward Motion Defined Boundary Stimuli<br>In/Out Centred FOE Radial Motion Stimuli<br>Stationary Square Location Stimuli<br><br>**Psychophysical protocol**: Visual stimuli were generated and eye movements were monitored via electro-oculogram electrodes.	AD subjects had impairment in left/right radial motion perception. 85% of AD and 33% of EHC exhibited impaired use of global radial patterns as opposed to YHC who did not exhibit any significant impairment.<br><br>Global pattern recognition is impaired first before clinically evident AD which forces all AD subjects to rely on local rather than global cues. Eventually, local motion processing also becomes impaired in clinically evident AD.<br><br>Road Map test scores were significantly related to left/right FOE outward

© 2022. Camilleri L & Whitehead D. Published online at http://www.aginganddisease.org/EN/10.14336/AD.2022.1126

					**Written Road Signs Test**.	radial motion in AD, suggesting that outward radial motion is linked to spatial cognition.<br><br>There was also a significant correlation between stationary visual stimuli threshold and figural and verbal memory test.<br><br>The keep-in-lane scores during the on-road test, correlated to the left/right FOE outwards radial motion thresholds in AD.<br><br>There was no significant correlation between other on-road measures, psychophysical measures and UFOV scores.
Ott BR, Anthony D, Papandonatos GD et.al. (2005)	To determine the validity and inter-rater reliability of clinician rating in determining driving fitness of drivers with dementia.	50 Mild Possible or Probable AD (CDR 0.5-1)<br><br>6 Clinicians	NINCDS-ADRDA criteria for AD.<br><br>CDR scale for severity rating.	CDR<br>MMSE	**The Washington University Road Test (WURT)**<br><br>**Participant, Informant and Physician rating of driving ability**<br><br>**Driving History**: Obtained from participants and informants.	When compared to the on-road driving instructor's score, clinician accuracy ranged from 62-78%.<br><br>There was great variability in specificity and sensitivity between clinicians. The greatest accuracy was observed in clinicians with specialised training in dementia irrespective of years of experience.<br><br>The following measures where given the highest weighting by the most accurate clinicians:<br>Dementia duration;<br>CDR and MMSE measures as predictors of disease<br>Neuropsychological tests assessing the following cognitive domains: praxis, visuospatial ability, executive function, and attention;<br>History of accidents and traffic violation.
Ott BR, Festa EK, Amick MM et.al. (2008) [1]	To analyse the accuracy of the Maze test in predicting on road driving performance in drivers with dementia.	65 Probable AD (CDR 0.5 - 1)<br><br>23 Possible AD (CDR 0.5 - 1)<br><br>45 Healthy Elderly	NINCDS-ADRDA criteria for AD.<br><br>CDR scale for severity rating.	Mazes (5 Computerised Maze Tasks)<br>MMSE<br>BQSS-ROCF,<br>TMT- A<br>TMT- B<br>Finger Tapping Test<br>HVLT	**The Washington University Road Test (WURT)** was used.	The total time to complete all the five mazes, correlated significantly with th total on-road test scores in both AD and HC participants.<br><br>There was a significantly high correlation between on-road test scores and TMT A and B and HVLT scores among the entire population, specifically

© 2022. Camilleri L & Whitehead D. Published online at http://www.aginganddisease.org/EN/10.14336/AD.2022.1126

		Controls (HC)				with HVLT and TMT A in AD participants.<br><br>Although a significant correlation was achieved, the Maze Test on its own is still not a good enough predictor of driving fitness in AD but if adjusted for age and combined with other neurophysiological tests like TMT-A and HVLT, it is a good predictor of on-road driving performance and may be used as a screening tool to identify at-risk AD drivers.
Ott BR et.al. (2012)	To compare on-road driving test performance with naturalistic driving performance of drivers with a dementia diagnosis.	42 Mild AD (CDR 0.5-1)<br><br>38 Healthy Controls (HC)	NINCDS-ADRDA criteria for AD.<br><br>CDR scale for severity rating.	CDR<br>MMSE	**The Rhode Island Road Test (RIRT)**<br><br>**Naturalistic Driving Assessment**:<br>The Composite Driving Assessment Scale (CDAS) was used.	Performance in the naturalistic setting was mostly correlated with two distinct aspects of driving namely, behaviors related to proper land keeping and responding to traffic and manoeuvring the vehicle. Performance on road test setting was correlated with one rather than the two driving behaviours, namely driving awareness items. Results were adjusted according to route difficulty, and these showed that similarity in the driving environment rather than measurement method (RIRT vs CDAS), is more significant to ensure a strong association between naturalistic and on road test performance. Driving skills assessed during on-road testing are not fully reflective of the full driving skills used during naturalistic driving.<br><br>As a result, road tests that stress proper lane keeping and response to traffic may be more representative of naturalistic driving ability.
Piersma D, Fuermaier ABM, De Waard D et.al. (2016)	To analyse and compare the predictive value of different forms of driving assessments in accurately determining driving fitness in drivers with Alzheimer's Dementia when compared to on-	81 AD (CDR 0.5 - 1)<br><br>45 Healthy Elderly Controls (HC)	NINCDS-ADRDA criteria for AD.<br><br>CDR for severity rating.	CDR<br>MMSE<br>TMT A TMT B<br>Two Maze Tests,<br>Reaction time S 1,2,3<br>Hazard perception test,<br>Traffic theory test,<br>ATAVT of the	**The Test Ride Investigating Practical fitness to drive (TRIP) protocol**<br><br>**Driving Simulator Test**: The following measures were included:<br>Minimum speed when approaching an intersection with traffic lights,	**On road driving test**:<br>50.6% of AD patients failed the on-road test compared to 4.4% of HC participants.<br><br>**Clinical interviews**:<br>Overall, The following aspects of the clinical interviews were significant correlates to fitness to drive in AD participants:

© 2022. Camilleri L & Whitehead D. Published online at http://www.aginganddisease.org/EN/10.14336/AD.2022.1126

	road driving measures.			Vienna Test System (VTS)	Number of collisions in a ride with intersections,<br>Two merging manoeuvres.<br><br>**Participant Interviews.**	CDR subscores: memory, orientation judgement and problem solving, community affairs and sum of box scores;<br>Informant's opinion of patient's driving safety;<br>Patient's judgement of one's own driving safety;<br>Recent driving experience.<br><br>**Neurophysiological tests**: AD patients achieved significantly lower scores in all tests except the drawings. The following test scores were significantly correlated with fitness to drive in AD participants: MMSE, Maze tests, drawings, TMT-A and TMT-B.<br><br>**Driving Simulator tests**: The following components of driving simulator test were significantly correlated with fitness to drive in AD participants:<br>Minimum speed at Intersection A and B,<br>Traffic lights reaction time,<br>Number of collisions,<br>Braking for car that pulls out,<br>Speed while merging,<br>Deceleration rear car,<br>Time headway merging.<br><br>Neuropsychological assessment (94.6% accuracy) was most accurate at identifying fitness to drive in AD, followed by driving simulator tests (85.6% accuracy), followed by clinical interviews (79.6% accuracy). The combination of these three measures provided the highest diagnostic accuracy (97.4% accuracy).
Piersma D, Fuermaier ABM, De Waard D et.al. (2018)	To investigate whether fitness-to-drive assessments used in AD patients is accurate in predicting fitness to drive in non-AD dementias namely FTD, VaD and DLB.	14 VaD<br><br>12 FTD<br><br>8 DLB<br><br>(CDR <2)	CDR for severity rating.<br><br>NINDS-AIREN criteria for VaD.<br><br>International Consortium Criteria for FTD.	CDR<br>MMSE<br>Reaction time S2,<br>Hazard perception test,<br>Traffic theory test	**Test Ride Investigating Practical fitness to drive (TRIP)**<br><br>**Driving Simulator Test**: The following measures were included:<br>Minimum speed when approaching an intersection with traffic lights,	4/14 patients with VaD, 5/12 patients with FTD, and 5/8 patients with DLB passed the on-road driving assessment.<br><br>Clinical interviews and the driving simulator test were not predictive of fitness to drive in patients with non-AD dementia when compared to on-road test scores, as

			Criteria of the DLB consortium for DLB.		Number of collisions in a ride with intersections,<br>Two merging manoeuvres.<br><br>**Participant Interviews**:	opposed to the neurophysiological tests.<br><br>The three tests used in combination, were not significant predictors of driving safety in non-AD dementia when compared to on-road test performance.<br><br>The three subgroups of dementia differed between themselves with regards to prediction variables:<br><br>VaD participants had worse predictor scores on both clinical interviews and neurophysiological assessments when compared to both FTD and DLB participants.<br><br>DLB participants had safer mean scores on all prediction variables when compared to both FTD and VaD participants.<br><br>FTD participants had a high safe self-judgment rate but were more likely than the other two groups to approach an intersection with traffic lights at high speed.<br><br>VaD drivers make more driving errors on road assessment while patients with FTD and DLB make more driving errors on driving simulator assessment.
Rizzo M et.al. (2001)	To assess whether drivers with mild AD are at greater risk of having MVC at intersections	18 Probable AD<br><br>12 Healthy Elderly Controls (HC)	NINCDS-ADRDA criteria for AD.	UFOV<br>WAIS-R, Block Designs and DSST,<br>COWA<br>ROCFT -Copy,<br>Facial Recognition Test (FRT)<br>BVRT<br>TMT- A<br>TMT B	**Iowa Driving Simulator**:<br>The following measures were assessed:<br>Steering wheel position,<br>Normalized accelerator and brake position,<br>Lateral and longitudinal acceleration,<br>Headway distance to the lead vehicle,<br>Time to collision,<br>Speed,<br>Number of crashes.	**Vehicle Control on road segment**: No safety errors related to lane crossings, shoulder crossings, speeding, or tailgating in either AD an HC groups.<br><br>**Crashes**: 33% of AD drivers experienced crashed at intersections compared to 0% of HC participants. Significantly predictive factors of crashes included: the Rey Osterreith CFT -(copy version), WAIS-R Block Design, TMT, motion perception, COWA, and overall cognitive status (ADSTAT). The ADSTAT score had the highest predictive value of crashes at intersections

SUPPLEMENTARY DATA

						in AD participants with a sensitivity of 75% and specificity of 94.7%.
Uc EY, Rizzo M, Anderson SW et.al. (2004)	1.) To assess whether AD drivers make more driving errors related route following tasks (RFT).<br>2.) To assess whether the cognitive load increases crash risk.<br><br>3.) To assess whether visual and cognitive measure predict errors in AD.	32 Mild AD<br><br>(MMSE >18/30)<br><br>136 Healthy Controls (HC)	NINCDS-ADRDA criteria for AD.<br><br>MMSE for severity rating.	MMSE<br>Cogstat<br>JLO<br>ROCFT<br>AVLT-RECALL<br>WAIS-R (Blocks Design subtest)<br>BVRTT<br>TMT-B<br>COWA<br>UFOV<br>NVA<br>FVA<br>CS<br>SFM	**Standardised road test using ARGOS instrumented vehicle.**<br><br>Divided in two parts: 'On-task' segment to assess route following tasks (RFT) and the 'no-task' segment typical of a standard road test. The following measures included:<br>1) Incorrect turns;<br>2) Times lost;<br>3) At-fault safety errors.	Cognitive scores related to verbal (AVLT-RECALL) and nonverbal memory (CFT-RECALL and BVRT), executive function (TMT-B and COWA), visual perception (FVA, NVA, CS, and JLO), visual attention (UFOVTOT), visuoconstructional abilities (CFT-COPY, BLOCKS), and overall cognitive function (COGSTAT) were significantly associated with RFT outcome measures.<br><br>AVLT-RECALL and UFOVTOT were significant predictors of incorrect turns and times lost.<br><br>AVLT-RECALL, CFT-COPY, and CS were significant predictors of at-fault safety errors.
Uc EY, Rizzo M, Anderson SW et.al. (2006)	To assess the behaviour of drivers with AD when faced with a potential risk of rear-end collision.	61 Mild AD<br><br>115 Healthy Controls (HC)	NINCDS-ADRDA criteria for AD.	MMSE<br>Cogstat<br>JLO<br>ROCFT<br>AVLT-RECALL<br>WAIS-R (Blocks Design subtest)<br>BVRTT<br>TMT-B<br>COWA<br>UFOV<br>NVA<br>FVA<br>CS<br>SFM	**Driving Simulator test**:<br><br>The following measures were assessed:<br>First Reaction Time;<br>Occurrence of Improper Response (crashing, swerving out of lane or stopping abruptly and permanently).	Scores on the COGSTAT, CFT Copy and Recall, JLO, BVRT, TMT B, FVA, CS, and UFOV were significantly correlated with an increased risk of improper response. These tests correspond to domains of visual perception, attention, memory, visuospatial abilities, and executive functions.
Uc EY, Rizzo M, Anderson SW et al. (2005)	To assess driving behaviour in AD drivers during a landmark and traffic sign identification task.	33 Mild Probable AD<br><br>137 Healthy Controls (HC)	NINCDS-ADRDA criteria for AD.	MMSE<br>Cogstat<br>JLO<br>ROCFT<br>AVLT-RECALL<br>WAIS-R Blocks Design subtest<br>BVRTT<br>TMT-B<br>COWA<br>UFOV<br>NVA<br>FVA<br>CS<br>SFM	**Standardised road test using ARGOS instrumented vehicle.**<br><br>Divided in two parts: 'On-task' segment to assess landmark and traffic sign identification tasks (LTIT); and the 'no-task' segment typical of a standard road test.	Cognitive scores related to verbal (AVLT-RECALL) and nonverbal memory (CFT-RECALL and BVRT), executive function (TMT-B and COWA), visual perception (FVA, NVA, CS), visual attention (UFOVTOT), visuoconstructional abilities (CFT-COPY, BLOCKS, JLO), and overall cognitive function (COGSTAT) were significantly associated with LTIT outcome measures.<br><br>TMT-B, AVLT-Recall, JLO and CS were

© 2022. Camilleri L & Whitehead D. Published online at http://www.aginganddisease.org/EN/10.14336/AD.2022.1126

						significant predictors of LTIT percentage identification.<br><br>AVLT-Recall and SFM were predictors of at-fault safety errors.
Vaux LM, Ni R, Rizzo M et.al. (2010)	To assess the ability of AD and PD patients to detect impending collisions.	6 Mild AD<br><br>(Mean MMSE 24.5)<br><br>8 PD<br>18 Healthy Controls (HC)	NINCDS-ADRDA criteria for AD.<br><br>Diagnostic criteria described by Gelb DJ et.al. (1999) for PD.<br><br>MMSE for severity rating.	MMSE<br>Cogstat<br>JLO<br>ROCFT (CFT copy and recall versions)<br>AVLT-RECALL<br>WAIS-R (Blocks Design)<br>BVRTT<br>TMT-B<br>COWA<br>UFOV<br>NVA<br>FVA<br>CS	**Self-Reported Driving Behaviour**: Number of miles and days driven per week, number of accidents in the past two years and number of times pulled over in the past two years.<br><br>**Collision Detection task**.	**Cognitive and vision measures:** AD participants performed worse in all vision measures, MMSE and Cogstat when compared to HC participants. Worse Cogstat and UFOV scores were significantly correlated with a reduced collision detection sensitivity.
Venkatesan U et.al. (2018)	To investigate the relationship between driving and visuospatial search and binding in drivers with mild AD dementia.	42 Mild AD (CDR 0.5-1)<br><br>37 Healthy Controls (HC)	NINCDS-ADRDA criteria for AD.<br><br>CDR for severity rating.	MMSE<br>CDR<br>TMT-A<br>TMT-B<br>CDT	**The Rhode Island Road Test (RIRT)**<br><br>**Naturalistic Driving**: The Composite Driving Assessment Scale (CDAS).<br><br>**Visual Search Task**: Luminance motion (L+M) and colour motion (C+M) targets were presented. The following measures were used:<br>1) Median reaction time<br>2) Mean hit rate;<br>3) Mean false alarm rate.	The following measures showed a significant relationship to driving performance in the AD group:<br>1) L+M Hit Rate with Speed Control;<br>3) C+M False Alarm Rate and RIRT + CDAS Total Errors;<br>4) C+M False Alarm Rate and Response to Traffic;<br>5) CDT scores and CDAS Total<br>Errors;<br>6) TMT-A and RIRT Total Errors and Driving Awareness;<br>7) TMT-B and RIRT Driving Awareness and CDAS Response to Traffic.
Wild K and Cottrell V (2003),	To investigate how impairment in awareness in AD drivers may affect driving behaviours.	15 Probable or Possible Mild AD (CDR 0.5-1)<br><br>15 Healthy Controls (HC)	NINCDS-ADRDA criteria for AD.<br><br>CDR for severity rating.	MMSE<br>CDR	**Standardised road test** Management of intersections,<br>Managing Lane changes,<br>Maintaining lane position,<br>Speed control,<br>Follows at safe distance,<br>Appropriate signalling,<br>Proper use of mirrors,<br>Responds to road conditions,<br>Responds to road signs,<br>Handles conversational distraction.<br><br>**Participant and Informant/ Caregiver Rating**:<br>1) Discrepancy Questionnaire.	**Discrepancy scores**: AD participants were more likely to underestimate their impairment in recent memory when compared to caregiver score and HC participants were more likely to overestimate their impairment. There was no significant difference between self and informant reporting in the other items of the Discrepancy questionnaire in either group.<br><br>**10 item scores**: AD drivers scored significantly worse in all 10 items compared to HCs on both road test and caregiver rating. There was no significant

© 2022. Camilleri L & Whitehead D. Published online at http://www.aginganddisease.org/EN/10.14336/AD.2022.1126

					2) Driving Safety Questionnaire.	difference in self rating between HC and AD.<br><br>**Conclusion**: AD drivers have a reduced awareness of cognitive and driving abilities. Caregiver rating was more accurate than self-rating in the AD group but failed to recognise two important driving impairments.
Yamin S, Stinchcombe A, Gagnon S (2016)	To assess whether measures of attention, visual processing and global cognition are good predictors of driving performance in AD drivers.	20 Probable Mild AD (GDR stage 3)<br><br>21 Healthy Controls (HC)	NINCDS-ADRDA criteria for AD.<br><br>GDR scale for severity rating.	MMSE<br>MDR Scale (DRS2 and DRS2 Alternate)<br>TEA<br>UFOV<br>VOSP BNT	**Driving Simulator Test**:<br>The following errors were recorded:<br>Center line crossings<br>Road edge excursions,<br>Failure to stop at a stop sign or red light,<br>Speeding,<br>Illegal turns,<br>Off-road crashes,<br>Vehicle collisions.	The correlation between global cognitive ratings and rater score, total errors, and number of crashes was not statistically significant.<br><br>As a result, measures of global cognitive functions are non-specific and should not be used in isolation to predict fitness to drive in dementia. On the other hand, VOSP-object scores and TEA can give important information on driving safety in drivers with AD dementia.

Supplementary Table 4. Summary of Meta-Analysis on driving behaviour and assessment in Dementia

Author and year of publication	Study Aims	Number and type of studies included	Population Characteristics	Neurophysiological Assessments used	Driving Assessments Used	Results and conclusion
Chee JN, Rapoport MJ, Molnar F, Herrmann N, O'Neill D et.al. (2017)	To assess the correlation between on-road assessment and the risk motor vehicle crashes and/or driving impairment in drivers with dementia.	4 POS	Dementia of any severity and aetiology. Dementia diagnosed by established criteria or physician referral.<br><br>240 Dementia<br><br>151 Healthy Controls	CDR	On road driving assessment	There was a higher failure rate among dementia drivers when compared to healthy controls after performing an on-road driving assessment.<br><br>Two of these studies examined failure rate in dementia patients with CDR 0.5 and CDR 1.0 separately. The absolute increase in test failure risk was 11-12% in CDR 0.5 compared to 18-22% in CDR 1.0 patients.

© 2022. Camilleri L & Whitehead D. Published online at http://www.aginganddisease.org/EN/10.14336/AD.2022.1126

SUPPLEMENTARY DATA

Hird MA, Egeto P, Fischer CE et.al. (2016)	To identify driving assessment methods used in drivers with MCI and AD dementia.	32 POS	1,293 AD Dementia<br><br>92 MCI<br><br>2,040 Healthy older controls (HC)	Maze test,<br>TMT-A,<br>TMT-B,<br>Verbal Fluency,<br>UFOV,<br>BVRT,<br>HVLT,<br>AVLT,<br>Finger Tapping,<br>ROCF,<br>CS,<br>SFM,<br>MMSE.<br><br>Each test was categorized into either of the following cognitive domains: executive functions, attention, visual memory, verbal memory, visuospatial function, vision, psychomotor,<br>global cognition.	On-road assessment in 19 studies;<br><br>Non-road simulator driving assessments in 9 studies;<br><br>Caregiver reporting in 3 studies;<br><br>Crash records in 1 study.	Maze Test, TMT-A, TMT-B, verbal fluency, UFOV, SFM, CS, Finger Tapping, BVRT, ROCF-Copy and MMSE were significantly predicted driving outcome. Maze Test, TMT-A and TMT-B were the best predictors of driving outcomes.<br><br>Executive function, attention, visuospatial function, global cognition, visual memory and vision were significant predictors of driving outcome.<br><br>The above correlation was observed across all driving outcomes.<br><br>A higher CDR rating indicating more severe disease, was significantly associated with a higher marginal and fail rate on on-road and simulator driving assessments.

© 2022. Camilleri L & Whitehead D. Published online at http://www.aginganddisease.org/EN/10.14336/AD.2022.1126

Reger MA, Welsh RK, Watson GS et.al. (2004)	To assess the relationship between neurophysiological functioning and driving performance in drivers with dementia.	27 POS	2037 participants with AD dementia.	1) **Mental Status and General Cognition**: MMSE, DRS, Blessed Dementia Rating Scale, Mattis Dementia Rating Scale, Temporal Orientation, Behavior Rating Scale, CDR, Direct Assessment of Functional Status, Full Scale IQ, IADL Scale, Shipley IQ Estimate, Sum of Boxes, Expanded Constructional Praxis.<br><br>2) **Attention and Concentration**: Trails A, Digit Span, Reaction Time, UFOV, Digit Symbol, Attention Switching, CPT, Crossing-Off, Freed's Selective Attention, Letter Cancellation, Mackworth Clock, Mattis Attention, Sternberg Test, Visual Search & Attention Test, Visual Tracking, WORLD spelled backwards.<br><br>3) **Visuospatial Skills**: BD, Picture Completion, Benton Copy, Benton Line Orientation, CDT, Figure Ground Test, HVOT, Mattis Construction, Visuospatial task–Stanford–Binet Intelligence Scale.<br><br>4) **Memory**: Logic Memory, BVRT, Visual Reproduction, Facial Recognition Test, Associate Learning, Mattis Memory, Spatial Recognition Test, Word List Learning.<br><br>5) **Executive Function**: TMT-B, Word Fluency, Stroop Colour–, Picture Arrangement, Category Fluency and Naming, Mattis Initiation/Perseveration, Mazes, Shipley Abstraction.<br><br>6**) Language**: BNT, Information, Verbal IQ, Aphasia Battery, Shipley Vocabulary, Reading IQ Equivalent.	On-road assessment in 12 studies;<br><br>Non-road driving assessments (e.g., driving simulators and tests of driving knowledge) in 9 studies;<br><br>Caregiver reporting in 8 studies.	The following were the statistically significant correlations between cognitive domains and driving measures without control participants:<br><br>- Moderate correlation between Visuospatial cognition non-road tests;<br><br>- Nearly moderate correlation between Visuospatial cognition and on-road tests;<br><br>- Low correlation between visuospatial cognition and caregiver rating;<br><br>- Nearly moderate correlation between Mental status and non-road test;<br><br>- Low correlation between Mental status and caregiver rating;<br><br>- Small correlation between Attention and on-road tests.

© 2022. Camilleri L & Whitehead D. Published online at http://www.aginganddisease.org/EN/10.14336/AD.2022.1126

Rashid R et.al. (2020)	To Identify the strength of association between office based cognitive tests and on-road driving assessment in drivers with dementia.	16 POS	Dementia of any severity and aetiology. Dementia diagnosed by established criteria or physician referral.<br><br>673 Dementia:<br>457 AD,<br>19 MCI,<br>114 Not specified,<br>45 VaD,<br>13 FTD,<br>10 DLB,<br>15 Mixed dementia.	1) **Mental and cognitive status**: MOCA, Cogstat, Sort Blessed Test, MMSE, CDR.<br><br>2) **Attention and Concentration**: UFOV, TMT-A, AMIPB Information processing, Stroke Driver Screening Assessment, NAB, Hazard Perception Test, Es and Fs, Digit Symbol, Measure of Inhibition, CDR Orientation, Visual Orientation, Two Mazes, Digit Span Forward, Attention Switching, Grooved Peg Board, Choice Reaction Time, Balloon Test, Letter Cancellation Test, Zazzo's Cancellation Test.<br><br>3**) Visuospatial Skills**: RFC, WAIS Block Design, VOSP Cube Analysis, Benton Copy, Drawings, Motor Free Visual Perception Test, Figure Ground Test, VOSP Position Discrimination, VOSP Incomplete Letters, JLO, Money Road Map Test, Visual Form Discrimination, Picture Completion, Visual Discrimination Stimuli, NAB..<br><br>4) **Memory**: Ref CFT Figure Recall, Figural Memory Test, Benton VRT, Brief Visual Memory Test, SORT, HVLT Learning, Wechsler memory Scale, AVLT, DST Backwards.<br><br>5) **Executive Function**: TMT-B, Wisconsin Card Sorting Test, SDSA Road Sign Recognition, SDSA, Stroop Test, BADS Key Search, OT-DHMT, BADS Rule Shift Card Action Programme, Shipley Institute of Living Scale.<br><br>6) **Language**: Aphasia Battery, Modified Token Test, COWA, Semantic Fluency Test, BNT, Shipley Institute of Living Scale.	On road driving assessment.	All cognitive domains except language, showed a moderate significant correlation with on-road performance.<br><br>Language showed a moderate but non-significant correlation.<br><br>As a result, measures of memory, attention, visuospatial skills and executive function, are associated with on-road driving performance in drivers with dementia.

Supplmenetary Table 5. Quality Appraisal and Risk of Bias of Observational Studies.
(*Red = High risk, Green = Low risk, Yellow = Uncertain level of risk due to insufficient information)

Author and year of publication	Study Designs	Newcastle-Ottawa Scale Score	Risk of Bias *<br>(RoBANS/ ROBINS-1 Tool)	CEBM

© 2022. Camilleri L & Whitehead D. Published online at http://www.aginganddisease.org/EN/10.14336/AD.2022.1126

SUPPLEMENTARY DATA

		Selection	Comparability	Exposure/ Outcome	Total Score	Selection Bias	Confounding Bias	Misclassification Bias	Performance Bias	Blinding/ Detection Bias	Attrition Bias	Reporting Bias	
Aksan N, Anderson SW, Dawson J et.al. (2014),	Case-Control	3	1	1	5								3b
Barrash J, Stillman A, Anderson SW et.al. (2010)	Cross-sectional Cohort	3	1	2	6								1b
Bixby K, Davis JD, Ott BR (2015),	Cross-sectional	3	1	2	6								2c
Brown LB, Stern RA, Cahn-Weiner DA, Rogers B, Messer MA et.al. (2005)	Case-Control	3	1	2	6								3b
Brown LB, Ott BR, Papandonatos GD et.al. (2005)	Case-Control	3	1	2	6								3b
Burns T, Lawler K, Lawler D et.al. (2018)	Cross-sectional	2	0	2	4								4
Crivelli L, Russo MJ, Farez MF, Bonetto M, Prado C et.al. (2019)	Cross-sectional Cohort	3	1	2	6								2b
Croston J, Meuser TM, Berg-Weger M et.al. (2009)	Cross-sectional	1	0	2	3								4
Davis JD, Papandonates GD, Miller LA et.al. (2012)	Cross-sectional Cohort	3	1	3	7								1b
Davis JD, Wang S, Festa EK et.al. (2018)	Cross-sectional Cohort	2	1	3	6								1b
Davis JD, Babulal GM, Papandonatos GD et.al. (2020)	Cross-sectional cohort	3	0	2	5								2b
Dawson JD, Anderson SW, Uc EY et.al. (2009)	Case Control	3	1	3	7								3b
de Simone V, Kaplan L, Patronas N et.al. (2007)	Cross-sectional Cohort	2	1	3	6								2b
Duchek JM, Carr DB, Hunt L et.al. (2003)	Longitudinal prospective	2	1	2	5								2c
Economou A, Pavlou D, Beratis I, et al. (2020)	Case-Control	3	1	2	6								3b
Fernandez R and Duffy CJ (2012)	Case-Control	2	1	1	4								4
Fuermaier ABM, Piersma D, de Waard D, Davidse RJ, de Groot J et.al. (2019)	Cross-sectional Cohort	2	0	3	5								1b
Fujito R, Kamimura N, Ikeda M et.al. (2016)	Cohort	2	1	2	5								2b
Frittelli C, Borghetti D, Ludice G et al (2009)	Cross-sectional Case Control	2	1	2	5								3b
Grace J, Amick M, D'Abreu A, et.al. (2005)	Cross-sectional Case Control	3	2	2	7								3b

© 2022. Camilleri L & Whitehead D. Published online at http://www.aginganddisease.org/EN/10.14336/AD.2022.1126

SUPPLEMENTARY DATA

Lafont S, Marin-Lamellet C, Paire-Ficout L et.al. (2010)	Case-Control	3	1	2	6								3b
Lovell RK and Russell KJ (2005)	Cross-sectional Cohort	2	0	1	3								4
Luzzi S, Cafazzo V, Damora A, Fabi K, Fringuelli FM et. al. (2015)	Cohort	2	2	2	6								1b
Manning KJ et.al. (2014)	Cross-sectional Cohort	3	1	2	6								1b
O'Brien HL, Tetewsky SJ, Avery LM et.al. (2001)	Cross-sectional Case Control	2	1	2	5								3b
Ott B.R, Heindel WC, Whelihan WM et.al. (2000)	Cross-sectional Case Control	3	1	1	5								3b
Ott BR, Heindel WC, Whelihan WM et.al. (2003)	Cross-sectional Case Control	2	0	1	3								4
Ott BR, Anthony D, Papandonatos GD et.al. (2005)	Case-Control	2	1	2	5								3b
Ott BR, Festa EK, Amick MM et.al. (2008) [1]	Cohort	3	1	2	6								1b
Ott BR, Heindel WC, Papandonatos GD et.al. (2008) [2]	Prospective Longitudinal	3	1	3	7								2c
Ott BR et.al. (2012)	Cross-sectional Case Control	3	1	2	6								3b
Paire-Ficout L, Lafont S, Conte F, Coquillant A, Fabrigoule C et.al. (2018)	Cohort	3	1	2	6								1b
Piersma D, Fuermaier ABM, De Waard D et.al. (2016)	Cross-sectional Case Control	3	1	3	7								3b
Piersma D, Fuermaier ABM, De Waard D et.al. (2018)	Cross-sectional Case Control	2	1	3	6								3b
Rizzo M et.al. (2001)	Cohort	3	1	2	6								1b
Stinchcombe A et.al. (2016)	Cohort	3	1	3	7								1b
Tomioka H, Yamagata B, Takahashi T et.al. (2009)	Cohort	2	1	2	5								2b
Uc EY, Rizzo M, Anderson SW et.al. (2004)	Cross-sectional Case Control	4	1	3	8								3b
Uc EY, Rizzo M, Anderson SW et al. (2005)	Cross-sectional Case Control	4	1	3	8								3b
Uc EY, Rizzo M, Anderson SW et.al. (2006)	Cross-sectional Case Control	3	1	3	7								3b
Vaux LM, Ni R, Rizzo M et.al. (2010)	Cohort	3	1	1	5								2b

© 2022. Camilleri L & Whitehead D. Published online at http://www.aginganddisease.org/EN/10.14336/AD.2022.1126

Venkatesan U et.al. (2018)	Cross-sectional	3	1	2	6								2c
Wild K and Cottrell V (2003)	Case-Control	3	1	2	6								3b
Yamin S, Stinchcombe A, Gagnon S (2016)	Cross-sectional Case Control	3	2	2	7								3b

Supplementary Table 6. Quality Appraisal of Meta-analysis.

**Author and year of publication**	**Heterogeneity (I^2)**	**Risk of Publication Bias**	**AMSTAR-2 Score (negative score items)**
Chee JN, Rapoport MJ, Molnar F, Herrmann N, O'Neill D et.al. (2017)	LOW I^2 = 0% (*p*=0.68)	Forest Plot showed no asymmetry indicative of low risk of bias, however few articles to make results reliable.	Low quality (Item 7)
Reger MA, Welsh RK, Watson GS et.al. (2004)	Moderate to High.<br><br>Q statistics varied across cognitive domains, driving outcomes and the presence/ absence of controls.<br><br>Statistical significance reached in following Q values:<br>- All combined neurophysiological tests to all three driving measures when controls present;<br>- Mental Status domain with care giver report without controls.	Not analysed	Low quality<br><br>(Items 5, 6, 9, 10, 12, 15, 16)
Hird MA, Egeto P, Fischer CE et.al. (2016)	Low to High I^2<br><br>Varied across cognitive tests and domains:<br><br>0% (Finger Tapping) to 88% (TMT-A);<br>28% (vision) to 89% (psychomotor domain).	Trim-and-fill method and Egger's test showed the presence of publication bias in all cognitive domains except visual memory, psychomotor and global cognition.	High quality
Rashid R et.al. (2020)	Low to High I^2 varied across cognitive domains:<br><br>Attention 33.29%<br>Memory 43.97%<br>Executive Function 45.32%<br>Attention 59.42%<br>Mental and Cognitive status 68.47%<br>Language 85.18%	No asymmetry on Funnel Plot and Egger test indicating low risk of bias but there were less than 10 studies for each cognitive domain so results are unreliable.	Moderate quality<br><br>(Items 10 and partial 7)

Appendix

A. List of excluded articles in literature search

1. Full text not found (n=6)

- Russell KJ, Unsworth C, Lovell R, Woodward M, Browne M, 'A randomized controlled trial to determine the effect of assessment location and number of assessments on driving performance of people with dementia' Alzheimer's & dementia, 2017, 13(7), P899

- GRAY-VICKREY PEG (2010) 'Research Updates: Driving and dementia', Alzheimer's Care Today; 11(3): 149-150 doi: 10.1097/ACQ.0b013e3181ec008a

© 2022. Camilleri L & Whitehead D. Published online at http://www.aginganddisease.org/EN/10.14336/AD.2022.1126

SUPPLEMENTARY DATA

- Frank-Garcia, A., Zea Sevilla, M.A., Barbas, R.G., Soler, M.V., Perdices, N.J. and Barranco, A.T. (2009), P3-064: Could driving changes be used as a tool for predicting development of dementia? Alzheimer's & Dementia, 5: P360-P360. https://doi.org/10.1016/j.jalz.2009.04.1140
- Freeman-Costin K, Holland P, Tappan R, ‘Methodologic considerations in prolonging safe driving in mild Alzheimer's disease’ Journal of the American geriatrics society, 2018, 66(6). https://doi.org/10.1111/jgs.15376
- Fernandez-Romero R, Cox DJ. Impaired driving capacity in early-stage Alzheimer’s is associated with decreased cortical responsiveness to simulated self-movement. Alzheimers Dement. (2016) 12:882. doi: 10.1016/j.jalz.2016.06.1824
- Badenes Guia D, Casas Hernanz L, Aguilar Barbera M, Cejudo Bolivar JC. Assessment of dementia of car driving abilities in patients with mild cognitive impairment and dementia. Mapfre Medicina 2007;18(2):98-107.

2. <u>Not published in the English language (n= 6)</u>

- Arai Y, Arai A, Misuno Y. Automobile driving by dementia patients: a need for social support. Seishin Shinkeigaku Zasshi 2009;111(1):101-7
- Badenes Guia D, Casas Hernanz L, Cejudo Bolivar JC, Aguilar Barberà M. Evaluation of the capacity to drive in patients diagnosed of mild cognitive impairment and dementia. Neurologia 2008;23(9):575-82
- Ernst, J., Krapp, S., Schuster, T., Förstl, H., Kurz, A., & Diehl-Schmid, J. (2010). [Car driving ability of patients with frontotemporal lobar degeneration and Alzheimer's disease]. Der Nervenarzt, 81(1), 79-85.
- Kamimura N. Dementia and automobile driving. Seishin Shinkeigaku Zasshi. 2009;111(8):960-6
- Lukas A, Nikolaus T. Driving ability and dementia. Zeitschri4 für Gerontologie und Geriatrie 2009;42(3):205-11.
- Paccalin M, Bouche G, Barc-Pain S, Merlet-Chicoine I, Nedelec C, Gil R. Automobile driving among patients with dementia. Survey in the Poitou-Charentes region. Presse Med. 2005; Jul 23,24:919-22.

3. <u>Study design does not meet inclusion criteria or meets exclusion criteria</u> (n= 149)

- Adler D (2010), ‘Driving decision-making in older adults with dementia’ SAGE journals; 9(1): 45-60. https://doi.org/10.1177/1471301209350289
- Adler G (2007), ‘Intervention Approaches to Driving and Dementia’. Health & Social Work; Oxford 32(1): 75-9. DOI:10.1093/hsw/32.1.75
- Adler G and Rottunda SJ (2011), ‘The Driver with Dementia: A Survey of Physician Attitudes, Knowledge, and Practice’ American Journal of Alzheimer’s Disease & Other Dementias; 26(1) 58-64. DOI: 10.1177/1533317510390350
- Adler G, Kuskowski M. Driving cessation in older men with dementia. Alzheimer Dis Assoc Disord 2003;17: 68 – 71.
- Adler G, Silverstein NM. At-risk drivers with Alzheimer's disease: recognition, response, and referral. Traffic Inj Prev. 2008 Aug;9(4):299-303. doi: 10.1080/15389580801895186.
- Adler G, Susan Rottunda, Maurice Dysken (2005), ‘The older driver with dementia: An updated literature review’ Journal of Safety Research; 36(4): 399-407. https://doi.org/10.1016/j.jsr.2005.07.005.
- Allan CL, Behrman S, Baruch N, et al. Driving and dementia: a clinical update for mental health professionals’ Evidence-Based Mental Health 2016;19:110-113.

SUPPLEMENTARY DATA

- Alzheimer's Australia (2010) Driving and dementia in New South Wales: Discussion Paper One. Retrieved from http://www.alzheimers.org.au on 30 April 2010.
- American Medical Association, U.S. Department of Transportation, and National Highway Traffic Safety Administration. Physician's guide to assessing and counseling older drivers. Washington (DC): National Highway Traffic Safety Administration, 2003; http://www.nhtsa.dot.gov/people/injury/olddrive/OlderDriversBook/pages/I.
- Andrew C., Carmody J., Lewis K., Traynor V. & Iverson D. (2013) Developing a decision aid for drivers living with dementia: supporting consumers and carers in the process of making decisions about driving retirement. Australian Occupational Therapy Journal 60(S1), 70–71. Australian Bureau of Statistics (2008) 3222.0 Population Projections, Australia, 2006 to 2101. Australian Government, Canberra. Retrieved from http://www.abs.gov.au/ausstats/abs@.nsf/mf/3222.0 on 7 November 2008.
- Andrew, C., Traynor, V. & Iverson, D. (2015) An integrative review: understanding driving retirement decisions for individuals living with a dementia. Journal of Advanced Nursing 71(12), 2728– 2740. doi: 10.1111/jan.12727
- Anstey KJ, Joanne Wood, Stephen Lord, Janine G. Walker (2005), 'Cognitive, sensory, and physical factors enabling driving safety in older adults' Clinical Psychology Review; 25(1): 45-65. https://doi.org/10.1016/j.cpr.2004.07.008
- Apolinario D, Magaldi RM, Busse AL, de Costa Lopez L, Tisson Kasai JY, Satomi E (2009), 'Cognitive impairment and driving: A review of the literature' Dementia & Neuropsychologia; 3(4): 283-290. DOI: https://doi.org/10.1590/S1980-57642009DN30400004.
- Australian and New Zealand Society for Geriatric Medicine. Australian and New Zealand Society for Geriatric Medicine position statement driving and dementia. Australas Journal of Ageing 2010;29(3):137-41
- Babulal GM, Kolady R, Stout SH, Roe CM. A Systematic Review Examining Associations between Cardiovascular Conditions and Driving Outcomes among Older Drivers. Geriatrics. 2020; 5(2):27. https://doi.org/10.3390/geriatrics5020027
- Barbas NR, Wilde EA. Competency issues in dementia: medical decision making, driving, and independent living. J Geriatr Psychiatry Neurol. 2001 Winter;14(4):199-212. doi: 10.1177/089198870101400405.
- Barco PP, Wallendorf M, Blenden G, Rutkoski K, Dolan K, Carr D (2021) 'Caregiver Prediction of Driving Fitness in Older Adults with Dementia' Clinical Gerontologist. DOI: 10.1080/07317115.2021.1872130.
- Bédard M, Weaver B, Man-Son-Hing M, Classen S, Porter M; CanDRIVE investigators. The SIMARD Screening Tool to Identify Unfit Drivers: Are We There Now? J Prim Care Community Health. 2011 Apr;2(2):133-5. doi: 10.1177/2150131910397704. PMID: 23804747.
- Bennet JM, Chekaluk E, Batchelor J (2016), 'Cognitive Tests and Determining Fitness to Drive in Dementia: A Systematic Review' J Am Geriatr Soc 64: 1904- 1917. https://doi.org/10.1111/jgs.14180
- Bennet JM, Chekaluk E, Batchelor J (2019), 'Determining Fitness to Drive for Drivers with Dementia: A Medical Practitioner Perspective' Journal of the Australasian College of Road Safety; 30(2): 9-17.
- Beran RG. Analysis and overview of the guidelines for assessing fitness to drive for commercial and private vehicle drivers. Intern Med J. 2005 Jun;35(6):364-8. doi: 10.1111/j.1445-5994.2005.00813.x. PMID: 15892768.
- Bernstein JPK, Matthew Calamia, Molly Z. Meth & Daniel Tranel (2019) Recommendations for Driving After Neuropsychological Assessment: A Survey of Neuropsychologists, The Clinical Neuropsychologist, 33:6, 971-987, DOI: 10.1080/13854046.2018.1518490
- Breen DA, Breen DP, Moore JW, Breen PA, O'Neill D. Driving, and dementia. BMJ. 2007 Jun 30;334(7608):1365-9. doi: 10.1136/bmj.39233.585208.55.
- Brown LB, Ott BR. Driving, and dementia: a review of the literature. J Geriatr Psychiatry Neurol. 2004 Dec;17(4):232-40. doi: 10.1177/0891988704269825.

© 2022. Camilleri L & Whitehead D. Published online at http://www.aginganddisease.org/EN/10.14336/AD.2022.1126

SUPPLEMENTARY DATA

- Burke AD, Yaari R, Tariot PN, Hall GR, Dougherty J, Brand H, Fleisher AS (2013), ‘The threat of behavioral changes in dementia’ Prim Care Companion CNS Disord.; 15(1): PCC.13alz01507. doi: 10.4088/PCC.13alz01507
- Byszewski, A., Aminzadeh, F., Robinson, K. et al. When it is time to hang up the keys: the driving and dementia toolkit – for persons with dementia (PWD) and caregivers – a practical resource. BMC Geriatr 13, 117 (2013). https://doi.org/10.1186/1471-2318-13-117
- Byszewski, A.M., Graham, I.D., Amos, S., Man-Son-Hing, M., Dalziel, W.B., Marshall, S., Hunt, L., Bush, C. and Guzman, D. (2003), A Continuing Medical Education Initiative for Canadian Primary Care Physicians: The Driving and Dementia Toolkit: A Pre- and Post-evaluation of Knowledge, Confidence Gained, and Satisfaction. Journal of the American Geriatrics Society, 51(10): 1484-1489. https://doi.org/10.1046/j.1532-5415.2003.51483.x
- Cable, G., Reisner, M., Gerges, S. and Thirumavalavan, V. (2000), Knowledge, Attitudes, and Practices of Geriatricians Regarding Patients with Dementia Who Are Potentially Dangerous Automobile Drivers: A National Survey. Journal of the American Geriatrics Society, 48: 14-17. https://doi.org/10.1111/j.1532-5415.2000.tb03022.x
- Cameron D, Zucchero Sarracini C, Rozmovits L, Naglie G, Herrmann N, Molnar F, Rapoport M (2017). Development of a decision-making tool for reporting drivers with mild dementia and mild cognitive impairment to transportation administrators. International Psychogeriatrics, 29(9), 1551-1563. doi:10.1017/S1041610217000242.
- Carmody J, Traynor V, Iverson D, Marchetti E. Driving, dementia, and Australian physicians: primum non nocere? Intern Med J. 2013 Jun;43(6):625-30. doi: 10.1111/imj.12161.
- Carmody, J., Granger, J., Lewis, K., Traynor, V., & Iverson, D. (2013). What factors delay driving retirement by individuals with dementia? (The doctors’ perspectives). Journal of the Australasian College of Road Safety, 24(1), 10–16. https://search.informit.org/doi/10.3316/informit.185847811344163
- Carmody, J., Traynor, V. and Iverson, D. (2012) ‘Dementia and driving: An approach for general practice’, Australian Family Physician. Royal Australian College of General Practitioners, 41(4), pp. 230–233. Accessed from https://search.informit.org/doi/10.3316/informit.157997205503288.
- Carr DB, Duchek JM, Meuser T et al. Older drivers with cognitive impairment. Am Fam Physician 2006; 73:1035–1036
- Carr DB, O'Neill D. Mobility, and safety issues in drivers with dementia. Int Psychogeriatr. 2015 Oct;27(10):1613-22. doi: 10.1017/S104161021500085X.
- Carr DB, Ott BR. The Older Adult Driver with Cognitive Impairment: “It's a Very Frustrating Life”. JAMA. 2010;303(16):1632–1641. doi:10.1001/jama.2010.481
- Carter, K., Monaghan, S., O'Brien, J., Teodorczuk, A., Mosimann, U., Taylor, J.-P. (2015), Driving and dementia: a clinical decision pathway, Int J Geriatr Psychiatry, 30, pages 210– 216, doi: 10.1002/gps.4132
- Centers for Disease Control and Prevention. (2015). Older adult drivers. Injury prevention and control: Motor vehicle safety. Retrieved from http://www.cdc.gov/Motorvehiclesafety/Older_Adult_Drivers/index.html.
- Clare L, Marková I, Verhey F, Kenny G. Awareness in dementia: A review of assessment methods and measures. Aging Ment Health. 2005 Sep;9(5):394-413. doi: 10.1080/13607860500142903.
- Clark M, Hecker J, Cleland E, Field C & Berndt A (2000) The effect of dementia on driving performance, Australian and New Zealand Journal of Psychiatry, 34:sup1, A13-A14, DOI: 10.1080/000486700573
- Clark M, Hecker J, Cleland E, Field C, Berndt A, Crotty M, Snellgrove C. Dementia and Driving. Australian Transport Safety Bureau. CANBERRA ACT 2608, Australia, 2005.
- Clift BJ (2015), ‘People with dementia in the driving seat: using a participatory approach to research in the development of a driver screening tool’ Accessed from http://usir.salford.ac.uk/id/eprint/35951/
- Davis RL, Ohman J (2017), ‘Driving in Early-Stage Alzheimer’s Disease: An Integrative Review of the Literature’ Res Gerontol Nurs. 2017; 10(2):86-100.

SUPPLEMENTARY DATA

- Desapriya E, Ranatunga Y and Pike I (2012), ‘We need evidence based tools to identify medically at risk drivers’ BMJ; 345:e7087. DOI:10.1136/bmj.e7087
- De Silva, Michael A. Gregory, Shree S. Venkateshan, Chris P. Verschoor, Ayse Kuspinar, "Examining the Association between Life-Space Mobility and Cognitive Function in Older Adults: A Systematic Review", Journal of Aging Research, vol. 2019, Article ID 3923574, 9 pages, 2019. https://doi.org/10.1155/2019/3923574
- Determining Medical Fitness to Operate Motor Vehicles: CMA’s Driver’sGuide, 7th Ed. Ottawa, Canada: Canadian Medical Association, 2006.56.
- Dickerson AE & Michel Bédard (2014) Decision Tool for Clients with Medical Issues: A Framework for Identifying Driving Risk and Potential to Return to Driving, Occupational Therapy In Health Care, 28:2, 194-202, DOI: 10.3109/07380577.2014.903357
- Dickerson AE (2013), ‘Driving Assessment Tools Used by Driver Rehabilitation Specialists: Survey of Use and Implications for Practice.’ Am J Occup Ther; 67(5):564–573. https://doi.org/10.5014/ajot.2013.007823
- Dickerson AE (2014) ‘Driving with Dementia: Evaluation, Referral, and Resources’ Occupational Therapy In Health Care; 28(1): 62-76. DOI: 10.3109/07380577.2013.867091
- Dickerson AE (2014) Screening and Assessment Tools for Determining Fitness to Drive: A Review of the Literature for the Pathways Project, Occupational Therapy In Health Care, 28:2, 82-121, DOI: 10.3109/07380577.2014.904535
- Dobbs B, Zirk H, Daly S (http://www.stacommunications.com/customcomm/Back-issue_pages/AD_Review/adPDFs/2009/February2009/13.pdf
- Dobbs BM, Carr DB, Morris JC (2002), ‘Evaluation and Management of the Driver with Dementia’ The Neurologist; 8(2): 61-70. Accessed from https://journals.lww.com/theneurologist/Abstract/2002/03000/Evaluation_and_Management_of_the_Driver_with.1.aspx
- Dubinsky RM, Stein AC, Lyons K. Practice parameter: risk of driving and Alzheimer's disease (an evidence-based review): report of the quality standards subcommittee of the American Academy of Neurology. Neurology. 2000 Jun 27;54(12):2205-11. doi: 10.1212/wnl.54.12.2205.
- Eby DW, Molnar LJ (2010), ‘Driving Fitness and Cognitive Impairment’ JAMA; 303(16):1642-1643. doi:10.1001/jama.2010.495
- Ferrah N, Obieta A, Ibrahim JE, et al ‘Inequity in health: older rural driving and dementia’ Injury Prevention 2016;22:292-296. http://dx.doi.org/10.1136/injuryprev-2015-041601.
- Fisher, Mark, Thomson, Sue. (2014) Dementia and Driving Safety: A clinical guideline. Version 3. This document is available electronically at www.healthpoint.co.nz; http://www.healthnavigator.org.nz/>
- Frank CC, Lee L, Molnar F (2018), ‘Driving assessment for people with dementia’ Canadian Family Physicians, 64 (10) 744.
- Freund B, Gravenstein S, Ferris R, Shaheen E. Evaluating driving performance of cognitively impaired and healthy older adults: a pilot study comparing on-road testing and driving simulation. J Am Geriatr Soc. 2002;50(7):1309-10.
- Fujito, R., Kamimura, N., Ikeda, M., Koyama, A., Shimodera, S., Morinobu, S., & Inoue, S. (2016). Comparing the driving behaviours of individuals with frontotemporal lobar degeneration and those with Alzheimer's disease. Psychogeriatrics, 16(1), 27-33.
- Fuller R. Towards a general theory of driver behaviour. Accident; Analysis and Prevention 2005;37(3):461-72.
- Handley JD, Thomas RH, McKenna P, et al ‘On the road again: assessing driving ability in patients with neurological conditions’ Practical Neurology 2017;17:203-206. http://dx.doi.org/10.1136/practneurol-2017-001601

© 2022. Camilleri L & Whitehead D. Published online at http://www.aginganddisease.org/EN/10.14336/AD.2022.1126

SUPPLEMENTARY DATA

- Hogan DB. Which older patients are competent to drive? Approaches to office-based assessment. Can Fam Physician. 2005 Mar;51(3):362-8.
- Hoggarth PA (2013), 'Diagnosis of cognitive impairment and the assessment of driving safety in New Zealand: a survey of Canterbury GPs' NZMJ; 126(1387): 87. URL: http://journal.nzma.org.nz/journal/126-1387/5946/
- Hoover L (2010), 'AAN Updates Guidelines on Evaluating Driving Risk in Patients with Dementia' Am Fam Physician.; 82(9):1144-1147. Available at: http://www.neurology.org/cgi/content/full/74/16/1316
- http://healthforce.co.nz/a/nzdc-connect/wp-content/uploads/2015/09/driving-pdf.pdf
- Inasu P https://www.gmjournal.co.uk/media/21603/september2009p526.pdf
- Iverson DJ, Gronseth GS, Reger MA, Classen S, Dubinsky RM, Rizzo M; Quality Standards Subcomittee of the American Academy of Neurology (2010), 'Practice parameter update: evaluation and management of driving risk in dementia: report of the Quality Standards Subcommittee of the American Academy of Neurology.' Neurology.; 74(16):1316-24. doi: 10.1212/WNL.0b013e3181da3b0f.
- Jacobs M, Hart EP, Roos RAC (2017), 'Driving with a neurodegenerative disorder: an overview of the current literature' J Neurol; 264:1678–1696. DOI 10.1007/s00415-017-8489-9.
- Jang, RW, Man-Son-Hing, M, Molnar, FJ, et al. Family physicians' attitudes and practices regarding assessments of medical fitness to drive in older persons. J Gen Intern Med 2007; 22(4): 531–543.
- Kakaiya R, Tisovec R, Fulkerson P. Evaluation of fitness to drive. The physician's role in assessing elderly or demented patients. Postgrad Med. 2000 Mar;107(3):229-36. doi: 10.3810/pgm.2000.03.953.
- Kay LG, Bundy AC, Cheal B (http://casr.adelaide.edu.au/rsr/RSR2011/1DPaper%20138%20Bundy.pdf
- Kennedy GJ (2009). Advanced age, dementia, and driving: Guidance for the patient, family and physician. Primary Psychiatry, 16(9), 19-23.
- Korner-Bitensky N, Bitensky J, Sofer S et al. Driving evaluation practices of clinicians working in the United States and Canada. Am J Occup Ther2006;60:428–434.57.
- Korner-Bitensky N, Gélinas I, Man-Son-Hing M & Marshall S (2005) 'Recommendations of the Canadian Consensus Conference on Driving Evaluation in Older Drivers' Physical & Occupational Therapy In Geriatrics; 23:2-3, 123-144, DOI: 10.1080/J148v23n02_08.
- Korner-Bitensky N, Jamie Bitensky, Susan Sofer, Malcolm Man-Son-Hing, Isabelle Gelinas; Driving Evaluation Practices of Clinicians Working in the United States and Canada. Am J Occup Ther 2006;60(4):428–434. https://doi.org/10.5014/ajot.60.4.428.
- Lafont S, Laumon B, Helmer C, Dartigues JF, Fabrigoule C. Driving cessation and self-reported car crashes in older drivers: the impact of cognitive impairment and dementia in a populationbased study. J Geriatr Psychiatry Neurol. 2008;21(3):171-82.
- Lee L, Molnar F. Driving and dementia. Efficient approach to driving safety concerns in family practice. Can Fam Physician 2017; 63:27-31. (Eng), e9–14 (Fr). Available from: www.cfp.ca/content/63/1/27.full.
- Lincoln N, Radford K, Devos H, Akinwuntan AE (2014), 'A shortened version of the Dementia Drivers' Screening Assessment' International Journal of Therapy and Rehabilitation 2014 21:6, 268-273. https://doi.org/10.12968/ijtr.2014.21.6.268
- Lloyd S, Cormack CN, Blais K, Messeri G, McCallum MA, Spicer K, Morgan S. Driving and dementia: a review of the literature. Can J Occup Ther. 2001 Jun;68(3):149-56. doi: 10.1177/000841740106800303.
- Lovas, Joel et al. 'Assessment and Reporting of Driving Fitness in Patients with Dementia in Clinical Practice: Data from SveDem, the Swedish Dementia Registry'. 1 Jan. 2016 : 631 – 638.

© 2022. Camilleri L & Whitehead D. Published online at http://www.aginganddisease.org/EN/10.14336/AD.2022.1126

SUPPLEMENTARY DATA

- Love CM. Cognitive impairment and dangerous driving: a decision-making model for the psychologist to balance confidentiality with safety. Dissertation Abstracts International: Section B: The Sciences and Engineering 2007;68(3-B):1933.
- Man-Son-Hing, M., Marshall, S.C., Molnar, F.J. and Wilson, K.G. (2007), Systematic Review of Driving Risk and the Efficacy of Compensatory Strategies in Persons with Dementia. Journal of the American Geriatrics Society, 55: 878-884. https://doi.org/10.1111/j.1532-5415.2007.01177.x
- Martin AJ, Marottoli R, O'Neill D. Driving assessment for maintaining mobility and safety in drivers with dementia. Cochrane Database Syst Rev. 2013 Aug 29;2013(8):CD006222. doi: 10.1002/14651858.CD006222.pub4.
- McCracken P, Caprio Tiscott JA, Dobbs AR (2001), 'Driving with dementia' The Canadian Alzheimer's disease Review [online]. Available from http://www.stacommunications.com/customcomm/Back-issue_pages/AD_Review/adPDFs/november2001/14.pdf. Accessed on 4th August 2021.
- McCracken PN 'The DrivaAble Assessment: A review' http://www.stacommunications.com/customcomm/back-issue_pages/ad_review/adpdfs/2007/may2007/04.pdf
- Meng A, Siren A, Teasdale TW (2013), 'Older drivers with cognitive impairment: Perceived changes in driving skills, driving-related discomfort and self-regulation of driving' European Geriatric Medicine; 4(3): 154-160. https://doi.org/10.1016/j.eurger.2013.01.002.
- Meuser TM, Carr DB, Ulfarsson GF. Motor-vehicle crash history and licensing outcomes for older drivers reported as medically impaired in Missouri. Accident; Analysis and Prevention 2009;41(2):246-52.
- Meuser TM, Carr DB, Unger EA, Ulfarsson GF. Family reports of medically impaired drivers in Missouri: Cognitive concerns and licensing outcomes. Accid Anal Prev. 2014;74C:17-23.
- Meuser TM, David B. Carr, MD, Marla Berg-Weger, PhD, Pat Niewoehner, OTR/L, CDRS, John C. Morris, MD, Driving and Dementia in Older Adults: Implementation and Evaluation of a Continuing Education Project, The Gerontologist, Volume 46, Issue 5, October 2006, Pages 680–687, https://doi.org/10.1093/geront/46.5.680
- Miller SM, Ruth E. Taylor-Piliae, Kathleen C. Insel (2016), 'The association of physical activity, cognitive processes and automobile driving ability in older adults: A review of the literature' Geriatric Nursing; 37(4): 313-320. https://doi.org/10.1016/j.gerinurse.2016.05.004
- Molnar FJ, Byszewski AM, Marshall SC, et al. In-office evaluation of medical fitness-to-drive: practical approaches for assessing older people. Can Fam Physician 2005; 51:372-9.
- Molnar FJ, Byszewski AM, Rapoport M, Dalziel WB (2009), 'Practical Experience-Based Approaches To Assessing Fitness to Drive in Dementia' Geriatrics and Ageing; 12(2): 83-92.
- Molnar FJ, Rapoport MJ, Roy M (2012), 'Driving and dementia: maximizing the utility of in-office screening and assessment tools.' Can Geriatr Soc J CME; 2(2):11-4. Available from: canadiangeriatrics.ca/2012/09/volume-2-issue-2-dementia-and-driving.
- Molnar, F.J., Patel, A., Marshall, S.C., Man-Son-Hing, M. and Wilson, K.G. (2006), Clinical Utility of Office-Based Cognitive Predictors of Fitness to Drive in Persons with Dementia: A Systematic Review. Journal of the American Geriatrics Society, 54: 1809-1824. https://doi.org/10.1111/j.1532-5415.2006.00967.x
- Moorhouse, P., Hamilton, L., Fisher, T., & Rockwood, K. (2011). Barriers to assessing fitness to drive in dementia in nova scotia: informing strategies for knowledge translation. Canadian geriatrics journal : CGJ, 14(3), 61–65. https://doi.org/10.5770/cgj.v14i3.7
- Moorhouse, P., & Hamilton, L. M. (2014). Not if, but when: impact of a driving and dementia awareness and education campaign for primary care physicians. Canadian geriatrics journal : CGJ, 17(2), 70–75. https://doi.org/10.5770/cgj.17.109
- Morgan E (2018), 'Driving Dilemmas: A Guide to Driving Assessment in Primary Care' Clinics in Geriatric Medicine; 34(1): 107-115. https://doi.org/10.1016/j.cger.2017.09.006.

© 2022. Camilleri L & Whitehead D. Published online at http://www.aginganddisease.org/EN/10.14336/AD.2022.1126

SUPPLEMENTARY DATA

- Mosimann UP, Bächli-Biétry J, Boll J, Bopp-Kistler I, Donati F, Kressig RW, et al. Consensus recommendations for the assessment of fitness to drive in cognitively impaired patients. Praxis 2011;101(7):437.
- Naidu, A., & McKeith, I. (2006). Driving, dementia and the Driver and Vehicle Licensing Agency: A survey of old age psychiatrists. Psychiatric Bulletin, 30(7), 265-268. doi:10.1192/pb.30.7.265
- Neilson D, Chacko E, Cheung G. Assessing driving fitness in dementia: a challenge for old age psychiatrists. Australas Psychiatry. 2019 Oct;27(5):501-505. doi: 10.1177/1039856219867020.
- Neitch, SM, Madero, G, Maynard, S. Driving Assessment Results in Patients with a Diagnosis of Dementia. West Virginia Medical Journal 107(3), pp.54-58, May/June, 2011
- Nichols, N., Fairholme, E., Thomas, M., & George, M. (2015). A Study of the Clinicians Approach to Patients' Driving Status in the Memory Clinic Setting. European Psychiatry, 30(S1), 1-1. doi:10.1016/S0924-9338(15)31118-4
- Nichols, N., Roberts, J., & George, M. (2014). EPA-1093 – Driving Assessment in Dementia – A Literature Review. European Psychiatry, 29(S1), 1-1. doi:10.1016/S0924-9338(14)78371-3
- Omer S, Dolan C, Dimitrov BD, Langan C, McCarthy G. General practitioners' opinions and attitudes towards medical assessment of fitness to drive of older adults in Ireland. Australas J Ageing. 2014;33(3):E33-6.
- Nuthall A, Anthony P. Road safety and the driver with dementia: shifting the debate up a gear. Nurs Older People. 2003 Mar;15(1):18-21. doi: 10.7748/nop2003.03.15.1.18.c2237. PMID: 12673998.
- Ott BR and Daiello LA (2010), 'How does dementia affect driving in older patients?' AGING HEALTH; 1(6). DOI: https://doi.org/10.2217/ahe.09.83.
- O'Neill D (2010), 'Deciding on driving cessation and transport planning in older drivers with dementia' European Geriatric Medicine; 1(1): 22-25. https://doi.org/10.1016/j.eurger.2010.01.001.
- Pastor DK, Jones A, Arms T. Where the Rubber Hits the Road: What Home Healthcare Professionals Need to Know About Driving Safety for Persons With Dementia. Home Healthc Now. 2017 Jan;35(1):26-32. doi: 10.1097/NHH.0000000000000482.
- Piersma D, de Waard D, Davidse R, Tucha O & Brouwer W (2016) Car drivers with dementia: Different complications due to different etiologies?, Traffic Injury Prevention, 17:1, 9-23, DOI: 10.1080/15389588.2015.1038786.
- Rapoport M, Yamin S, Vrkljan B et.al. (2019), 'PERSPECTIVES FROM THE FIELD: DESIGNING THE DRIVING CESSATION IN DEMENTIA INTERVENTION TOOLKIT (DCD-IT)' The American Journal of Geriatric Psychiatry; 27(3): S185-S186. https://doi.org/10.1016/j.jagp.2019.01.099.
- Rapoport M, Zucchero Sarracini C, Molnar F, Herrma N (2008), ' Driving with dementia: How to assess safety behind the wheel' Current Psychiatry; 7(12): 37-48
- Rapoport MJ, Chee JN, Carr DB, Molnar F, Naglie G et.al., An International Approach to Enhancing a National Guideline on Driving and Dementia. Curr Psychiatry Rep. 2018 Mar 12;20(3):16. doi: 10.1007/s11920-018-0879-x.
- Rapoport MJ, Herrmann N, Molnar FJ, Man-Son-Hing M, Marshall SC, Shulman K and Naglie G (2007), 'Sharing the responsibility for assessing the risk of the driver with dementia' CMAJ; 177 (6) 599-601; DOI: https://doi.org/10.1503/cmaj.070342
- Rapoport MJ, Sarracini CZ, Mulsant BM, Seitz DP, Molnar F, Naglie G, Herrmann N, Rozmovits L. A virtual second opinion: Acceptability of a computer-based decision tool to assess older drivers with dementia. Health Informatics J. 2020 Jun;26(2):911-924. doi: 10.1177/1460458219852870.
- Reime, N Pilgram, T Bertsche, Elderly drivers with dementia – how do experts and relatives assess their safety risk?, European Journal of Public Health, Volume 29, Issue Supplement_4, November 2019, ckz186.600, https://doi.org/10.1093/eurpub/ckz186.600.

© 2022. Camilleri L & Whitehead D. Published online at http://www.aginganddisease.org/EN/10.14336/AD.2022.1126

SUPPLEMENTARY DATA

- Selway JS (2018), ‘To Drive or Not to Drive: When There Is Dementia’ The Journal for Nurse Practitioners; 14(3): 202-209. https://doi.org/10.1016/j.nurpra.2017.12.033
- Sheridan, M. (2012). Assessing fitness to drive in dementia and other psychiatric conditions: A higher training learning opportunity at a driving assessment centre. The Psychiatrist, 36(3), 113-116. doi:10.1192/pb.bp.111.034983
- Siren A, Haustein S (2015), ‘Driving licences and medical screening in old age: Review of literature and European licensing policies’ Journal of Transport & Health; 2(1): 68-78. https://doi.org/10.1016/j.jth.2014.09.003
- Silva MT, Laks J, Engelhardt E (2009), ‘Neuropsychological tests and driving in dementia: a review of the recent literature. Rev Assoc Med Bras.; 55(4):484-8. doi: 10.1590/s0104-42302009000400027.
- Silverstein MN, Vanderbur M, ‘Community Mobility and Dementia: A Review of the Literature" (2006). Gerontology Institute Publications. Paper 34.http://scholarworks.umb.edu/gerontologyinstitute_pubs/34.
- Snyder, C.H. (2005), Dementia and Driving: Autonomy Versus Safety. Journal of the American Academy of Nurse Practitioners, 17: 393-402. https://doi.org/10.1111/j.1745-7599.2005.00070.x
- Toepper M and Falkenstein M (2019), ‘Driving Fitness in Different Forms of Dementia: An Update’ J Am Geriatr Soc.; 67(10):2186-2192. Doi: 10.1111/jgs.16077.
- Trilling JS. Selections from current literature assessment of older drivers. Family Practice 2001;18(3):339-42.
- Turk K, Dugan E. Research Brief: A Literature Review of Frontotemporal Dementia and Driving. Am J Alzheimers Dis Other Demen. 2014 Aug;29(5):404-8. doi: 10.1177/1533317513518656.
- Turk KM (2017), ‘Parkinson’s Disease and Lewy Body Dementia and Driving: A Review of the Literature’ Journal of Alzheimer’s Parkinsonism & Dementia. https://scientonline.org/open-access/parkinsons-disease-and-lewy-body-dementia-and-driving-a-review-of-the-literature.pdf
- Uc EY, Rizzo M. Driving and neurodegenerative diseases. Curr Neurol Neurosci Rep. 2008 Sep;8(5):377-83. doi: 10.1007/s11910-008-0059-1.
- Vair CL, Paul R. King, Julie Gass, April Eaker, Anna Kusche & Laura O. Wray (2018) Electronic Medical Record Documentation of Driving Safety for Veterans with Diagnosed Dementia, Clinical Gerontologist, 41:1, 66-76, DOI: 10.1080/07317115.2017.1312654
- Vanderbur, M., & Silverstein, M. M. (2006). Community Mobility and Dementia: A Review of the Literature. Washington, DC: Alzheimer’s Association Public Policy Division and the National Highway Traffic Safety Administration.
- Vardaki S, Yannis G, Papageorgiou SG (2014), ‘Assessing selected cognitive impairments using a driving simulator: a focused review’ Advances in Transportation Studies an international Journal Section B 34. https://www.nrso.ntua.gr/geyannis/wp-content/uploads/geyannis-pj99m.pdf
- Versijpt J, Tant M, Beyer I, Bier JC, Cras P, De Deyn PP, De Wit P, Deryck O, Hanseeuw B, Lambert M, Lemper JC, Mormont E, Petrovic M, Picard G, Salmon E, Segers K, Sieben A, Thiery E, Tournoy J, Vandewoude M, Ventura M, Verschraegen J, Engelborghs S, Goffin T, Deneyer M, Ivanoiu A. Alzheimer's disease and driving: review of the literature and consensus guideline from Belgian dementia experts and the Belgian road safety institute endorsed by the Belgian Medical Association. Acta Neurol Belg. 2017 Dec;117(4):811-819. doi: 10.1007/s13760-017-0840-5.
- Versijpt, J., Tant, M., Beyer, I. et al. Alzheimer’s disease and driving: review of the literature and consensus guideline from Belgian dementia experts and the Belgian road safety institute endorsed by the Belgian Medical Association. Acta Neurol Belg 117, 811–819 (2017). https://doi.org/10.1007/s13760-017-0840-5
- Vrkljan, B. H., McGrath, C. E., & Letts, L. J. (2011). Assessment tools for evaluating fitness to drive: A critical appraisal of evidence. Canadian Journal of Occupational Therapy, 78, 80-96. doi: 10.2182/cjot.2011.78.2.3

© 2022. Camilleri L & Whitehead D. Published online at http://www.aginganddisease.org/EN/10.14336/AD.2022.1126

SUPPLEMENTARY DATA

- Wagner JT, Müri RM, Nef T, Mosimann UP. Cognition and driving in older persons. Swiss Med Wkly. 2011 Jan 17;140:w13136. doi: 10.4414/smw.2011.13136. PMID: 21240690.
- Walsh L, Chacko E, Cheung G (2019), ‘The process of determining driving safety in people with dementia: A review of the literature and guidelines from 5 English speaking countries’ Australas Psychiatry; 27(5):480-485. doi: 10.1177/1039856219848828.
- Wang CC, Kosinski CJ, Schwartzberg JG et al. Physician’s Guide to Assessing and Counseling Older Drivers. Washington, DC: National Highway Traffic Safety Administration, 2003.
- Warlow C Driving safely with dementia Journal of Neurology, Neurosurgery & Psychiatry 2015;86:593-594. – TO BUY http://dx.doi.org/10.1136/jnnp-2015-310325
- Watanabe T, Konagaya Y, Yanagi T, Miyao M, Mukai M, Shibayama H. Study of daily driving characteristics of individuals with dementia using video-recording driving recorders. J Am Geriatr Soc. 2012 Jul;60(7):1381-3. doi: 10.1111/j.1532-5415.2012.04011.x.
- Wheatly CJ, Carr DB, Marottoli RA (2014), ‘Consensus Statements on Driving for Persons with Dementia’ Occupational Therapy In Health Care, 28:2, 132-139, DOI: 10.3109/07380577.2014.903583
- Wilson, S., & Pinner, G. (2013). Driving and dementia: A clinician’s guide. Advances in Psychiatric Medicine, 19, 89-96. doi: 10.1192/apt.bp.111.009555
- Withaar FK, Brouwer WH, van Zomeren AH. Fitness to drive in older drivers with cognitive impairment. J Int Neuropsychol Soc. 2000 May;6(4):480-90. doi: 10.1017/s1355617700644065.
- Wolfe PL, Katie A. Lehockey, Neuropsychological Assessment of Driving Capacity, Archives of Clinical Neuropsychology, Volume 31, Issue 6, September 2016, Pages 517–529, https://doi.org/10.1093/arclin/acw050
- Yale SH, Hansotia P, Knapp D, Ehrfurth J. Neurologic conditions: assessing medical fitness to drive. Clin Med Res. 2003 Jul;1(3):177-88. doi: 10.3121/cmr.1.3.177.
- Yamasaki T, Tobimatsu S (2018), ‘Driving Ability in Alzheimer Disease Spectrum: Neural Basis, Assessment, and Potential Use of Optic Flow Event-Related Potentials’ Front. Neurol. 9:750. doi: 10.3389/fneur.2018.00750
- You Joung K, Hoyoung A, Binna K, Young Shin P, Ki Woong K (2017), ‘An International Comparative Study on Driving Regulations on People with Dementia’ Journal of Alzheimer’s Disease; 56(3): 1007 – 1014. DOI: 10.3233/JAD-160762.
- Zuin, D.., Ortiz, H.., Boromei, D.. and Lopez, O.L.. (2002), Motor vehicle crashes and abnormal driving behaviours in patients with dementia in Mendoza, Argentina. European Journal of Neurology, 9: 29-34. https://doi.org/10.1046/j.1468-1331.2002.00296.x
- Holden A, Pusey H. The impact of driving cessation for people with dementia - An integrative review. Dementia (London). 2021 Apr;20(3):1105-1123. doi: 10.1177/1471301220919862.
- Lovas, Joel et al. ‘Assessment and Reporting of Driving Fitness in Patients with Dementia in Clinical Practice: Data from SveDem, the Swedish Dementia Registry’. 1 Jan. 2016 : 631 – 638.
- Ott BR and Daiello LA (2010), ‘How does dementia affect driving in older patients?’ AGING HEALTH; 1(6). DOI: https://doi.org/10.2217/ahe.09.83.
- Westphal, A. (2013). Cognitive Dementia and Memory Service Best Practice Guidelines: Service Guidelines for Victorian Cognitive Dementia and Memory Services.. State of Victoria, Department of Health.
- Kim YJ, An H, Kim B, Park YS, Kim KW (2017) An international comparative study on driving regulations on people with dementia. J Alzheimers Dis 56(3):1007–1014. doi:10.3233/ JAD-160762

4. Unpublished thesis, conference abstracts or book chapters (n= 49)

© 2022. Camilleri L & Whitehead D. Published online at http://www.aginganddisease.org/EN/10.14336/AD.2022.1126

SUPPLEMENTARY DATA

- Adler GL (2001), ‘Longitudinal Study of Driving Habits and Cessation in Older Persons with Dementia’ Accessed on https://www.proquest.com/openview/21b3c49405f18d62b519f9f20e475592/1?pq-origsite=gscholar&cbl=18750&diss=y
- Ali AA, Adler G and Rapaport M (2017), ‘Driving and Dementia—an Introduction, Educational Resources, and International Perspectives’ The American Journal of Geriatric Psychiatry; 25(3): 19-20. DOI:https://doi.org/10.1016/j.jagp.2017.01.045
- Allen, R. W., Park, G. D., Cook, M. L. and Fiorentino, D., 2007. A simulator for assessing older driver skills, 5th STISIM Drive User Group Meeting, Road Safety and Simulation RSS2007, Rome, Italy
- American Bar Association Commission on Law and Aging & American Psychological Association. (2008). Driving capacity. In Assessment of older adults with diminished capacity: A handbook for psychologists, (pp. 91–100). Washington, DC: Authors
- Andersen, G. J., 2011. Sensory and Perceptual Factors in the Design off Simulation Displays. Handbook of Driving Simulation for Engineering, Medicine and Psychology, CRC Press
- Anderson Sw, Rizzo M, Shi Q, Uc EY, Dawson JD (2005), ‘Cognitive Abilities Related to Driving Performance in a Simulator and Crashing on the Road’ Proceedings of the Third International Driving Symposium on Human Factors in Driver Assessment, Training and Vehicle Design, Rockport, Maine. Iowa City, IA: Public Policy Center, University of Iowa, 2005: 286-292. DOI: 10.17077/drivingassessment.1173.
- Austroads National Road Transport Commission (2012) Assessing Fitness to Drive for Commercial and Private Vehicles: Medical Standards for Licensing and Clinical Management Guidelines. NRTC, Sydney.
- Bakhtiari r, Michelle V. Tomczak, Stephen Langor, Joanna E.M. Scanlon, Aaron Granley, Anthony Singhal (2020), ‘Application of tablet-based cognitive tasks to predict unsafe drivers in older adults’ Transportation Research Interdisciplinary Perspectives; 4: 100105. https://doi.org/10.1016/j.trip.2020.100105.
- Ball, K.K. and Ackerman, M.L., 2011. The Older Driver (Training and Assessment: Knowledge, Skills, and Attitudes). Handbook of Driving Simulation for Engineering, Medicine and Psychology, CRC Press.
- Beratis IN, Koros C, Fragkiadaki S, Kontaxopoulou D, Pavlou D, Yannis G, Papageorgiou SG (https://www.nrso.ntua.gr/geyannis/wp-content/uploads/geyannis-cp186.pdf
- Beratis IN, Stanitsa E, Kontaxopoulou D, Fragkiadaki S, Pavlou D, Kontari P, Papageorgiou SG, Economou A, Yannis G, Papantoniou P ‘THE VALUE OF MINI MENTAL STATE EXAMINATION (MMSE) AND MONTREAL COGNITIVE ASSESSMENT (MoCA) IN THE PREDICTION OF FITNESS TO DRIVE IN PATIENTS WITH MILD COGNITIVE IMPAIRMENT (MCI) AND MILD ALZHEIMER’S DISEASE (AD)’ https://www.nrso.ntua.gr/geyannis/wp-content/uploads/geyannis-pc286.pdf
- Berndt A, May E, Clark M (2007), ‘Drivers with Dementia: Environment, Errors and Performance Outcomes’ In: Proceedings of the Fourth International Driving Symposium on Human Factors in Driver Assessment, Training and Vehicle Design, July 9-12, 2007, Stevenson, Washington. Iowa City, IA: Public Policy Center, University of Iowa, 2007: 401-407. https://doi.org/10.17077/drivingassessment.1268
- Byszewski A, Molnar F, Aminzadeh F. The impact of disclosure of unfitness to drive in persons with newly diagnosed dementia: patient and caregiver experiences Clin Gerontol 2009. In press.
- Caird, J. K. and Horrey, W. J., 2011. Twelve practical and useful questions about driving simulation. Handbook of Driving Simulation for Engineering, Medicine and Psychology, CRC Press.
- Canadian Medical Association. Determining Medical Fitness to Operate Motor Vehicles. CMA Driver’s Guide, 7th edition.
- Carmody J., Traynor V., Iverson D. & Andrew C. (2014) Dementia and Driving Decision Aid Booklet. Retrieved from dementia-driving@uow.edu.au on 24 November 2014.
- Carr D, Kennedy GJ, Rapaport M (2019), ‘DRIVING IN DEMENTIA: ADVANCES IN RESEARCH AND CLINICAL APPROACHES.: Session 414’ The American Journal of Geriatric Psychiatry; 27(3): S47-S48. https://doi.org/10.1016/j.jagp.2019.01.201
- Carr DB, Stowe JD, Morris JC. Driving in the elderly in health and disease. Handb Clin Neurol. 2019;167:563-573. doi: 10.1016/B978-0-12-804766-8.00031-5.
- Davis CE https://www.proquest.com/openview/9a6e6ef880f90dd607d910ae460f05bf/1?pq-origsite=gscholar&cbl=18750
- Carmody J, https://ro.uow.edu.au/cgi/viewcontent.cgi?referer=https://scholar.google.com/&httpsredir=1&article=5375&context=theses

© 2022. Camilleri L & Whitehead D. Published online at http://www.aginganddisease.org/EN/10.14336/AD.2022.1126

SUPPLEMENTARY DATA

- Dreher JW, "Development of a neurocognitive test battery to accurately predict driving ability in patients with Mild Cognitive Impairment and early Alzheimer's disease" (2013). Chancellor's Honors Program Projects. Accessed from https://trace.tennessee.edu/utk_chanhonoproj/1680
- Driving and dementia toolkit (for health professionals). 3rd ed. The Champlain Dementia Network, Regional Geriatric Program of Eastern Ontario; 2009. Available from: www.rgpeo.com/media/30695/dementiatoolkit.pdf.
- Edwards CJ, Creaser JI, Caird JK, Lamsdale AM, Chisholm SL. Older and younger driver performance at complex intersections: implications for using perception-response time and driving simulation. Proceedings of the Second International Driving Symposium on Human Factors in Driver Assessment, Training, and Vehicle Design, Park City, Utah. 2003:7-12.
- Etienne V, Marin-Lamellet C, Laurent B. Mental flexibility impairment in drivers with early Alzheimer's disease: A simulator-based study. IATSS Research. 2013; 37(1):16–20
- Hunter J, Brouwer W. Driver fitness, ability, skill and behavior. In: Hunter J, de Vries J, Brown Y, Hekstra A, et al., editors. Handbook of the disabled driver. Institute for Rehabilitation; Ljubljana, Republic of Slovenia:2009;16-20.
- Galski, T., & McDonald, M. A. (2009). Driving and the law. In Schultheis M. T., DeLuca J., & Chute D. (Ed.), Handbook for the assessment of driving capacity, (pp. 187–200). San Diego: Academic Press
- https://digital.library.adelaide.edu.au/dspace/bitstream/2440/22313/1/09phs6717.pdf
- Innes C, Jones R, Darlymple-Alford J, Severinsen J, Gray J (2009), 'Prediction of Driving Ability in People With Dementia- and Non- Dementia-Related Brain Disorders' Proceedings of the Fifth International Driving Symposium on Human Factors in Driver Assessment, Training and Vehicle Design; Big Sky, Montana. Iowa City, IA: Public Policy Center, University of Iowa, 2009: 342-348. DOI 10.17077/drivingassessment.1341
- Innes CR, Lee D, Chen C, Ponder-Sutton AM, Melzer TR, Jones RD. Do complex models increase prediction of complex behaviours? Predicting driving ability in people with brain disorders. Q J Exp Psychol (Hove). 2011 Sep;64(9):1714-25. doi: 10.1080/17470218.2011.555821.
- Innes CRH, D. Lee, C. Chen, A. M. Ponder-Sutton and R. D. Jones, "Different models for predicting driving performance in people with brain disorders," 2010 Annual International Conference of the IEEE Engineering in Medicine and Biology, 2010, pp. 5226-5229, doi: 10.1109/IEMBS.2010.5626280.
- Jang M, Son SJ, 'White matter hyperintensities as a new predictor of driving cessation in the elderly: a Clinical Research Center for Dementia of South Korea (CREDOS) Study' European neuropsychopharmacology. Conference: 29th european college of neuropsychopharmacology congress, ECNP 2016. Austria.
- Kawano N, Makino T, Suzuki Y, Umegaki H. Impact of driving cessation on daily transportation utility in elderly people with cognitive decline: a survey of patients in the memory clinic of an urban university hospital. Nihon Ronen Igakkai Zasshi 2009;46(5):420-7.
- Manore, M. and Papelis, Y., 2011. Roadway Visualization. Handbook of Driving Simulation for Engineering, Medicine and Psychology, CRC Press.
- Kirby J (2016), 'Examining the role of cognition in driving: Comparisons between driver groups and the development of the Maynooth On-Road Driving Assessment' Available from https://www.proquest.com/openview/a9941ddcc0f34fd71c5aae63263c6f4c/1?pq-origsite=gscholar&cbl=2026366&diss=y
- McGwin Jr, G., 2011. Independent Variables: The Role of Confounding and Effect Modification. Handbook of Driving Simulation for Engineering, Medicine and Psychology, CRC Press
- Papelis, Y., Ahmad, O. and Watson, G., 2003. Developing Scenarios to Determine Effects of Driver Performance: Techniques for Authoring and Lessons Learned, Conference paper, Document number N2003-010. Available from https://www.nadssc.uiowa.edu/publications.php?specificPub=N2003-010
- Piersma, D. (2018). Fitness to drive of older drivers with cognitive impairments. Rijksuniversiteit Groningen. Available from https://research.rug.nl/en/publications/fitness-to-drive-of-older-drivers-with-cognitive-impairments
- Robinson D, O'Neill D. Ethics of driving assessment in dementia care, competence and communication. In: Rai GS editor(s). Medical Ethics and the Elderly. Oxford: RadcliEe Publications, 2004:103-12.
- Sangeeta D https://www.proquest.com/openview/5aa9f56765a1e862eff33dff321a6542/1?pq-origsite=gscholar&cbl=18750&diss=y
- Silverstein N.M., Dickerson A.E., Schold Davis E. (2016) Community Mobility and Dementia: The Role for Health Care Professionals. In: Boltz M., Galvin J. (eds) Dementia Care. Springer, Cham. https://doi.org/10.1007/978-3-319-18377-0_9
- Silvertein NM https://deepblue.lib.umich.edu/bitstream/handle/2027.42/152940/alzjjalz200905475.pdf?sequence=1

© 2022. Camilleri L & Whitehead D. Published online at http://www.aginganddisease.org/EN/10.14336/AD.2022.1126

SUPPLEMENTARY DATA

- Singh, H., Barbour, B. M. and Cox, D. J., 2011. Driving Rehabilitation as Delivered by Driving Simulation. Handbook of Driving Simulation for Engineering, Medicine and Psychology, CRC Press
- Sirén, A., Heikkinen, S. and Hakamies-Blomqvist, L., 2001. Older female road users: A review, VTI Rapport 467A, Swedish National Road and Transport Research Institute, Linköping, Sweden,
- Sivak, Michael; Flannagan, George J; and Schoettle, Brandon. Driver Assessment and Training in the 1980s and 1990s: An Analysis of the Most Cited-Publications. In: Proceedings of the First International Driving Symposium on Human Factors in Driver Assessment, Training and Vehicle Design, 14-17 August 2001, Aspen, Colorado. Iowa City, IA: Public Policy Center, of Iowa, 2001: 21-25. https://doi.org/10.17077/ drivingassessment.1004
- Snellgrove SA, Cognitive screening for the safe driving competence of older people with mild cognitive impairment or early dementia. Google scholar. Available from https://www.infrastructure.gov.au/roads/safety/publications/2005/pdf/cog_screen_old.pdf
- Stamatelos P https://www.nrso.ntua.gr/geyannis/wp-content/uploads/geyannis-cp376.pdf
- Stern, R.A. & White, T. (2004). Neuropsychological Assessment Battery (NAB). Lutz, FL: Psychological Assessment Resources
- Uc, E. and Rizzo, M., 2011. Driving in Alzheimer's Disease, Parkinson's Disease, and Stroke. Handbook of Driving Simulation for Engineering, Medicine and Psychology, CRC Press.
- Leung, Judith, Psychological predictors of fitness to drive, Doctor of Psycology thesis, Faculty of Health and Behavioural Sciences, University of Wollongong, 2004. https://ro.uow.edu.au/theses/2135

5. <u>Diagnostic criteria or type of dementia not specified</u> {n= 35):

- Adler G, Rottunda S, Christensen K, Kuskowski M and Thuras P (2006), 'Driving SAFE: Development of a knowledge test for drivers with dementia' Sage Journals, 5(2): 213-222. https://doi.org/10.1177/1471301206062250
- Anstey K, J, Smith G, A: Associations of Biomarkers, Cognition and Self-Reports of Sensory Function with Self-Reported Driving Behaviour and Confidence. Gerontology 2003;49:196-202. doi: 10.1159/000069177
- Barco PP, Baum CM, Ott BR, Ice S, Johnson A, Wallendorf M, Carr DB (2015), 'Driving Errors in Persons with Dementia' J Am Geriatr Soc 63: 1373– 1380
- Barco PP, David B. Carr, Kathleen Rutkoski, Chengjie Xiong, Catherine M. Roe; Interrater Reliability of the Record of Driving Errors (RODE). Am J Occup Ther 2015;69(2):6902350020. https://doi.org/10.5014/ajot.2015.013128
- Berndt A., Clark M. and May E. (2008) 'Dementia severity and on-road assessment: Briefly revisited.' Australasian Journal on Ageing, 27: 157-160. https://doi.org/10.1111/j.1741-6612.2008.00300.x
- Bouman W (2010), 'A prospective study of cognitive tests to predict performance on a standardised road test in people with dementia' International journal of Geriatric psychiatry; 25: 489-496.
- Budd MA, Franks S and Hall JR (2003), 'Hooper Visual Organization Test (VOT) as a Predictor of Driving Status of Individuals with Dementia' Archives of Clinical Neuropsychology; 18(7):698-698.
- Bunt, R. and Lipski, P.S. (2003), Driving and dementia: a prospective audit of clients referred to an aged care assessment team. Australasian Journal on Ageing, 22: 215-217. https://doi.org/10.1111/j.1741-6612.2003.tb00502.x
- Carmody J, Potter J, Lewis K, Bhargava S, Trynor V, Iverson D (2014), 'Development and pilot testing of a decision aid for drivers with dementia' BMC Med Inform Decis Mak; 14(19). DOI: https://doi.org/10.1186/1472-6947-14-19
- Carr, D.B., Barco, P.P., Wallendorf, M.J., Snellgrove, C.A. and Ott, B.R. (2011), Predicting Road Test Performance in Drivers with Dementia. J Am Geriatr Soc, 59: 2112-2117. https://doi.org/10.1111/j.1532-5415.2011.03657.x
- Carr, D.B., Duchek, J. and Morris, J.C. (2000), Characteristics of Motor Vehicle Crashes of Drivers with Dementia of the Alzheimer Type. Journal of the American Geriatrics Society, 48: 18-22. https://doi.org/10.1111/j.1532-5415.2000.tb03023.x
- Crivelli L, Russo M, Bonetto M, Farez M, Sabe L, Allegri RF (2015), 'The association between neuropsychological functioning and driving performance in older people with mild dementia' Journal of the Neurological Sciences; 357:e215–e234. DOI:https://doi.org/10.1016/j.jns.2015.08.790.
- Eby DW, Nina M. Silverstein, Lisa J. Molnar, David LeBlanc, Geri Adler (2012), 'Driving behaviors in early stage dementia: A study using in-vehicle technology' Accident Analysis & Prevention; 49: 330-337. https://doi.org/10.1016/j.aap.2011.11.021.
- Eby, D.W. (2008), S3-05–03: Fitness to drive in early stage dementia: An instrumented vehicle study. Alzheimer's & Dementia, 4: T156-T156. https://doi.org/10.1016/j.jalz.2008.05.400

© 2022. Camilleri L & Whitehead D. Published online at http://www.aginganddisease.org/EN/10.14336/AD.2022.1126

SUPPLEMENTARY DATA

- Foley DJ, Masaki KH, Ross GW et al. Driving cessation on older men with incident dementia. J Am Geriatr Soc 2000;48:928–930.
- Falkmer T and Selander H. https://www.researchgate.net/profile/Torbjorn-Falkmer/publication/49363355_PREDICITVE_VALUE_OF_THE_NorSDSA_TO_ASSESS_DRIVING_IN_PERSONS_WITH_STROKE_OR_COGNITIVE_DEFICITSDEMENTIA/links/02e7e52096be1f0de2000000/PREDICITVE-VALUE-OF-THE-NorSDSA-TO-ASSESS-DRIVING-IN-PERSONS-WITH-STROKE-OR-COGNITIVE-DEFICITS-DEMENTIA.pdf
- Hunt LA, Brown AE, Gilman IP (2010), 'Drivers With Dementia and Outcomes of Becoming Lost While Driving.' Am J Occup Ther; 64(2):225–232. https://doi.org/10.5014/ajot.64.2.225
- Koppel S, Charlton J, Langford J, Vlahodimitrakou Z, Di Stefano M, Macdonald W, Mazer B, Gelinas I, Vrkljan B, & Marshall S (2013). The Relationship between Older Drivers' Performance on the Driving Observation Schedule (eDOS) and Cognitive Performance. Annals of advances in automotive medicine. Association for the Advancement of Automotive Medicine. Annual Scientific Conference, 57, 67–76.
- Lincoln LB, Taylor J, Radford KA (2012), 'Inter-rater reliability of the Nottingham Neurological Driving Assessment for people with dementia – a preliminary evaluation' Clin Rehabil.; 26(9):836-9. doi: 10.1177/0269215512442413.
- Lincoln NB, Taylor JL, Vella K, Bouman WP, Radford KA. A prospective study of cognitive tests to predict performance on a standardised road test in people with dementia. International Journal of Geriatric Psychiatry 2010;25(5):489-96.
- Lincoln, N.B., Radford, K.A., Lee, E. and Reay, A.C. (2006), The assessment of fitness to drive in people with dementia. Int. J. Geriat. Psychiatry, 21: 1044-1051. https://doi.org/10.1002/gps.1604
- Papandonatos GD, Ott BR, Davis JD, Barco PP, Carr DB. Clinical utility of the trail-making test as a predictor of driving performance in older adults. J Am Geriatr Soc 2015;63(11):2358-64.
- Ma'u E and Cheung G (2020), 'Ability of the Maze Navigation Test, Montreal Cognitive Assessment, and Trail Making Tests A & B to predict on-road driving performance in current drivers diagnosed with dementia' NZMA; 133(151513): 23-32.
- Ott BR, Davis JD, Papandonatos GD, Hewitt S, Festa EK, Heindel WC, Snellgrove CA, Carr DB (2013), 'Assessment of Driving-Related Skills Prediction of Unsafe Driving in Older Adults in the Office Setting' J Am Geriatr Soc 61:1164-1169. https://doi.org/10.1111/jgs.12306
- Pavlou D, Eleonora Papadimitriou, Constantinos Antoniou, Panagiotis Papantoniou, George Yannis, John Golias, Sokratis G. Papageorgiou (2017), 'Comparative assessment of the behaviour of drivers with Mild Cognitive Impairment or Alzheimer's disease in different road and traffic conditions' Transportation Research Part F: Traffic Psychology and Behaviour; 47: 122-131. https://doi.org/10.1016/j.trf.2017.04.019.
- Pavlou D, Ion Beratis, Eleonora Papadimitriou, Constantinos Antoniou, George Yannis, Sokratis Papageorgiou (2016), 'Which Are the Critical Measures to Assess the Driving Performance of Drivers with Brain Pathologies?' Transportation Research Procedia; 14: 4393-4402. https://doi.org/10.1016/j.trpro.2016.05.361.
- Piersma, D., Fuermaier, A.B.M., de Waard, D. et al. The MMSE should not be the sole indicator of fitness to drive in mild Alzheimer's dementia. Acta Neurol Belg 118, 637–642 (2018). https://doi.org/10.1007/s13760-018-1036-3
- Ranchet M, Tant M, Akinwuntan AE, Morgan JC, Devos H (2016), 'Fitness-to-drive Disagreements in Individuals With Dementia' Gerontologist; 57(5): 833–837. doi:10.1093/geront/gnw119
- Rottunda SJ, Lawler KL, Mc Carten R , The Driving Clinic: A Pilot Program for Veterans with Dementia. https://cdn.mdedge.com/files/s3fs-public/Document/September-2017/027040034.pdf
- Selander H, Kurt Johansson, Catarina Lundberg & Torbjörn Falkmer (2010) The Nordic Stroke Driver Screening Assessment as predictor for the outcome of an on-road test, Scandinavian Journal of Occupational Therapy, 17:1, 10-17, DOI: 10.3109/11038120802714898
- Stein AC, Dubinsky RM (2011), 'Driving simulator performance in patients with possible and probable Alzheimer's disease.' Ann Adv Automot Med.; 55:325-34
- Stout SH, Babulal GM, Ma C, Carr DB et.al. (2018), 'Driving cessation over a 24-year period: Dementia severity and cerebrospinal fluid biomarkers' Alzheimer's & Dementia; 14(5): 610-616. https://doi.org/10.1016/j.jalz.2017.11.011.
- Unsworth C and Chan SP (2016), 'Determining fitness to drive among drivers with Alzheimer's disease or cognitive decline' British Journal of Occupational Therapy; 79(2) 102–110
- Unsworth CA, Russell K, Lovell R, Woodward M, Browne M. Effect of Navigation Problems, Assessment Location, and a Practice Test on Driving Assessment Performance for People with Alzheimer's Disease. J Alzheimers Dis. 2019;67(3):1035-1043. doi: 10.3233/JAD-181069. PMID: 30776013.

© 2022. Camilleri L & Whitehead D. Published online at http://www.aginganddisease.org/EN/10.14336/AD.2022.1126

SUPPLEMENTARY DATA

- Vella K & Lincoln NB (2014) Comparison of assessments of fitness to drive for people with dementia, Neuropsychological Rehabilitation, 24:5, 770-783, DOI: 10.1080/09602011.2014.903197

6. Other neurological condition, unspecified or non-dementia related cognitive impairment included and dementia patients were not isolated (n= 211)

- Ackerman ML, PhD, Jerri D. Edwards, PhD, Lesley A. Ross, PhD, Karlene K. Ball, PhD, Melissa Lunsman, MS, Examination of Cognitive and Instrumental Functional Performance as Indicators for Driving Cessation Risk Across 3 Years, The Gerontologist, Volume 48, Issue 6, December 2008, Pages 802–810, https://doi.org/10.1093/geront/48.6.802
- Adrian J, Michèle Moessinger, André Charles, Virginie Postal (2019), 'Exploring the contribution of executive functions to on-road driving performance during aging: A latent variable analysis' Accident Analysis & Prevention; 127: 96-109. https://doi.org/10.1016/j.aap.2019.02.010.
- Adrian J, Virginie Postal, Michèle Moessinger, Nicole Rascle, André Charles (2011), 'Personality traits and executive functions related to on-road driving performance among older drivers' Accident Analysis & Prevention; 43(5): 1652-1659. https://doi.org/10.1016/j.aap.2011.03.023
- Akinwuntan AE, De Weerdt W, Feys H, Baten G, Arno P, Kiekens C. Reliability of a road test after stroke. Baten G, Arno P, Kiekens C. Arch Phys Med Rehabil 2003;84:1792-1796
- Akinwuntan AE, De Weerdt W, Feys H, Baten G, Arno P, Kiekens C. The validity of a road test after stroke. Arch Phys Med Rehabil 2005;86:421-426
- Akinwuntan AE, De Weerdt W, Feys H, et al. Effect of simulator training on driving after stroke: a randomized controlled trial. Neurology 2005;65:843-850
- Akinwuntan AE, Devos H, Feys H, et al. Confirmation of the accuracy of a short battery to predict fitness to drive of stroke survivors without severe deficits. J Rehabil Med 2007;39:698-702.
- Akinwuntan AE, Feys H, De Weerdt W, Baten G, Arno P, Kiekens C. Prediction of driving after stroke: a prospective study. Neurorehabil Neural Repair 2006;20:417-423.
- Akinwuntan, A. E., Wachtel, J., and Rosen, P. N., 2011. Driving simulation for evaluation and rehabilitation of driving after stroke, Journal of Stroke and Cerebrovascular Diseases, 21(6), p. 478-486.
- Alavi SS, Mohammadi MR, Souri H, Mohammadi Kalhori S, Jannatifard F, Sepahbodi G. Personality, Driving Behavior and Mental Disorders Factors as Predictors of Road Traffic Accidents Based on Logistic Regression. Iran J Med Sci. 2017;42(1):24-31.
- Al Banna, M., Redha, N. A., Abdulla, F., Nair, B., & Donnellan, C. (2016). Metacognitive function poststroke: A review of definition and assessment. Journal of Neurology, Neurosurgery, & Psychiatry, 87, 161–166.
- Alexandersen A, Knut Dalen & Kolbjørn Brønnick (2009) Prediction of driving ability after inconclusive neuropsychological investigation, Brain Injury, 23:4, 313-321, DOI: 10.1080/02699050902788428
- Alonso AC, Mark D. Peterson, Alexandre L. Busse, Wilson Jacob-Filho, Mauricio T.A. Borges, Marcos M. Serra, Natalia M.S. Luna, Paulo H. Marchetti, Júlia M.D.A. Greve (2016), 'Muscle strength, postural balance, and cognition are associated with braking time during driving in older adults' Experimental Gerontology; 85: 13-17. https://doi.org/10.1016/j.exger.2016.09.006
- Anderson SW, Nazan Aksan, Jeffrey D. Dawson, Ergun Y. Uc, Amy M. Johnson & Matthew Rizzo (2012) Neuropsychological assessment of driving safety risk in older adults with and without neurologic disease, Journal of Clinical and Experimental Neuropsychology, 34:9, 895-905, DOI: 10.1080/13803395.2011.630654
- Andrews E.C. and Westerman S.J., 2012. Age differences in simulated driving performance: Compensatory processes, Accident Analysis & Prevention, 45, p. 660-668
- Anstey KJ, Mark S. Horswill, Joanne M. Wood, Christopher Hatherly (2012), 'The role of cognitive and visual abilities as predictors in the Multifactorial Model of Driving Safety' Accident Analysis & Prevention; 45: 766-774. https://doi.org/10.1016/j.aap.2011.10.006
- Anstey KJ, Wood J, Caldwell H, Kerr G, Lord SR. Comparison of self-reported crashes, state crash records and an on-road driving assessment in a population-based sample of drivers aged 69-95 years. Tra!ic Injury Prevention 2009;10(1):84-90
- Arno, P. and Boets, S., 2003, Aged people Integration, mobility, safety and quality of Life Enhancement through driving (AGILE) Project, Deliverable 5.2, Elderly driver's integrated assessment methodology. http://www.agile.iao.fraunhofer.de/downloads/agile_d5_2.pdf
- Asimakopulos, J., Boychuck, Z., Sondergaard, D., Poulin, V., Ménard, I. and Korner-Bitensky, N. (2012), Assessing executive function in relation to fitness to drive: A review of tools and their ability to predict safe driving. Australian

Occupational Therapy Journal, 59: 402-427. https://onlinelibrary.wiley.com/doi/epdf/10.1111/j.1440-1630.2011.00963.x
- Babulal GM, Stout SH, Benzinger TLS, Ott BR, Carr DB, Webb M, Traub CM, Addison A, Morris JC, Warren DK, Roe CM. A Naturalistic Study of Driving Behavior in Older Adults and Preclinical Alzheimer Disease: A Pilot Study. J Appl Gerontol. 2019 Feb;38(2):277-289. doi: 10.1177/0733464817690679
- Babulal GM. et al. 'Neuropsychiatric Symptoms and Alzheimer's Disease Biomarkers Predict Driving Decline: Brief Report'. 1 Jan. 2017 : 675 – 680-
- Baldock MRJ, Mathias JL, McLean AJ, Berndt A. Self regulation of driving and its relationship to driving ability among older adults. Accid Anal Prev 2006;38:1038 –1045
- Ball KK, Roenker DL, Wadley VG, Edwards JD, Roth DL, McGwin G, Jr, et al. Can high-risk older drivers be identified through performance-based measures in a Department of Motor Vehicles setting?. Journal of the American Geriatrics Society 2006;54(1):77-84
- Bellagamba D, Vionette L, Margot-Cattin I, Vaucher P (2020), 'Standardized on-road tests assessing fitness-to-drive in people with cognitive impairments: A systematic review' PLoS ONE 15(5): e0233125. https://doi.org/10.1371/journal.pone.0233125 - people other than dementia
- Benedetto, A., 2008. Older drivers and safety: a roadway study using driving simulator, Advances in Transportation Studies, XVI(A), pp.29-42
- Berndt AH, May E and Darzins P (2015), 'On-road driving assessment and route design for drivers with dementia' British journal of occupational therapy; 78(2): 121-130. DOI: https://doi.org/10.1177/0308022614562397
- Bieliauskas LA (2005) Neuropsychological assessment of geriatric driving competence, Brain Injury, 19:3, 221-226, DOI: 10.1080/02699050400017213
- Biernacki MP and Lewkowicz R (2020), 'Evidence for the role of personality in the cognitive performance of older male drivers' Transportation Research Part F: Traffic Psychology and Behaviour; 69: 385-400. https://doi.org/10.1016/j.trf.2020.02.005
- Blane A, Hoe Lee, Torbjörn Falkmer, Tania Dukic Willstrand (2018), 'Cognitive ability as a predictor of task demand and self-rated driving performance in post-stroke drivers – Implications for self-regulation' Journal of Transport & Health; 9: 169-179. https://doi.org/10.1016/j.jth.2018.01.013.
- Bliokas VV, Joanne E. Taylor, Judith Leung & Frank P. Deane (2011) Neuropsychological assessment of fitness to drive following acquired cognitive impairment, Brain Injury, 25:5, 471-487, DOI: 10.3109/02699052.2011.559609 -
- Brady, B., Eramudugolla, R., Wood, J. M., & Anstey, K. J. (2021). Association between decision-making under risk conditions and on-road driving safety among older drivers. Neuropsychology. Advance online publication. https://doi.org/10.1037/neu0000754
- Cantin, V., Lavallière, M., Simoneau, M. and Teasdale, N., 2009. Mental workload when driving in a simulator: Effects of age and driving complexity, Accident Analysis & Prevention, 41(4), p. 763-771 – non dementia patients
- Choi M, Lohman MC, Mezuk B (2014) Trajectories of cognitive decline by driving mobility: evidence from the Health and Retirement Study. Int J Geriatr Psychiatry 29(5):447–453. doi:10.1002/gps.4024
- Chua, M., McCluskey, A. and Smead, J.M. (2012), Retrospective analysis of factors that affect driving assessment outcomes after stroke. Aust Occup Ther J, 59: 121-130. https://doi.org/10.1111/j.1440-1630.2012.01005.x
- Cizman Staba U, Tara Klun, Kristina Stojmenova, Grega Jakus & Jaka Sodnik (2020) Consistency of neuropsychological and driving simulator assessment after neurological impairment, Applied Neuropsychology: Adult, DOI: 10.1080/23279095.2020.1815747
- Crizzle AM, Sherrilene Classen, Michel Bédard, Desiree Lanford, Sandra Winter (2012), 'MMSE as a predictor of on-road driving performance in community dwelling older drivers' Accident Analysis & Prevention; 49: 287-292. https://doi.org/10.1016/j.aap.2012.02.003
- Crotty M, George S (2009), 'Retraining Visual Processing Skills To Improve Driving Ability After Stroke' Archives of Physical Medicine and Rehabilitation; 90(12): 2096-2102. https://doi.org/10.1016/j.apmr.2009.08.143.
- Cyr, A. A., Stinchcombe, A., Gagnon, S., Marshall, S., Hing, M. M. S., & Finestone, H. (2009). Driving difficulties of brain-injured drivers in reaction to high-crash-risk simulated road events: a question of impaired divided attention? Journal of Clinical and Experimental Neuropsychology, 31, 472–482.
- Daigneault G, Joly P, Frigon J-Y. Previous convictions or accidents and the risk of subsequent accidents of older drivers. Accid Anal Prev 2002;34:257–261.
- Danciu B, Popa C, Ioan Micle M, Preda G (2012), 'Psychological risk factors for road safety' Procedia - Social and Behavioral Sciences; 33: 363-367. https://doi.org/10.1016/j.sbspro.2012.01.144
- De Raedt R, Ponjaert-Kristoffersen I. Short cognitive/neuropsychological test battery for first-tier fitness-to-drive assessment of older adults. Clin Neuropsychol 2001;15: 329 –336

SUPPLEMENTARY DATA

- De Raedt R, Ponjaert-Kristoffersen I. The relationship between cognitive/neuropsychological factors and car driving performance in older adults. J Am Geriatr Soc. 2000 Dec;48(12):1664-8. doi: 10.1111/j.1532-5415.2000.tb03880.x.
- De Raedt, R. and Ponjaert-Kristoffersen, I., 2000. Can Strategic and tactical compensation reduce crash risk in older drivers? Age & Ageing, 29, pp. 517-521
- Depestele S, Veerle Ross, Stefanie Verstraelen, Kris Brijs, Tom Brijs, Kim van Dun, Raf Meesen (2020), 'The impact of cognitive functioning on driving performance of older persons in comparison to younger age groups: A systematic review' Transportation Research Part F: Traffic Psychology and Behaviour; 73: 433-452. https://doi.org/10.1016/j.trf.2020.07.009.
- Devlin A, Jane McGillivray (2016), 'Self-regulatory driving behaviours amongst older drivers according to cognitive status' Transportation Research Part F: Traffic Psychology and Behaviour; 39: 1-9. https://doi.org/10.1016/j.trf.2016.02.001.
- Devlin, A. and McGillivray, J.A. (2014), Self-regulation of older drivers. Australasian Journal on Ageing, 33: 74-80. https://doi.org/10.1111/ajag.12061
- Devlin, A., McGillivray, J., Charlton, J., Lowndes, G. and Etienne, V., 2012. Investigating driving behaviour of older drivers with mild cognitive impairment using a portable driving simulator, Accident Analysis & Prevention
- Devos H, Akinwuntan AE, Nieuwboer A, Truijen S, Tant M, De Weerdt W(2011), 'Screening for fitness to drive after stroke: A systematic review and meta-analysis' Neurology Feb 2011, 76 (8) 747-756; DOI: 10.1212/WNL.0b013e31820d6300
- Dickerson A, Reistetter T and Trujillo L (2009), Using an IADL Assessment to Identify Older Adults Who Need a Behind-the-Wheel Driving Evaluation' Journal of applied Gerontology; 29(4): 494-506. https://doi.org/10.1177/0733464809340153
- Dickerson AE, Danielle Brown Meuel, Cyrus David Ridenour, Kristen Cooper; Assessment Tools Predicting Fitness to Drive in Older Adults: A Systematic Review. Am J Occup Ther 2014;68(6):670–680. https://doi.org/10.5014/ajot.2014.011833 -
- Dijksterhuis, C., Brookhuis, K. A. and De Waard, D., 2011. Effects of steering demand on lane keeping behaviour, self-reports, and physiology. A simulator study, Accident Analysis & Prevention, 43(3), p. 1074-1081.
- Di Stefano, M., Stefano, M. D., & Macdonald, W. (2012). Design of occupational therapy on-road test routes and related validity issues. Australian Occupational Therapy Journal, 59(1), 37–46. https://doi.org/10.1111/J.1440-1630.2011.00990.X
- Di Stefano, M., Stefano, M. D., & Macdonald, W. (2003). Assessment of older drivers: Relationships among on-road errors, medical conditions and test outcome. Journal of Safety Research, 34(4), 415–429. https://doi.org/10.1016/J.JSR.2003.09.010
- Dobbs AR (2013), 'Accuracy of the DriveABLE cognitive assessment to determine cognitive fitness to drive' Canadian Family Physician Mar, 59 (3) e156-e161
- Dobbs BM, Schopflocher D. The Introduction of a New Screening Tool for the Identification of Cognitively Impaired Medically At-Risk Drivers: The SIMARD A Modification of the DemTect. J Prim Care Community Health. 2010 Jul 1;1(2):119-27. doi: 10.1177/2150131910369156.
- Dobbs BM, Simran S. Shergill, How effective is the Trail Making Test (Parts A and B) in identifying cognitively impaired drivers?, Age and Ageing, Volume 42, Issue 5, September 2013, Pages 577–581, https://doi.org/10.1093/ageing/aft073
- Dobbs BM, Wodzin E, Vegega M (2005), 'Medical conditions and driving : a review of the literature (1960-2000)' Repository and Open Science Access Portal [online]. Available from https://rosap.ntl.bts.gov/view/dot/1902. Accessed on 2nd August 2021.
- Duncanson H, Ann M. Hollis, Margaret G. O'Connor (2018), 'Errors versus speed on the trail making test: Relevance to driving performance' Accident Analysis & Prevention; 113: 125-130. https://doi.org/10.1016/j.aap.2018.01.004.
- Eby, D. W., Molnar, L. J., Shope J. T., & Dellinger, A. M. (2007). Development and pilot testing of an assessment battery for older drivers. Journal of Safety Research, 38(5),535-543. https://doi.org/10.1016/j.jsr.2007.07.004.
- Elkin-Frankston S, Brian K. Lebowitz, Lissa R. Kapust, Ann M. Hollis, Margaret G. O'Connor, The use of the Color Trails Test in the assessment of driver competence: Preliminary report of a culture-fair instrument, Archives of Clinical Neuropsychology, Volume 22, Issue 5, June 2007, Pages 631–635, https://doi.org/10.1016/j.acn.2007.04.004
- Ellison J (2012), "Introduction: dementia, delirium, depression, drugs, and driving." Psychiatric Times; 29(8): 34. Available from link.gale.com/apps/doc/A301776603/AONE?u=anon~d9649bf9&sid=googleScholar&xid=65ecc6a0. Accessed 4 Aug. 2021.

© 2022. Camilleri L & Whitehead D. Published online at http://www.aginganddisease.org/EN/10.14336/AD.2022.1126

SUPPLEMENTARY DATA

- Espie, S., Gauriat, P. and Duraz, M., 2005. Driving Simulators validation. Driver Simulation Conference, North America, Orlando, FL
- Esser P, Dent S, Jones C, et al Utility of the MOCA as a cognitive predictor for fitness to drive' Journal of Neurology, Neurosurgery & Psychiatry 2016;87:567-568.
- Falkenstein M, Karthaus M, Brüne-Cohrs U. Age-Related Diseases and Driving Safety. Geriatrics (Basel). 2020 Oct 19;5(4):80. doi: 10.3390/geriatrics5040080.
- Fausto BA, McIntosh B, Bonner C, McBride AM (2016), 'Neuropsychological Tests and Measurement of Comorbidity in Predicting Driving Competence among Memory Clinic Patients' Psychology Research, October 2016, Vol. 6, No. 10, 559-566. doi:10.17265/2159-5542/2016.10.001
- Ferreira IS, Mário R. Simões, João Marôco (2012), 'The Addenbrooke's Cognitive Examination Revised as a potential screening test for elderly drivers' Accident Analysis & Prevention; 49: 278-286. https://doi.org/10.1016/j.aap.2012.03.036. –
- Ferreira IS, Simões MR, Marôco J (2013), 'Cognitive and psychomotor tests as predictors of on-road driving ability in older primary care patients' Transportation Research Part F: Traffic Psychology and Behaviour; 21: 146-158. https://doi.org/10.1016/j.trf.2013.09.007.-
- Fields, S.M. and Unsworth, C.A. (2017), Revision of the Competency Standards for Occupational Therapy Driver Assessors: An overview of the evidence for the inclusion of cognitive and perceptual assessments within fitness-to-drive evaluations. Aust Occup Ther J, 64: 328-339. https://doi.org/10.1111/1440-1630.12379 - https://onlinelibrary.wiley.com/doi/epdf/10.1111/1440-1630.12379 -
- Freund B, Szinovacz M (2002), 'Effects of Cognition on Driving Involvement Among the Oldest Old: Variations by Gender and Alternative Transportation Opportunities' The Gerontologist; 42(5): 621–633, https://doi.org/10.1093/geront/42.5.621
- Freund, B., Gravenstein, S., Ferris, R. et al. Drawing clocks and driving cars. J GEN INTERN MED 20, 240–244 (2005). https://doi.org/10.1111/j.1525-1497.2005.40069.x
- Gardezi F, Wilson KG, Man-Son-Hing M et al. Qualitative research on older drivers. Clin Gerontol 2006;30:5–22.4
- George S, Clark M, Crotty M. Development of the Adelaide driving self-efficacy scale. Clin Rehabil. 2007 Jan;21(1):56-61. doi: 10.1177/0269215506071284.
- George S, Maria Crotty; Establishing Criterion Validity of the Useful Field of View Assessment and Stroke Drivers' Screening Assessment: Comparison to the Result of On-Road Assessment. Am J Occup Ther 2010;64(1):114–122. https://doi.org/10.5014/ajot.64.1.114
- Gibbons C, Nathan Smith, Randy Middleton, John Clack, Bruce Weaver, Sacha Dubois, Michel Bédard; Using Serial Trichotomization With Common Cognitive Tests to Screen for Fitness to Drive. Am J Occup Ther 2017;71(2):7102260010. https://doi.org/10.5014/ajot.2017.019695
- Giuliano G, Hu H, Lee K. Travel Patterns in the Elderly: the Role of Land Usage. Los Angeles, CA: METRANS Transportation Center, 2003. [METRANS project 00-8]
- Greene WR, Randi Smith (2019), 'Driving in the Geriatric Population' Clinics in Geriatric Medicine; 35(1): 127-131. https://doi.org/10.1016/j.cger.2018.08.011.
- Hajek A, Christian Brettschneider, Marion Eisele, Hendrik van den Bussche, Birgitt Wiese, Silke Mamone, Siegfried Weyerer, Jochen Werle, Verena Leve, Michael Pentzek, Susanne Röhr, Janine Stein, Horst Bickel, Edelgard Mösch, Kathrin Heser, Michael Wagner, Martin Scherer, Wolfgang Maier, Steffi G. Riedel-Heller, Hans-Helmut König (2019), 'Prevalence and determinants of driving habits in the oldest old: Results of the multicenter prospective AgeCoDe-AgeQualiDe study' Archives of Gerontology and Geriatrics; 82: 245-250. https://doi.org/10.1016/j.archger.2019.03.006.
- Hakamies-Blomqvist L, Wiklund M, Henriksson P. Predicting older drivers' involvement: FSmeed's law revisited. Accid Anal Prev 2005;37:675–680.52
- Hakamies-Blomqvist, L. and Peters, B., 2000. Recent European research on older drivers, Accident Analysis & Prevention, 32, p. 601-607
- Hakamies-Blomqvist, L., 2004. Safety of Older Persons in Traffic. Proc., Transportation in an Ageing Society. A Decade of Experience, Transportation Research Board, Washington D.C., 22-35.
- Hargrave DD, Jason M. Nupp & Rey J. Erickson (2012) Two brief measures of executive function in the prediction of driving ability after acquired brain injury, Neuropsychological Rehabilitation, 22:4, 489-500, DOI: 10.1080/09602011.2012.662333
- Harris, M. (2000). Psychiatric conditions with relevance to fitness to drive. Advances in Psychiatric Treatment, 6(4), 261-269. doi:10.1192/apt.6.4.261

© 2022. Camilleri L & Whitehead D. Published online at http://www.aginganddisease.org/EN/10.14336/AD.2022.1126

SUPPLEMENTARY DATA

- Hartman-Maeir A, Asnat Bar-Haim Erez, Navah Ratzon, Tatiana Mattatia & Penina Weiss (2008) The validity of the Color Trail Test in the pre-driver assessment of individuals with acquired brain injury, Brain Injury, 22:13-14, 994-998, DOI: 10.1080/02699050802491305
- Hemmy, Laura, Susan Rottunda, and Geri Adler. 2016. "The Older Driver with Cognitive Impairment: Perceptions of Driving Ability and Results of a Behind the Wheel Test" Geriatrics 1, no. 1: 6. https://doi.org/10.3390/geriatrics1010006
- Hines, A. and Bundy, A.C. (2014), Predicting driving ability using DriveSafe and DriveAware in people with cognitive impairments: A replication study. Aust Occup Ther J, 61: 224-229. https://doi.org/10.1111/1440-1630.12112
- Hiraoka T et.al. (2015), ‘Investigation into the safety of driving by individuals with higher brain dysfunction’ Kawasaki Medical Journal 41（2） : 71－81, 2015 doi : 10.11482/KMJ-E41（2）7
- Hird MA, Vetivelu A, Saposnik G, Schweizer TA (2014), ‘Cognitive, On-road, and Simulator-based Driving Assessment after Stroke’ Journal of Stroke and Cerebrovascular Diseases; 23(10): 2654-2670. https://doi.org/10.1016/j.jstrokecerebrovasdis.2014.06.010
- Hogan D, Bedard M. Review of the introduction of a new screening tool for the identification of cognitively impaired medically at-risk drivers. Can Geriatr J 2011;14(2):51–4; http://cgjonline.ca/index.php/cgj/article/view/12/31
- Hogan, D. B., & Bédard, M. (2011). Papers that might change your practice: review of the introduction of a new screening tool for the identification of cognitively impaired medically at-risk drivers. Canadian geriatrics journal : CGJ, 14(2), 51–54. https://doi.org/10.5770/cgj.v14i2.12
- Hoggarth PA, Carrie R.H. Innes, John C. Dalrymple-Alford, Richard D. Jones (2015), ‘Prediction of driving ability: Are we building valid models?’ Accident Analysis & Prevention; 77: 29-34. https://doi.org/10.1016/j.aap.2015.01.013.
- Hoggarth PA, https://ir.canterbury.ac.nz/handle/10092/5384-
- Hoggarth PA, Innes CRH, Dalrymple-Alford JC, Jones RD (2013), ‘Predicting On-Road Assessment Pass and Fail Outcomes in Older Drivers with Cognitive Impairment Using a Battery of Computerized Sensory-Motor and Cognitive Tests’ J Am Geriatr Soc 61: 2192– 2198. https://doi.org/10.1111/jgs.12540
- Hoggharth P, Innes C https://ir.uiowa.edu/drivingassessment/2011/papers/50/
- Hollis AM, Duncanson H, Kapust LA, Xi Ma PM, O’Conner MG (2015), Validity of the Mini-Mental State Examination and the Montreal Cognitive Assessment in the Prediction of Driving Test Outcome’ J Am Geriatr Soc 63: 988– 992, 2015. https://doi.org/10.1111/jgs.13384
- HORIKAWA E, Ryo MORIZONO, Akemi KOGA, Jun HORIE (2009), ‘ELDERLY DRIVING BEHAVIOR AND COGNITIVE FUNCTIONS: Analysis of License Renewal Course Data’ IATSS Research; 3(1): 18-26. https://doi.org/10.1016/S0386-1112(14)60233-8.
- https://esmed.org/MRA/mra/article/view/1035 –
- Ida Sletmo Torgerstuen and Mathilde Suhr Hemminghyth ‘Driving safety after brain injury: Relationships between cognitive and executive functions, driving behaviour, and accident involvement’ https://www.duo.uio.no/bitstream/handle/10852/59716/Driving-safety-after-brain-injury.pdf?sequence=1&isAllowed=y
- Innes CRH, Richard D. Jones, John C. Dalrymple-Alford, Sarah Hayes, Sue Hollobon, Julie Severinsen, Gwyneth Smith, Angela Nicholls, Tim J. Anderson (2007), ‘Sensory-motor and cognitive tests predict driving ability of persons with brain disorders’ Journal of the Neurological Sciences; 260(1-2_: 188-198. https://doi.org/10.1016/j.jns.2007.04.052.
- Jones Ross RW, Scialfa CT, Cordazzo STD (2015), ‘Predicting On-Road Driving Performance and Safety in Cognitively Impaired Older Adults’ J Am Geriatr Soc 63: 2365– 2369. https://doi.org/10.1111/jgs.13712
- Joseph PG, O'Donnell MJ, Teo KK, Gao P, Anderson C, Probstfield JL, Bosch J, Khatib R, Yusuf S. The mini-mental state examination, clinical factors, and motor vehicle crash risk. J Am Geriatr Soc. 2014 Aug;62(8):1419-26. doi: 10.1111/jgs.12936
- Justiss MD (2005), ‘Development of a behind-the-wheel driving performance assessment for older adults’ https://www.proquest.com/openview/55d591076b5ce53ed30f1cd9b8bafc4a/1?pq-origsite=gscholar&cbl=18750&diss=y – not exclusive to dementia patients
- Kandasamy D, Kayla Williamson, David B. Carr, Diana Abbott, Marian E. Betz (2019), ‘The utility of the Montreal Cognitive Assessment in predicting need for fitness to drive evaluations in older adults’ Journal of Transport & Health; 13: 19-25. https://doi.org/10.1016/j.jth.2019.03.005-
- Katsouri I, Athanasiadis L, Bekiaris E, Tsolaki M. Differences between professional and non-professional drivers with cognitive disorders. Hell J Nucl Med. 2019 Jan-Apr;22 Suppl:17-31. PMID: 30877720.

SUPPLEMENTARY DATA

- Kay L., Bundy A. & Clemson L. (2009a) Awareness of driving ability in senior drivers with neurological conditions. American Journal of Occupational Therapy 63(2), 146–150.
- Kay LG, Anita C. Bundy, Lindy M. Clemson (2009), 'Predicting Fitness to Drive in People With Cognitive Impairments by Using DriveSafe and DriveAware' Archives of Physical Medicine and Rehabilitation; 90(9): 1514-1522. https://doi.org/10.1016/j.apmr.2009.03.011
- Kay LG, Bundy AC, Clemson LM https://conference2006.acspri.org.au/proceedings/streams/Paper%2009%20ACSPRI%202006.pdf
- Kay, L., Bundy, A., & Clemson, L. (2009). Validity, reliability and predictive accuracy of the driving awareness questionnaire. Disability and Rehabilitation, 31, 1074–1082.
- Keall MD, Frith WJ. Association between older driver characteristics, on-road driving test performance, and crash liability. Traffic Inj Prev 2004;5:112–116
- Khan, R., Khan, M.T. & Alam, B. The use of neuropsychological tests to study the effects of aging on driving performance in the UK. Eur. Transp. Res. Rev. 10, 15 (2018). https://doi.org/10.1007/s12544-018-0287-7
- Kilik L, Fogarty JN, Hopkins RW. Medical Driving Assessment Outcomes in Seniors Using The Kscar+Drive: An In-Office Screening Tool to Assist Clinicians in Determining Driving Safety and Who to Refer for Medical Driving Assessments. J Parkinsons Dis Alzheimer Dis. 2018;5(2): 5.
- Klavora P, Ronald J. Heslegrave, Margaret Young (2000), 'Driving skills in elderly persons with stroke: Comparison of two new assessment options' Archives of Physical Medicine and Rehabilitation; 81(6): 701-705. https://doi.org/10.1016/S0003-9993(00)90096-0.
- Knoblauch, R., Nitzburg, M. and Seifert, R., 1997. An Investigation of Older Driver Freeway Needs and Capabilities. Federal Highway Administration, McLean VA. Advances in Transportation Studies an international Journal Section B 34 (2014) - 128
- Korner-Bitensky N, Sofer S. The DriveABLE Competence Screen as a predictor of on-road driving in a clinical sample. Aust Occup Ther J. 2009;56(3):200–205.
- Kowalski K, Jeznach A, Tuokko HA. Stages of driving behavior change within the Transtheoretical Model (TM). J Safety Res. 2014 Sep;50:17-25. doi: 10.1016/j.jsr.2014.01.002.
- Kwak, H. S.; Kim, J. Y.; Jung, B. K. (2015), 'An Experimental Study for Testing Two Different Input Methods, Mouse and Touch Screen, to Explore Impact on Reliability and Validity of Driving Assessment' Advanced Science Letters; 21(3): 447-450. DOI: https://doi.org/10.1166/asl.2015.5807
- Kwok, J. C. W., Gélina, I., & Benoit, D., & Chilingaryan, G. (2015). Predictive validity of the Montreal Cognitive Assessment (MoCA) as a screening tool for on-road driving performance. British Journal of Occupational Therapy, 78(2), 100-108. https://doi.org/10.1177%2F0308022614562399.
- Larrson H and Falkmar T https://ir.uiowa.edu/cgi/viewcontent.cgi?article=1258&context=drivingassessment
- Ledger S, Joanne M. Bennett, Eugene Chekaluk, Jennifer Batchelor (2019), 'Cognitive function and driving: Important for young and old alike' Transportation Research Part F: Traffic Psychology and Behaviour; 60: 262-273. https://doi.org/10.1016/j.trf.2018.10.024
- Lees MN, Cosman JD, Lee JD, Fricke N, Rizzo M. Translating cognitive neuroscience to the driver's operational environment: a neuroergonomic approach. Am J Psychol. 2010;123(4):391-411. doi:10.5406/amerjpsyc.123.4.0391
- Lee HC, Andy H. Lee; Identifying Older Drivers at Risk of Traffic Violations by Using a Driving Simulator: A 3-Year Longitudinal Study. Am J Occup Ther 2005;59(1):97–100. https://doi.org/10.5014/ajot.59.1.97
- Lee, H. C., Cameron, D., & Lee, A. H. (2003). Assessing the driving performance of older adult drivers: on-road versus simulated driving. Accident Analysis and Prevention, 35, 797 – 803
- Lengenfelder, J., Schultheis, M. T., Al Shihabi, T., Mourant, R., & DeLuca, J. (2002). Divided attention and driving: A pilot study using virtual reality technology. Journal of Head Trauma Rehabilitation, 17, 26–37.
- León-Domínguez U, Ignacio Solís-Marcos, Cristina Alejandra López-Delgado, Juan Manuel Barroso y Martín, José León-Carrión (2020), 'A Frontal Neuropsychological Profile in Fitness to Drive' Accident Analysis & Prevention; 142: 105807. https://doi.org/10.1016/j.aap.2020.105807
- Leproust S, Lagarde E, Salmi LR. Risks and advantages of detecting individuals unfit to drive: a Markov decision analysis. Journal of General Internal Medicine 2008;23(11):1796-803.
- Lesikar SE, Gallo JJ, Rebok GW, Keyl PM. Prospective study of brief neuropsychological measures to assess crash risk in older primary care patients. JABFP 2002;15:11–19.
- Leung J, Deane FP, Taylor JE, Bliokas VV. Anxiety in driving assessment of individuals with cognitive impairment. Disabil Rehabil. 2009;31(20):1700-8. doi: 10.1080/09638280902738581.
- Li G, Eby DW, Santos R, et al. Longitudinal Research on Aging Drivers (LongROAD): study design and methods. Inj Epidemiol. 2017;4(1):22. doi:10.1186/s40621-017-0121-z

SUPPLEMENTARY DATA

- Liebherr M, Stephanie Antons, Stephan Schweig, Niko Maas, Dieter Schramm, Matthias Brand (2019), 'Driving performance and specific attentional domains' Transportation Research Interdisciplinary Perspectives; 3:100077. https://doi.org/10.1016/j.trip.2019.100077
- Lindstrom-Forneri W, Tuokko HA, Douglas Garrett BA & Frank Molnar MD (2010) Driving as an Everyday Competence: A Model of Driving Competence and Behavior, Clinical Gerontologist, 33:4, 283-297, DOI: 10.1080/07317115.2010.502106.
- MacGregor JM, Freeman DH, Zhang D. A traffic sign recognition test can discriminate between older drivers who have and have not had a motor vehicle crash. J AmGeriatr Soc 2001;49:466 – 469.
- MacLeod KE, William A. Satariano, David R. Ragland (2014), 'The impact of health problems on driving status among older adults' Journal of Transport & Health; 1(2): 86-94. https://doi.org/10.1016/j.jth.2014.03.001
- Mallon K, Joanne M. Wood; Occupational Therapy Assessment of Open-Road Driving Performance: Validity of Directed and Self-Directed Navigational Instructional Components. Am J Occup Ther 2004;58(3):279–286. https://doi.org/10.5014/ajot.58.3.279
- Man-Son-Hing M, Marshall SC, Molnar FJ, Wilson KG, Crowder C, Chambers LW. A Canadian research strategy for older drivers: the CanDRIVE program. Geriatr Today. J Can Geriatr Soc 2004;7:86-92. Available at: www.canadiangeriatrics.com. Accessed 2005 January 5
- Marshall SC & Malcolm Man-Son-Hing (2011) Multiple Chronic Medical Conditions and Associated Driving Risk: A Systematic Review, Traffic Injury Prevention, 12:2, 142-148, DOI: 10.1080/15389588.2010.551225
- Mathias, J., & Lucas, L. (2009). Cognitive predictors of unsafe driving in older drivers: A meta-analysis. International Psychogeriatrics, 21(4), 637-653. doi:10.1017/S1041610209009119
- McGehee, D. V., Lee, J. D., Rizzo, M., Dawson, J. and Bateman, K., 2004. Quantitative analysis of steering adaptation on a high performance fixed-base driving simulator, Transportation Research Part F: Traffic Psychology and Behaviour, 7(3), 181-196
- McKay, C., Rapport, L.J., Bryer, R.C. and Casey, J., 2011. Self-evaluation of driving simulator performance after stroke, Topics in Stroke Rehabilitation, 18(5):549-61
- McKenna, P. and Bell, V. (2007), Fitness to drive following cerebral pathology: The Rookwood Driving Battery as a tool for predicting on-road driving performance. Journal of Neuropsychology, 1: 85-100. https://doi.org/10.1348/174866407X180837 -
- McKenna, P., Jefferies, L., Dobson, A. and Frude, N. (2004), The use of a cognitive battery to predict who will fail an on-road driving test. British Journal of Clinical Psychology, 43: 325-336. https://doi.org/10.1348/0144665031752952
- Mizenko, A. J., Tefft, B. C., Arnold, L. S., & Grabowski, J. (2014). Older American drivers and traffic safety culture: A long ROAD study. Washington, DC: AAA Foundation for Traffic Safety
- Molnar F. Systematic review of the evidence for Trails B cut-off scores in assessing fitness-to-drive. Can Geriatr J 2013;16(3):120-42.PubMedGoogle Scholar
- Molnar FJ and Simpson CS (2010), 'Approach to assessing fitness to drive in patients with cardiac and cognitive conditions' Canadian Family Physician; ;56:1123-9.
- Molnar FJ, Marshall SC, Man-Son-Hing M, Wilson KG, Byszewski AM, Stiell I. Acceptability and concurrent validity of measures to predict older driver involvement in motor vehicle crashes: An Emergency Department pilot case-control study. Accident; Analysis and Prevention 2007;39(5):1056-63
- Moon S, Maud Ranchet, Mark Tant, Abiodun E. Akinwuntan, Hannes Devos (2017), 'Comparison of Unsafe Driving Across Medical Conditions' Mayo Clinic Proceedings; 92(9): 1341-1350. https://doi.org/10.1016/j.mayocp.2017.06.003- not exclusive to dementia
- Motta K, Hoe Lee, Torbjorn Falkmer (2014), 'Post-stroke driving: Examining the effect of executive dysfunction' Journal of Safety Research; 49: 33.e1-38. https://doi.org/10.1016/j.jsr.2014.02.005
- Mullen, N. W., Chattha, H. K., Weaver, B. and Bedard, M., 2008. Older driver performance on a simulator: associations between simulated tasks and cognition, Advances in Transportation Studies, Spec. Iss., p. 31-42. – awaiting article
- Northcutt Pope C, Tyler Reed Bell, Despina Stavrinos (2017), 'Mechanisms behind distracted driving behavior: The role of age and executive function in the engagement of distracted driving' Accident Analysis & Prevention; 98: 123-129. https://doi.org/10.1016/j.aap.2016.09.030
- O'Connor ML, Edwards JD, Wadley VG, Crowe M. Changes in mobility among older adults with psychometrically defined mild cognitive impairment. J Gerontol B Psychol Sci Soc Sci. 2010 May;65B(3):306-16. doi: 10.1093/geronb/gbq003.
- O'Connor, M.G., Duncanson, H. and Hollis, A.M. (2019), Use of the MMSE in the Prediction of Driving Fitness: Relevance of Specific Subtests. J Am Geriatr Soc, 67: 790-793. https://doi.org/10.1111/jgs.15772-

© 2022. Camilleri L & Whitehead D. Published online at http://www.aginganddisease.org/EN/10.14336/AD.2022.1126

SUPPLEMENTARY DATA

- O'Connor, M.G., Kapust, L.R., Lin, B., Hollis, A.M. and Jones, R.N. (2010), The 4Cs (Crash History, Family Concerns, Clinical Condition, and Cognitive Functions): A Screening Tool for the Evaluation of the At-Risk Driver. Journal of the American Geriatrics Society, 58: 1104-1108. https://doi.org/10.1111/j.1532-5415.2010.02855.x
- Okonkwo OC, GriEith HR, Vance DE, Marson DC, Ball KK, Wadley VG. Awareness of functional diEiculties in mild cognitive impairment: a multidomain assessment approach. Journal of the American Geriatric Society 2009;57(6):978-84
- Oswanski MF, Sharma OP, Raj SS, Vassar LA, Woods KL, Sargent WM, et al. Evaluation of two assessment tools in predicting driving ability of senior drivers. American Journal of Physical Medicine and Rehabilitation 2007;86(3):190-9.
- Park G. D., Allen R. W., Fiorentino D., Rosenthal T. J. and Cook M. L., 2006. Simulator Sickness Scores According to Symptom Susceptibility, Age, and Gender for an Older Driver Assessment Study, Proceedings of the Human Factors and Ergonomics Society Annual Meeting, 50(26), p. 2702-2706.
- Park SW, Eun Seok Choi, Mun Hee Lim, Eun Joo Kim, Sung Il Hwang, Kyung-In Choi, Hyun-Chul Yoo, Kuem Ju Lee, Hi-Eun Jung (2011), 'Association Between Unsafe Driving Performance and Cognitive-Perceptual Dysfunction in Older Drivers' PM&R; 3(3): 198-203. https://doi.org/10.1016/j.pmrj.2010.12.008. – non demented subjects
- Park, G. D., Cook, M. L. and Fiorentino, D., 2007. A simulator for assessing older driver skills, Advances in transportation studies, Spec. Iss., p. 71-80
- Parker D, McDonald L, Rabbitt P, et al. Elderly drivers and their accidents: the Aging Driver Questionnaire. Accid Anal Prev 2000;32:751-9.
- Patomella AH, Tham K, Johansson K, Kottorp A. P-drive on-road: internal scale validity and reliability of an assessment of on-road driving performance in people with neurological disorders. Scand J Occup Ther. 2010;17(1):86-93. doi: 10.1080/11038120903071776
- Pavlou D, Papantoniou P, Papadimitriou E, Vardaki S; Yannis G; Antoniou C; Golias J.; Papageorgiou SG (2016), 'Which are the effects of driver distraction and brain pathologies on reaction time and accident risk?' Advances in Transportation Studies; 2016(1): p83-98.
- Pavlou D, Panagiotis Papantoniou, Eleonora Papadimitriou, Sophia Vardaki, Alexandra Economou, George Yannis, Sokratis G. Papageorgiou (2017), 'Self-assessment of older drivers with brain pathologies: reported habits and self-regulation of driving' Journal of Transport & Health; 4: 90-98. https://doi.org/10.1016/j.jth.2016.08.008.
- Pavlou D and Yannis G (2018) 'Road Safety Behavior of Drivers with Neurological Diseases Affecting Cognitive Functions: An Interdisciplinary Structural Equation Model Analysis Approach' Advances in transportation studies; 44: 133-150. DOI:10.4399 / 978882551434610
- Pavlou D, Papadimitriou E, Yannis G, Papantoniou P, Golias J, Papageorgiou SG (2014), First findings from a simulator study on driving behaviour of drivers with cerebral diseases' Research Gate [online] Available from https://www.researchgate.net/profile/John-Golias/publication/261798255_First_findings_from_a_simulator_study_on_driving_behaviour_of_drivers_with_cerebral_diseases/links/0a85e535866af3a8d5000000/First-findings-from-a-simulator-study-on-driving-behaviour-of-drivers-with-cerebral-diseases.pdf. Accessed on 10th August 2021.
- Pavlou D., Papadimitriou E., Vardaki S., Antoniou C., Papantoniou P., Yannis G., Golias J., Beratis I., Economou A., Papageorgiou S.G., "Assessment of driving performance of drivers with brain pathologies in urban roads, using a driving simulator", Proceedings of the International interdisciplinary conference 'Ageing and Safe Mobility', Bergisch-Gladbach, November 2014.
- Pavlou D, Beratis I, Papantoniou P, Papadimitriou E, Golias J, Papageorgiou SG (2017), 'Driving performance profiles of drivers with brain pathologies in rural roads' International Journal of Transportation; 5(3): 17-28. http://dx.doi.org/10.14257/ijt.2017.5.3.02
- Rapoport M, Gary Naglie, Kelly Weegar, Anita Myers, Duncan Cameron, Alexander Crizzle, Nicol Korner-Bitensky, Holly Tuokko, Brenda Vrkljan, Michel Bédard, Michelle M. Porter, Barbara Mazer, Isabelle Gélinas, Malcolm Man-Son-Hing, Shawn Marshall (2013), 'The relationship between cognitive performance, perceptions of driving comfort and abilities, and self-reported driving restrictions among healthy older drivers' Accident Analysis & Prevention; 61:288-295. https://doi.org/10.1016/j.aap.2013.03.030.
- Read, Nicoleta L; Ward, Michael J; and Parkes, Andrew M. Time-to-Contact and Collision-Detection (2001), 'Estimations as Measures of Driving Safety in Old and Dementia Drivers'. In: Proceedings of the First International Driving Symposium on Human Factors in Driver Assessment, Training and Vehicle Design, 14-17 August 2001, Aspen, Colorado. Iowa City, IA: Public Policy Center, of Iowa, 2001: 240-245. https://doi.org/10.17077/drivingassessment.1046 - non dementia participants

SUPPLEMENTARY DATA

- Regan MA, Hallett C, Gordon CP. Driver distraction and driver inattention: definition, relationship and taxonomy. Accid Anal Prev. 2011 Sep;43(5):1771-81. doi: 10.1016/j.aap.2011.04.008.
- Roe CM, https://www.sciencedirect.com/science/article/pii/S2352873716300476 -
- Ronen, A. and Yair, N., 2013. The adaptation period to a driving simulator, Transportation research part F: traffic psychology and behaviour, 18, p. 94-106
- Ross LA, Anstey KJ, Kiely KM, Windsor TD, Byles JE, Luszcz MA, Mitchell P. Older drivers in Australia: trends in driving status and cognitive and visual impairment. Journal of the American Geriatric Society 2009;57(10):1868-73
- Roy M, Molnar F. Systematic review of the evidence for Trails B cut-off scores in assessing ftness-todrive. Can Geriatr J 2013;16(3):120-42.
- Sahami, S. and Sayed, T., 2013. How drivers adapt to drive in driving simulator, and what is the impact of practice scenario on the research?, Transportation Research Part F: Traffic Psychology and Behaviour, 16, p. 41-52.
- Samuelsson K, Wressle E (2020), 'Decisions on driving after brain injury/disease: Feasibility and construct validity of a new simulator assessment tool' British Journal of Occupational Therapy; 84(7): 421-429. https://doi.org/10.1177/0308022620950986
- Samuelsson, K., Modig-Arding, I., & Wressle, E. (2018). Driving after an injury or disease affecting the brain: an analysis of clinical data. British Journal of Occupational Therapy, 81, 376 - 383.
- Sawada, T., Tomori, K., Hamana, H., Ohno, K., Seike, Y., Igari, Y., & Fujita, Y. (2019). Reliability and validity of on-road driving tests in vulnerable adults: a systematic review. International journal of rehabilitation research. Internationale Zeitschrift fur Rehabilitationsforschung. Revue internationale de recherches de readaptation, 42(4), 289–299. https://doi.org/10.1097/MRR.0000000000000374
- Selander H (2012), 'Driving assessment and driving behaviour' Jönköping: School of Health Sciences , 2012. , p. 100. https://www.diva-portal.org/smash/record.jsf?pid=diva2%3A570186&dswid=-3641
- Selander H, Ewa Wressle & Kersti Samuelsson (2020) Cognitive prerequisites for fitness to drive: Norm values for the TMT, UFOV and NorSDSA tests, Scandinavian Journal of Occupational Therapy, 27:3, 231-239, DOI: 10.1080/11038128.2019.1614214
- Selander H, Hoe C. Lee, Kurt Johansson, Torbjörn Falkmer (2011), 'Older drivers: On-road and off-road test results' Accident Analysis & Prevention; 43(4): 1348-1354. https://doi.org/10.1016/j.aap.2011.02.007.
- Sharma N, Azizuddin Khan, Jai Prakash Kushvah (2020), 'Role of Prospective Memory in Driving Behaviour' Transportation Research Procedia; 48: 1334-1341. https://doi.org/10.1016/j.trpro.2020.08.162
- Shechtman O, Kezia D. Awadzi, Sherrilene Classen, Desiree N. Lanford, Yongsung Joo; Validity and Critical Driving Errors of On-Road Assessment for Older Drivers. Am J Occup Ther 2010;64(2):242–251. https://doi.org/10.5014/ajot.64.2.242
- Shechtman, O., 2010. Validation of driving simulators, Advances in Transportation Studies, p. 53-62
- Shechtman, O., Classen, S., Awadzi, K. and Mann, W., 2009. Comparison of driving errors between on-the-road and simulated driving assessment: A validation study, Traffic Injury Prevention, 10(4), p. 379-385
- Shimada, H., Tsutsumimoto, K., Lee, S., Doi, T., Makizako, H., Lee, S., Harada, K., Hotta, R., Bae, S., Nakakubo, S., Uemura, K., Park, H., and Suzuki, T. (2016) Driving continuity in cognitively impaired older drivers. Geriatrics & Gerontology International, 16: 508– 514. doi: 10.1111/ggi.12504.
- Siren A, Meng A. Cognitive screening of older drivers does not produce safety benefits. Accident; Analysis and Prevention 2012;45:634-8.
- Soderstorm CA, Scottino MA, Joyce JJ, Burch C, Ho SM, Kerns TJ. Police referral of drivers to the Maryland Motor Vehicle Administration's Medical Advisory Board. Annals of Advances in Automotive Medicine 2009;53:105-16
- Spinney JEL, Scott DM, Newbold KB. Transport mobility benefits and quality of life: a time-use perspective of elderly Canadians. Transport Policy 2009;16(1):1-11
- Stack AH, Duggan O, Stapleton T (2018), 'Assessing fitness to drive after stroke: A survey investigating current practice among occupational therapists in Ireland' Irish Journal of Occupational Therapy; 46(2): 106-129. DOI 10.1108/IJOT-03-2018-0006
- Staplin L, Gish KW, Wagner EK. MaryPODS revisited: updated crash analysis and implications for screening program implementation. Journal of Safety Research 2003;34(4):389-97
- Staplin L, Kenneth W. Gish, Kathy H. Lococo, John J. Joyce, Kathy J. Sifrit (2013), 'The Maze Test: A significant predictor of older driver crash risk' Accident Analysis & Prevention; 50: 483-489. https://doi.org/10.1016/j.aap.2012.05.025.
- Staplin L, Lococo KH, Gish KW, Decina LE. Model driver screening and evaluation program final technical report: Vol. 2. Maryland Pilot Older Driver Study. www.nhtsa.gov/people/ injury/olddrive/modeldriver/volume_ii.htm (accessed 2 April 2013).

SUPPLEMENTARY DATA

- Staplin L., 2010. Safe Driving Tactics: What Role for Low Cost Simulation?. TRB Annual Meeting
- Stoner, A.H., Fischer, L. D. and Mollenhauer, Jr. M., 2011. Simulator and Scenario Factors Influencing Simulator Sickness. Handbook of Driving Simulation for Engineering, Medicine and Psychology, CRC Press
- Stutts JC, Wilkins JW. On-road driving evaluations: a potential tool for helping older adults drive safely longer. Safety Research 2003;34(4):431-9
- Sun, Q.(., Xia, J.(., Foster, J. et al. A psycho-Geoinformatics approach for investigating older adults' driving behaviours and underlying cognitive mechanisms. Eur. Transp. Res. Rev. 10, 36 (2018). https://doi.org/10.1186/s12544-018-0308-6
- Szlyk, J.P., Myers, L., Zhang,Y.X.,Wetzel, L., & Shapiro, R. (2002). Development and assessment of a neuropsychological battery to aid in predicting driving performance. Journal of Rehabilitation Research and Development, 3(4): 483-496. https://www.rehab.research.va.gov/jour/02/39/4/pdf/myers.pdf –
- Tan, K.M., O'Driscoll, A. & O'Neill, D. Factors affecting return to driving post-stroke. Ir J Med Sci 180, 41–45 (2011). https://doi.org/10.1007/s11845-010-0528-9
- Tiu J, Harmon AC, Stowe JD, Zwa A, Kinnear M, Dimitrov L, Nolte T, Carr DB. Feasibility and Validity of a Low-Cost Racing Simulator in Driving Assessment after Stroke. Geriatrics. 2020; 5(2):35. https://doi.org/10.3390/geriatrics5020035
- Vanlaar WGM, Marisela Mainegra Hing, Shawna Meister, Jan-Michael Charles, Leanna Ireland, Dan Mayhew, David Carr, Peggy Barco, Robyn D. Robertson (2019), 'Pilot study of a new road test to assess cognitive fitness to drive' Transportation Research Part F: Traffic Psychology and Behaviour; 65: 258-267. https://doi.org/10.1016/j.trf.2019.07.022.
- Vaughan L, Hogan P, Rapp SR, Dugan E, Marottoli RA, Snively BM, Shumaker SA, Sink KA (2015), 'Driving with Mild Cognitive Impairment or Dementia: Cognitive Test Performance and Proxy Report of Daily Life Function in Older Women' J Am Geriatr Soc 63: 1774– 1782.
- Wald JL, Liu L, and Reil S (2000), 'Concurrent Validity of a Virtual Reality Driving Assessment for Persons with Brain Injury' CyberPsychology & Behavior 2000 3:4, 643-654.
- Wernham, M., Jarrett, P. G., Stewart, C., MacDonald, E., MacNeil, D., & Hobbs, C. (2014). Comparison of the SIMARD MD to Clinical Impression in Assessing Fitness to Drive in Patients with Cognitive Impairment. Canadian geriatrics journal : CGJ, 17(2), 63–69. https://doi.org/10.5770/cgj.17.100-
- Whelihan W, DiCarlo MA, Paul RH (2005), 'The relationship of neuropsychological functioning to driving competence in older persons with early cognitive decline' Archives of Clinical Neuropsychology; 20(2): 217–228, https://doi.org/10.1016/j.acn.2004.07.002-
- White JH, Belinda Miller, Parker Magin, John Attia, Jonathan Sturm & Michael Pollack (2012) Access and participation in the community: a prospective qualitative study of driving post-stroke, Disability and Rehabilitation, 34:10, 831-838, DOI: 10.3109/09638288.2011.623754
- Wong IY, Simon S. Smith, Karen A. Sullivan (2012), 'The relationship between cognitive ability, insight and self-regulatory behaviors: Findings from the older driver population' Accident Analysis & Prevention; 49: 316-321. https://doi.org/10.1016/j.aap.2012.05.031.
- Wood JM, Mark S. Horswill, Philippe F. Lacherez, Kaarin J. Anstey (2013), 'Evaluation of screening tests for predicting older driver performance and safety assessed by an on-road test' Accident Analysis & Prevention; 50: 1161-1168. https://doi.org/10.1016/j.aap.2012.09.009.
- Woolf C, Heng K, Pan S, Layde MP, Zhu S. The elderly and motor vehicle crashes. Chinese Journal of Emergency Medicine 2006;15(1):6-12. [ISSN: 1671-0282}
- Woolnough, A., Salim, D., Marshall, S. C., Weegar, K., Porter, M. M., Rapoport MJ, Vrkljan, B. (2013). Determining the validity of the AMA guide: A historical cohort analysis of the assessment of driving related skills and crash rate among older drivers. Accident; Analysis and Prevention, 61, 311-316. doi:10.1016/j.aap.2013.03.020
- Zingg, C., Puelschen, D. & Soyka, M. Neuropsychological assessment of driving ability and self-evaluation: a comparison between driving offenders and a control group. Eur Arch Psychiatry Clin Neurosci 259, 491 (2009). https://doi.org/10.1007/s00406-009-0019-z
- Zook NA, Thomas L. Bennett & Marlis Lane (2009) Identifying At-Risk Older Adult Community-Dwelling Drivers Through Neuropsychological Evaluation, Applied Neuropsychology, 16:4, 281-287, DOI: 10.1080/09084280903297826
- Edwards JD, Lunsman M, Perkins M, Rebok GW, Roth DL (2009) Driving cessation and health trajectories in older adults. J Gerontol A Biol Sci Med Sci 64(12):1290–1295. doi:10.1093/gerona/glp114
- Langford J, Bohensky M, Koppel S, Newstead S (2008) Do agebased mandatory assessments reduce older drivers' risk to other road users? Accid Anal Prev 40(6):1913–1918. doi:10.1016/j.aap.2008.08.010

© 2022. Camilleri L & Whitehead D. Published online at http://www.aginganddisease.org/EN/10.14336/AD.2022.1126

- Woolnough, A., Salim, D., Marshall, S. C., Weegar, K., Porter, M. M., Rapoport, M. J., …, Vrkljan, B. (2013). Determining the validity of the AMA guide: A historical cohort analysis of the assessment of driving related skills and crash rate among older drivers. Accident; Analysis and Prevention, 61, 311-316. doi:10.1016/j.aap.2013.03.020

7. Not related to driving assessment and/or behaviour in dementia (n= 71)

- Arms, T. (2016). The NPs role of assessing and intervening with older adult drivers. Nursing Research and Practice. doi:10.1155/2016/3254857
- Ashendorf L, Michael L Alosco, Hanaan Bing-Canar, Kimberly R Chapman, Brett Martin, Christine E Chaisson, Diane Dixon, Eric G Steinberg, Yorghos Tripodis, Neil W Kowall, Robert A Stern, Clinical Utility of Select Neuropsychological Assessment Battery Tests in Predicting Functional Abilities in Dementia, Archives of Clinical Neuropsychology, Volume 33, Issue 5, August 2018, Pages 530–540, https://doi.org/10.1093/arclin/acx100
- Bhalla RK, Papandonatos GD, Stern RA, Ott BR. Anxiety of Alzheimer's disease patients before and after a standardized on-road driving test. Alzheimers Dement. 2007 Jan;3(1):33-9. doi: 10.1016/j.jalz.2006.10.006.
- Bonnie H (2016), 'Developing an evidenced-based role for the social worker in addressing driving competency in clients with dementia' ProQuest [online] Available from https://www.proquest.com/openview/df59b6e3d434260ea5440e003e6571a4/1?pq-origsite=gscholar&cbl=18750. Accessed on 4th August 2021.
- Campos JL, Bédard M, Classen S, Delparte JJ, Hebert DA., Hyde N, Law G, Naglie G, Yung S (2017) 'Guiding Framework for Driver Assessment Using Driving Simulators' Frontiers in Psychology; 8: 1428. DOI=10.3389/fpsyg.2017.01428
- Cochrane Central Register of Controlled Trials: 'A driving in dementia decision tool: preliminary analysis' Rapoport MJ, Sarracini CZ, Rozmovits L, Kiss A, Grigoriev I, Taylor R, Herrmann N, Mulsant BH, Cameron D, Frank C, Seitz D, Byszewski A, Tang-Wai DF, Masellis M, Molnar F, Naglie G; Alzheimer's & dementia, 2016, 12(7), P789
- Croston J, Meuser TM, Berg-Weger M, Grant EA, Carr DB. Driving Retirement in Older Adults with Dementia. Top Geriatr Rehabil. 2009;25(2):154-162. doi:10.1097/TGR.0b013e3181a103fd
- Daiello LA, Ott BR, Festa EK, Friedman M, Miller LA, Heindel WC. EEects of cholinesterase inhibitors on visual attention in drivers with Alzheimer disease. Journal of Clinical Psychopharmacology 2010;30(3):245-51.
- Davis J, Hamann C, Butcher B, Peek-Asa C (2020), 'The Medical Referral Process and Motor-Vehicle Crash Risk for Drivers with Dementia' Geriatrics 2020, 5, 91; doi:10.3390/geriatrics5040091
- Delaying the Progression of Driving Impairment in Individuals With Mild Alzheimer's Disease, NCT00476008, https://clinicaltrials.gov/show/NCT00476008, 2007
- Delaying the progression of driving impairment in individuals with mild Alzheimer's disease or delaying the progression of driving impairment in individuals with mild Alzheimer's disease. Clinicaltrials.gov [http://clinicaltrials.gov], 2007
- Dobbs, Bonnie M. PhD; Harper, Lori A. PhD; Wood, Ava MSc Transitioning From Driving to Driving Cessation, Topics in Geriatric Rehabilitation: January 2009 - Volume 25 - Issue 1 - p 73-86 doi: 10.1097/01.TGR.0000346058.32801.95
- Fraade-Blanar LA, Ryan N. Hansen, Kwun Chuen G. Chan, Jeanne M. Sears, Hilaire J. Thompson, Paul K. Crane, Beth E. Ebel (2018), 'Diagnosed dementia and the risk of motor vehicle crash among older driverS' Accident Analysis & Prevention; 113: 47-53. https://doi.org/10.1016/j.aap.2017.12.021.
- Fraade-Blannar L and Smith JP (2018), 'Cognitive Change and Driving Behavior among Older Drivers' Transportation Research Record: Journal of the Transportation Research Board; Volume: 2672 issue: 33, page(s): 89-100. https://doi.org/10.1177/0361198118801356
- Gergerich EM (2016), 'Reporting Policy Regarding Drivers with Dementia' The Gerontologist; 56(2): 345–356, https://doi.org/10.1093/geront/gnv143.
- Gorrie CA, Julie Brown, Phil M.E. Waite (2008), 'Crash characteristics of older pedestrian fatalities: Dementia pathology may be related to 'at risk' traffic situations' Accident Analysis & Prevention; 40(3): 912-919. https://doi.org/10.1016/j.aap.2007.10.006.
- Gorrie CA, Rodriguez M, Sachdev P, Duflou J, Waite PM. Mild neuritic changes are increased in the brains of fatally injured older motor vehicle drivers. Accid Anal Prev. 2007 Nov;39(6):1114-20. doi: 10.1016/j.aap.2007.02.008.
- Grabowski DC, Campbell CM, Morrisey MA. Elderly licensure laws and motor vehicle fatalities. JAMA 2004;291:2840 –2846.

SUPPLEMENTARY DATA

- Harries, P., Unsworth, C., Gokalp, H. et al. A randomised controlled trial to test the effectiveness of decision training on assessors' ability to determine optimal fitness-to-drive recommendations for older or disabled drivers. BMC Med Educ 18, 27 (2018). https://doi.org/10.1186/s12909-018-1131-4
- Herrmann N, Rapoport MJ, Sambrook R, Hébert R, McCracken P, Robillard A (2006), 'Predictors of driving cessation in mild-to-moderate dementia' CMAJ; 175 (6) 591-595; DOI: https://doi.org/10.1503/cmaj.051707.
- Holland PJ, Tappen RM, Fisher L, Curtis AL, Apter J, 'Effect of memantine on the progression of driving impairment in patients with mild Alzheimer's disease' Journal of nutrition, health & aging, 2013, 17(9), 823-824
- Hopkins RW, Kilik L, Day DJ, Rows C, Tseng H. Driving and dementia in Ontario: a quantitative assessment of the problem. Can J Psychiatry. 2004 Jul;49(7):434-8. doi: 10.1177/070674370404900704.
- Jett K, Ruth M. Tappen, Monica Rosselli (2005), 'Imposed versus involved: Different strategies to effect driving cessation in cognitively impaired older adults' Geriatric Nursing; 26(2): 111-116. https://doi.org/10.1016/j.gerinurse.2005.01.004.
- Kelly, E., Darke, S., & Ross, J. (2004). A review of drug use and driving: Epidemiology, impairment, risk factors and risk perceptions. Drug and Alcohol Review, 23, 319–344.
- Klöppel S. Brain morphometry and functional imaging techniques in dementia: methods, findings and relevance in forensic neurology. Curr Opin Neurol. 2009 Dec;22(6):612-6. doi: 10.1097/WCO.0b013e328332ba0f.
- Liddle, J., Bennett, S., Allen, S., Lie, D., Standen, B., & Pachana, N. (2013). The stages of driving cessation for people with dementia: Needs and challenges. International Psychogeriatrics, 25(12), 2033-2046. doi:10.1017/S1041610213001464
- Lopez A, Caffò AO, Tinella L, Postma A, Bosco A. Studying Individual Differences in Spatial Cognition Through Differential Item Functioning Analysis. Brain Sciences. 2020; 10(11):774. https://doi.org/10.3390/brainsci10110774
- Martyr A, Clare L (2012), 'Executive Function and Activities of Daily Living in Alzheimer's Disease: A Correlational Meta-Analysis' Dement Geriatr Cogn Disord; 33:189–203. https://doi.org/10.1159/000338233
- Meuleners, L. and Ng, J. and Chow, K. and Stevenson, M. 2016. Motor vehicle crashes and dementia: A population-based study. American Geriatrics Society Journal. 64 (5): pp. 1039-1045, http://doi.org/10.1111/1467-8462.12164. T
- Mizuno Y., Arai A. & Arai Y. (2008) Determination of driving cessation for older adults with dementia in Japan. International Journal of Geriatric Psychiatry 23(9), 987–989.
- Molnar FJ, Patel A, Marshall SC, Man-Son-Hing M, Wilson KG. Systematic review of the optimal frequency of follow-up in persons with mild dementia who continue to drive. Alzheimer Dis Assoc Disord. 2006 Oct-Dec;20(4):295-7. doi: 10.1097/01.wad.0000213843.43871.c7.
- Naglie G, Elaine Stasiulis, Stephanie Yamin, Brenda Vrkljan, Holly Tuokko, Sarah Sanford, Michelle Porter, Jan Polgar, Anita Myers, Paige Moorhouse, Frank Molnar, Barbara Mazer, Shawn Marshall, Isabelle Gelinas, Alexander Crizzle, Anna Byszewski, Patricia Belchior, Michael Bedard, Mark J. Rapoport (2019), 'DRIVING CESSATION IN DEMENTIA: A HOT TOPIC THAT IS STILL AVOIDED' Alzheimer's & Dementia; 15(7): P858-P859. https://doi.org/10.1016/j.jalz.2019.06.3020.
- Naglie, G, Sanford, S, Cameron, D, et al. Strategies to facilitate driving decision-making and the transition to non-driving in older adults and persons with dementia: a mixed methods scoping review (abstract). J Am Geriatr Soc 2016; 64: S80
- O'Connor ML, Jerri D. Edwards, Yvonne Bannon (2013), 'Self-rated driving habits among older adults with clinically-defined mild cognitive impairment, clinically-defined dementia, and normal cognition' Accident Analysis & Prevention; 61:197-202. https://doi.org/10.1016/j.aap.2013.05.010
- O'Brien HL, Tetewsky SJ, Avery LM, Cushman LA, Makous W, Duffy CJ. Visual mechanisms of spatial disorientation in Alzheimer's disease. Cereb Cortex. 2001 Nov;11(11):1083-92. doi: 10.1093/cercor/11.11.1083.
- Ott, B.R., Papandonatos, G.D., Burke, E.M., Erdman, D., Carr, D.B. and Davis, J.D. (2020), A video feedback intervention for cognitively impaired older drivers. Alzheimer's Dement., 16: e041852. https://doi.org/10.1002/alz.041852
- Paire-Ficout L, Marin-Lamellet C, Lafont S, Thomas-Antérion C, Laurent B. The role of navigation instruction at intersections for older drivers and those with early Alzheimer's disease. Accid Anal Prev. 2016 Nov;96:249-254. doi: 10.1016/j.aap.2016.08.013.
- Pastor, Diane K. PhD, MBA, NP-C; Arms, Tamatha DNP, PMHNP-BC, NP-C; Jones, Andrea L. PhD, MSW Red Flags and Off Ramps, Home Healthcare Now: September/October 2018 - Volume 36 - Issue 5 - p 319-323 doi: 10.1097/NHH.0000000000000695

© 2022. Camilleri L & Whitehead D. Published online at http://www.aginganddisease.org/EN/10.14336/AD.2022.1126

SUPPLEMENTARY DATA

- Perkinson MA, Berg-Weger ML, Carr DB, Meuser TM, PalmeR JL, Buckles VD, Powlishta KK, Foley DJ, Morris JC (2005) 'Driving and Dementia of the Alzheimer Type: Beliefs and Cessation Strategies Among Stakeholders' The Gerontologist, Volume 45, Issue 5, October 2005, Pages 676–685, https://doi.org/10.1093/geront/45.5.676
- Petersen JD, Siersma VD, Depont Christensen R, Storsveen MM, Nielsen CT, Vass M, Waldorff FB. Dementia and road traffic accidents among non-institutionalized older people in Denmark: A Danish register-based nested case-control study. Scand J Public Health. 2019 Mar;47(2):221-228. doi: 10.1177/1403494818782094
- Pievani M, Willem de Haan, Tao Wu, William W Seeley, Giovanni B Frisoni (2011), 'Functional network disruption in the degenerative dementias' The Lancet Neurology; 10(9): 829-843. https://doi.org/10.1016/S1474-4422(11)70158-2.
- Poletti M, Enrici I, Adenzato M. Cognitive and affective Theory of Mind in neurodegenerative diseases: neuropsychological, neuroanatomical and neurochemical levels. Neurosci Biobehav Rev. 2012 Oct;36(9):2147-64. doi: 10.1016/j.neubiorev.2012.07.004.
- Rapoport MJ, Cameron DH, Sanford S, Naglie G; Canadian Consortium on Neurodegeneration in Aging Driving and Dementia Team. A systematic review of intervention approaches for driving cessation in older adults. Int J Geriatr Psychiatry. 2017 May;32(5):484-491. doi: 10.1002/gps.4681.
- Rapoport MJ, Herrmann N, Molnar FJ, et al. Psychotropic medications and motor vehicle collisions in patients with dementia. J Am Geriatric Soc 2008;56:1968-70.
- Rapoport MJ, Zucchero Sarracini C, Kiss A, Lee L, Byszewski A, Seitz DP, Vrkljan B, Molnar F, Herrmann N, Tang-Wai DF, Frank C, Henry B, Pimlott N, Masellis M, Naglie G (2018), 'Computer-Based Driving in Dementia Decision Tool With Mail Support: Cluster Randomized Controlled Trial.' J Med Internet Res.; 20(5):e194. doi: 10.2196/jmir.9126.
- Redelmeier, DA, Vinkatesh, V, Stanbrook, MB. Mandatory reporting by physicians of patients potentially unfit to drive. Open Med 2008; 2(1): e8–e17.
- Sachdev PS, Blacker D, Blazer DG, Ganguli M, Jeste DV, Paulsen JS, Petersen RC. Classifying neurocognitive disorders: the DSM-5 approach. Nat Rev Neurol. 2014 Nov;10(11):634-42. doi: 10.1038/nrneurol.2014.181. Epub 2014 Sep 30. PMID: 25266297.
- Sanford S, Rapoport MJ, Tuokko H, Crizzle A, Hatzifilalithis S, Laberge S, Naglie G; Canadian Consortium on Neurodegeneration in Aging Driving and Dementia Team. Independence, loss, and social identity: Perspectives on driving cessation and dementia. Dementia (London). 2019 Oct-Nov;18(7-8):2906-2924. doi: 10.1177/1471301218762838.
- Seiler S, Schmidt H, Lechner A, Benke T, Sanin G, et al. (2012) Driving Cessation and Dementia: Results of the Prospective Registry on Dementia in Austria (PRODEM). PLOS ONE 7(12): e52710. https://doi.org/10.1371/journal.pone.0052710
- Sims J, Rouse-Watson S, Schattner P, Beveridge A, Jones KM (2011), 'To Drive or Not to Drive: Assessment Dilemmas for GPs' International Journal of Family Medicin; Volume 2012, Article ID 417512, 6 pages. doi:10.1155/2012/417512
- Sinnott C, Foley T, Forsyth J, McLoughlin K, Horgan L, Bradley CP (2018) Consultations on driving in people with cognitive impairment in primary care: A scoping review of the evidence. PLoS ONE 13(10): e0205580. https://doi.org/10.1371/journal.pone.0205580
- Snellgrove, C.A. and Hecker, J.R. (2002), Driving and dementia: General practitioner attitudes, knowledge and self-reported clinical practices in South Australia. Australasian Journal on Ageing, 21: 210-212. https://doi.org/10.1111/j.1741-6612.2002.tb00449.x
- Snyder KM, Ganzini L. Outcomes of Oregon's law mandating physician reporting of impaired drivers. Journal of Geriatric Psychiatry and Neurology 2009;22(3):161-5
- Talbot A, Irene Bruce, Conal J. Cunningham, Robert F. Coen, Brian A. Lawlor, Davis Coakley, J. B. Walsh, Desmond O'Neill, Driving cessation in patients attending a memory clinic, Age and Ageing, Volume 34, Issue 4, July 2005, Pages 363–368, https://doi.org/10.1093/ageing/afi090
- Taylor BD, Tripodes S. The eEects of driving cessation on the elderly with dementia and their caregivers. Accident; Analysis and Prevention 2001;33(4):519-28.
- Tippet WJ, Sergio LE, Black SE (2012), 'Compromised visually guided motor control in individuals with Alzheimer's disease: Can reliable distinctions be observed?' Journal of Clinical Neuroscience; 19(5): 655-660. https://doi.org/10.1016/j.jocn.2011.09.013
- Valcour V.G., Masaki K.H. & Blanchette P.L. (2002) Self-reported driving, cognitive status and physician awareness of cognitive impairment. Journal of the American Geriatrics Society 50(7), 1265–1267.

SUPPLEMENTARY DATA

- Velayudhan L.a,b · Baillon S.b,c · Urbaskova G.a · McCulloch L.b · Tromans S.c · Storey M.b · Lindesay J.b · Bhattacharyya S. (2018), 'Driving Cessation in Patients Attending a Young-Onset Dementia Clinic: A Retrospective Cohort Study' Dement Geriatr Cogn Disord Extra 2018;8:190–198. https://doi.org/10.1159/000488237
- Yamin S, Naglie G, Manouia R, Sanford S, Stasiulis E, Rapoport MJ (2021), 'The Role of Gender in the Transition to Driving Cessation in Persons with Dementia' The American Journal of Geriatric Psychiatry; 29(4): S120-S121. https://doi.org/10.1016/j.jagp.2021.01.115 – experience of driving cessation
- Yamin S, Stinchcombe A, Gagnon s (2015), 'Driving Competence in Mild Dementia with Lewy Bodies: In Search of Cognitive Predictors Using Driving Simulation' International journal pf Alzheimer's disease; vol. 2015, Article ID 806024, 8 pages, 2015. https://doi.org/10.1155/2015/806024
- Yamin S, Stinchcombe A, Gagnon S (2016), 'Comparing Cognitive Profiles of Licensed Drivers with Mild Alzheimer's Disease and Mild Dementia with Lewy Bodies' International Journal of Alzheimer's Disease; Volume 2016, Article ID 6542962, 11 pages. http://dx.doi.org/10.1155/2016/6542962
- Yi J. · Lee H.C.-Y. · Parsons R. · Falkmer T. (2015), 'The Effect of the Global Positioning System on the Driving Performance of People with Mild Alzheimer's Disease' Gerontology; 61:79-88. https://doi.org/10.1159/000365922
- Petersen JD, Siersma VD, Depont Christensen R, Storsveen MM, Nielsen CT, Vass M, Waldorff FB. Dementia and road traffic accidents among non-institutionalized older people in Denmark: A Danish register-based nested case-control study. Scand J Public Health. 2019 Mar;47(2):221-228. doi: 10.1177/1403494818782094
- Pievani M, Willem de Haan, Tao Wu, William W Seeley, Giovanni B Frisoni (2011), 'Functional network disruption in the degenerative dementias' The Lancet Neurology; 10(9): 829-843. https://doi.org/10.1016/S1474-4422(11)70158-2.
- Rapoport MJ, Herrmann N, Molnar FJ, et al. Psychotropic medications and motor vehicle collisions in patients with dementia. J Am Geriatric Soc 2008;56:1968-70
- Spiers HJ, Maguire EA (2007) Neural substrates of driving behaviour. Neuroimage 36(1):245–255. doi:10.1016/j.neuroimage.2007.02.032
- Ragland DR, Satariano WA, MacLeod KE (2005) Driving cessation and increased depressive symptoms. J Gerontol A Biol SciMed Sci 60(3):399–403
- Caragata Nasvadi G, Wister A (2009) Do restricted driver's licenses lower crash risk among older drivers? A survival analysis of insurance data from British Columbia. Gerontologist 49(4):474–484. doi:10.1093/geront/gnp039
- Langford J, Methorst R, Hakamies-Blomqvist L (2006) Older drivers do not have a high crash risk—a replication of low mileage bias. Accid Anal Prev 38(3):574–578. doi:10.1016/j. aap.2005.12.002
- Yamasaki T, Tobimatsu S (2018), 'Driving Ability in Alzheimer Disease Spectrum: Neural Basis, Assessment, and Potential Use of Optic Flow Event-Related Potentials' Front. Neurol. 9:750. doi: 10.3389/fneur.2018.00750
- Grabowski DC, Campbell CM, Morrisey MA. Elderly licensure laws and motor vehicle fatalities. JAMA 2004;291:2840 –2846

B. Standerdised Driving Assessments

Washington University Road Test (WURT)

Standardised Road test consisting of the following components: (Grace J, Amick M, D'Abreu A, et.al., 2005)

Operational
1. Appropriate reaction to merging traffic
2. Awareness of how driving is affecting others
3. Lane change
4. Awareness of traffic environment
5. Left turn at four-way stop

Strategic
1. Patient's reasoning about making a left-hand turn onto a one-way street
2. Ability to follow a lengthy command
3. Overall judgment

Tactical
6. Merging from right: scanned for lane change
7. Lane change: checks blind spot
8. Lane change: smoothness of change
9. Left turn turns in appropriate lane
10. Pulls over to curb
11. Lane change: signals
12. Checks mirrors
13. Right turn: observed legal right on red
14. Right at four-way stop: complete stop
15. Right turn: signals

© 2022. Camilleri L & Whitehead D. Published online at http://www.aginganddisease.org/EN/10.14336/AD.2022.1126

4. Lapses of concentration

16. Parking: checks traffic backing out of space
Drives within 5 mph of the speed limit

Rhode Island Road Test (RIRT)

Adaptation of the WURT and consists of the following items: (Ott BR et.al., 2012)

1. Awareness of signs
2. Light awareness
3. Approaches intersection at appropriate speed
4. Brakes smoothly and accurately
5. Comes to a complete stop
6. Aware of traffic situations
7. Responds appropriately to signal
8. Scans
9. Uses mirrors
10. Positions for turn
11. Yields right of way
12. Proceeds timely
13. Accelerates smoothly and accurately
14. Signal use
15. Attends to task
16. Awareness of pedestrians
17. Spatial awareness
18. Lane selection
19. Lane keeping
20. Awareness of environment
21. Speed control
22. Steering control
23. Uses mirrors for lane change
24. Checks blind spots
25. Changes lanes without verbal cue
26. Scans while in motion
27. Shifts for park
28. Turns vehicle engine off

Test Ride Investigating Practical (TRIP)

Standardised on-road assessment consisting of the following items: (de Haan GA, Melis-Dankers BJ, Brouwer WH et.al., 2014)

Position on the Road

1. What is the average lateral positioning on the driving lane (on a regular two-lane road)? (Too much to the left, approximately in the middle, too much to the right, fluctuating)

How is the steadiness of steering (swaying and drifting away)?

On Straight Roads

2. speed < 50 km/h
3. speed > 50 km/h

In Curves

4. speed < 50 km/h
5. speed > 50 km/h

When Making Head/Eye Movements

6. speed < 50 km/h
7. speed > 50 km/h

How good is the choice of position for the following specific situations?

8. Lane choice for straight ahead
9. Lane choice for turning right
10. Lane choice for turning left
11. Lane choice for/at roundabouts

Car Following Distance

1. How would you classify the style of car following of the driver? (short, sufficient, long)

How well is the following distance adapted to variations of speed of the cars ahead?

13. In town areas

© 2022. Camilleri L & Whitehead D. Published online at http://www.aginganddisease.org/EN/10.14336/AD.2022.1126

14. Outside town areas

Choice of Speed

2. How would you classify the driver in terms of his choice of speed? (average, slow, fast)

How good is the driver’s adaptation of speed to the circumstances?

16. In town areas
17. Outside town areas

Observation Behavior (Head and Eye Movements)

General

18. When moving straight ahead
19. Crossing junctions without designated priorities
20. Crossing priority junctions
21. When turning right at junctions or forks
22. When turning left at junctions or forks
23. In curves 24. When using inside mirror
25. When using outside mirror
26. Observation in the blind angle

Anticipatory viewing behaviour

27. With regard to changing road situations
28. With regard to changing traffic situations

Traffic Signals (Lights and Signs)

29. Perception
30. Reaction

Overtaking and Passing By

31. Perception and judgement
32. Performing the manoeuvres

Anticipation

(At a tactical level, e.g., slowing down when a pedestrian approaches the driving lane)

33. With regard to changing road situations
34. With regard to changing traffic situations

Communication With and Adaptation to Other Traffic Participants

35. With other car drivers
36. With cyclists and pedestrians

Assessment of Specific Situations

A. Turning left on a priority road or no traffic lights.

When Approaching the Junction

37. Adaptation of speed 38. Use of mirrors and looking sideways
39. Operating the direction indicator
40. Position on the driving lane
41. Viewing behaviour (head movements)
42. Effectiveness of viewing behaviour (seeing other traffic)

© 2022. Camilleri L & Whitehead D. Published online at http://www.aginganddisease.org/EN/10.14336/AD.2022.1126

SUPPLEMENTARY DATA

At the Junction
43. Choice of position
44. Viewing behaviour (head movements)
45. Effectiveness of viewing behaviour (seeing other traffic)
46. Application of the priority rules
47. Tempo of perception and action

B. Merging with a fast-moving stream of traffic (merging lane trunk road or motorway).
48. Acceleration on the merging lane
49. Looking sideways
50. Adaptation of speed to other traffic
51. Operating the direction indicator
52. Driving on to the main lane

Mechanical Operation
53. Operating the accelerator
54. Operating the brakes

General Impressions
55. Practical fitness to drive (general)
56. Mechanical operation
57. Traffic perception and traffic insight

The Composite Driving Assessment Scale (CDAS)

A driving assessment tool developed by Davis JD, Papandonatos GD, Miller LA et.al. (2012) to assess naturalistic driving behaviour in patients with dementia. It consists of global events (attention, attitude and reaction time) and discrete events which include the following items:

1. Checks blind spots
2. Makes complete stops
3. Pays attention
4. Awareness of driving on others
5. Responds to signs
6. Lane keeping
7. Appropriate response to emergency vehicles
8. Traffic awareness
9. Right turn
10. Speed control

Mockingbird Event Scoring System

Originally developed by a commercial driving evaluation system. It was used in the study conducted by Davis JD, Wang S, Festa EK et.al. (2018) and consists of the following:

1. Unsafe/risky behaviours
2. Distracted by electronic Device
3. Unsafe/unnecessary behaviours
4. Minor lane maintenance error
5. Other distraction
6. Failed to keep an out
7. Distracted by mobile usage
8. Not looking far enough ahead

© 2022. Camilleri L & Whitehead D. Published online at http://www.aginganddisease.org/EN/10.14336/AD.2022.1126
